# Dolabellane Diterpenoids from Soft Coral *Clavularia viridis* with Anti-Inflammatory Activities

**DOI:** 10.3390/md23080312

**Published:** 2025-07-30

**Authors:** Chufan Gu, Hongli Jia, Kang Zhou, Bin Wang, Wenhan Lin, Wei Cheng

**Affiliations:** 1 State Key Laboratory of Natural and Biomimetic Drugs, Peking University, Beijing 100191, China; xsgcf@163.com (C.G.); jiahongli@bjmu.edu.cn (H.J.); 2 Ningbo Institute of Marine Medicine, Peking University, Beijing 100191, China; kang.zhou@pkunimm.com; 3 School of Food and Pharmacy, Zhejiang Ocean University, Zhoushan 316022, China; wangbin@zjou.edu.cn

**Keywords:** *Clavularia viridis*, dolabellane diterpenoids, anti-inflammatory

## Abstract

A chemical investigation of the EtOAc fraction from soft coral *Clavularia viridis* resulted in the isolation of 12 undescribed dolabellane-type diterpenoids, namely clavirolides W–Z (**1**–**4**), clavularols A–H (**5**–**12**), and three known analogs (**13**–**15**). Their structures were characterized by an extensive analysis of spectroscopic data, including X-ray diffraction and ECD calculations for the assignment of absolute configurations. The structures of **2** and **4**–**6** are feathered as peroxyl-substituted derivatives, while compounds **7**–**12** possess additional oxidative cyclization, including epoxide or furan that are rare in the dolabellane family. All these compounds were evaluated for activities on cytotoxic and anti-inflammatory models. Compound **10** exhibited most potential against NO production in the BV2 cell induced by LPS with an IC_50_ value of 18.3 μM.

## 1. Introduction

**Figure 1:**
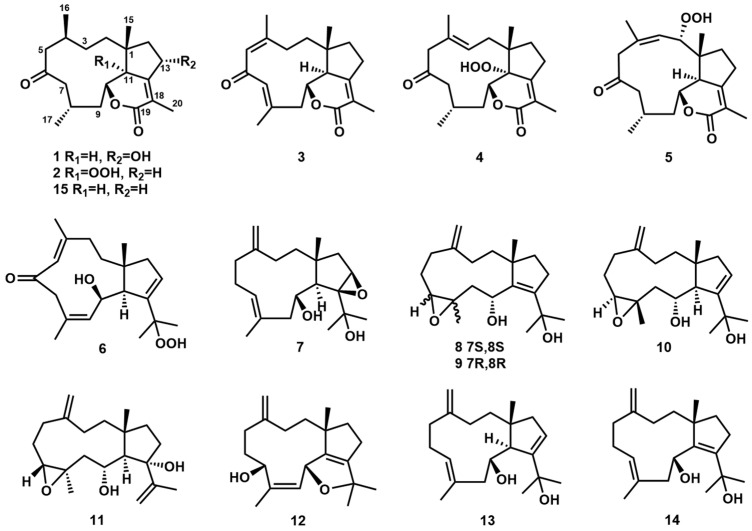
Structures of compounds **1**–**15**.

Diterpenoids derived from marine invertebrates significantly enrich the structural diversity and novelty of natural products [1,2]. Major classes include cembranes, briaranes, and eunicellanes, which originate from a common geranylgeranyl diphosphate (GGPP)-derived 14-membered carbocyclic precursor [3,4,5], while dolabellane-type diterpenoids, characterized by a distinctive 5,11-fused bicyclic core, arise from a fundamentally different cyclization mechanism in their biosynthesis. The subsequent oxidation on the backbone introduces additional stereochemical centers and ring systems (e.g., epoxides, peroxides, cyclic ethers) that strongly enhance structural novelty and complexity as well as molecular rigidity [6,7,8,9]; thus, it is supposed to exert a specific binding effect on target proteins to potentially improve pharmacological profiles [10]. Extensive biological evaluations revealed that marine diterpenoids generally display low cytotoxicity coupled with promising anti-inflammatory properties, often acting through mechanisms like the modulation of nitric oxide (NO) or pro-inflammatory cytokine production [11,12,13,14,15]. Based on our prior molecular networking (GNPS) analysis of the soft coral *Clavularia viridis*, which indicated a rich potential for novel dolabellanes [16], this study targeted higher polarity subfractions. Consequently, 12 new dolabellane diterpenoids were isolated and characterized. Compounds **2** and **4**–**6** incorporate peroxide functionalities, while compounds **7**–**12** contain epoxide or cyclic ether moieties (Figure 1). The inhibitory effects of these isolates on lipopolysaccharide (LPS)-induced NO production in mouse microglia BV-2 models were evaluated.

## 2. Results

### 2.1. Structural Elucidation

**Figure 2:**
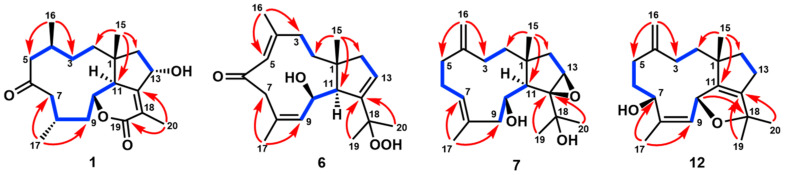
Key HMBC (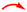) and ^1^H-^1^H COSY (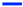) correlations of **1**, **6**, **7**, and **12**.

**Figure 3:**
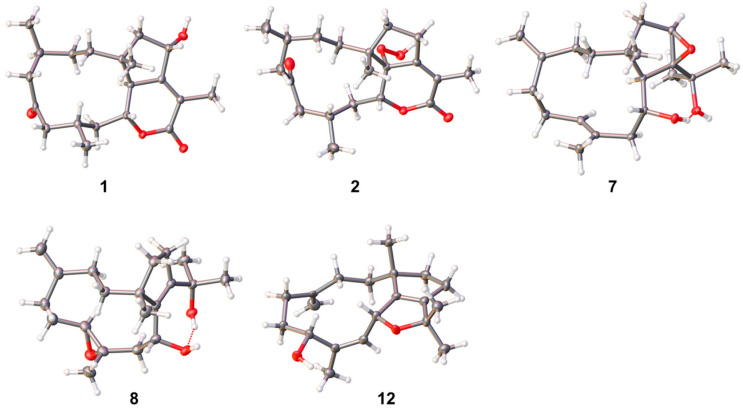
ORTEP views of the crystal structures of **1**, **2**, **7**, **8**, and **12**.

Clavirolide W (**1**) was obtained as a colorless crystal. Its molecular formula was assigned to C_20_H_30_O_4_ based on the HRESIMS data, requiring six degrees of unsaturation. The IR absorptions at 1702 cm^−1^ suggested the presence of ketone and lactone functionalities. The ^13^C NMR (APT) data presented 20 carbon resonances, including two olefinic carbons for a double bond and two carbonyl groups, which indicated a tricyclic skeleton of **1** due to the molecular unsaturation. These data assembled with ^1^H NMR (Table 1) unveiled **1** as a typical dolabellane with an *α*/*β*-unsaturated-*δ*-lactone ring and is extremely similar to the structure of clavirolide C (**15**) [17]. The only difference is the C-13 hydroxylation in **1**, which was confirmed from HMBC correlations from H-13 to C-1, C-11, C-12, and C-18, in addition to ^1^H-^1^H COSY correlations from H-13 to H_2_-14 (Figure 2). The NOESY interaction between H-13 and H_3_-15 suggested the same orientation of these protons (Figure S8). The other stereogenic centers were deduced to possess the same configurations as that of clavirolide C based on the biogenetic consideration. We cultured the single crystal of **1** and applied X-ray diffraction employing graphite-monochromated Cu Kα radiation to confirm its absolute configuration as 1*R*, 4*S*, 10*R*, 11*S*, 13*S* with a Flack parameter of 0.00(5) (Figure 3).

The molecular formula of clavirolide X (**2**) was determined to be C_20_H_30_O_5_ from HRESIMS data, containing one more oxygen atom than that of **1**. A comprehensive analysis of 1D and 2D NMR data revealed **2** as a peroxyl derivative of clavirolide C. The peroxyl group was located at C-11, certificated by its chemical shifts at *δ*_C_ 88.4 [18], which is significantly different from the hydroxyl counterpart (*δ*_C_ 79.1) in previous reported clavirolide M [16]. The structure of **2**, including its absolute configuration, was finally elucidated by X-ray diffraction data of a single crystal, as shown in Figure 3.

**Figure 4:**
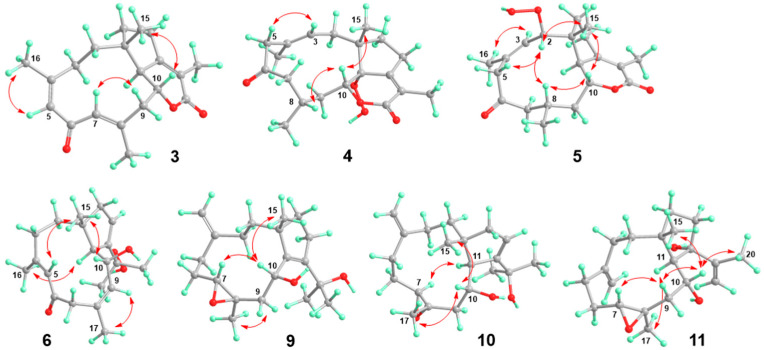
Key NOE correlations of **3**–**6** and **9**–**11**.

Clavirolide Y (**3**) was another analog of **15** based on 1D and 2D NMR data. The structural differences were two more double bonds existing in **3**. The olefinic singlets were observed at *δ*_H_ 5.86 and 6.36, which showed HMBC correlations with Me-16 (*δ*_C_ 25.5) and Me-17 (*δ*_C_ 18.2), indicating the location of *Δ*^4^ and *Δ*^7^ (Figure S23). The geometry was assigned as *Z* for *Δ*^4^ and *E* for *Δ*^7^ according to NOE interactions between H_3_-16 (*δ*_H_ 1.92) and H-5, as well as that between H-7 and H-9b (*δ*_H_ 2.57) (Figure 4 and Figure S24).

**Figure 5:**
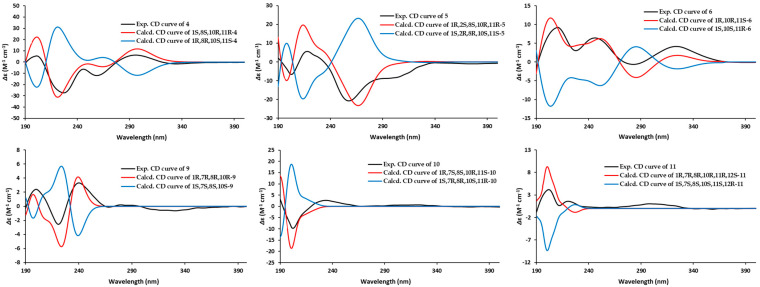
Experimental and calculated ECD data of **4**–**6** and **9**–**11**.

The structure of clavirolide Z (**4**) resembled that of clavirolide B by a comprehensive comparison of their NMR data, except for the quaternary carbon with a downfield chemical shift at *δ*_C_ 88.1 in **4**. This signal, together with the molecular formula deduced from HRESIMS, indicated a peroxyl group substitution, which was assigned to C-11 according to the key HMBC correlation with H_3_-15 (*δ*_H_ 1.09) (Figure S31). The relative configuration of **4** was identified by the NOE data (Figure 4 and Figure S32), of which the NOE correlation between H_3_-15 and H-10 (*δ*_H_ 4.46) suggested a *trans*fusion of rings A and B with the same orientation of H-10 and H_3_-15. In addition, the NOE interaction between H-10 and H-8 (*δ*_H_ 2.32) revealed the same orientation. The geometry of *Δ*^3^ was determined as *E* based on the NOE interaction between H-3 (*δ*_H_ 5.56) and H-5a (*δ*_H_ 3.47). The absolute configuration of compound **4** was established by comparing the experimental CD spectrum with the TDDFT-ECD calculated results (Figure 5). The configuration was finally unveiled as 1*S*, 8*S*, 10*R*, 11*R*.

The HRESIMS data of clavularol A (**5**) showed the molecular formula to be the same as that of **4**. Detailed NMR analysis suggested the peroxyl relocation from C-11 to C-2 in **5**. This was confirmed by a chemical shift in C-2 at *δ*_C_ 86.8 as well as a ^1^H-^1^H COSY correlation between H-2 (*δ*_H_ 5.18) and H-3 (*δ*_H_ 5.47) (Figure S38). In its NOE spectra, H-2 showed strong interactions with H-5a (*δ*_H_ 4.17), H-8 (*δ*_H_ 2.43), and H_3_-15 (*δ*_H_ 1.16), which interacted with H-10 (*δ*_H_ 4.29) as well, indicating the spatial proximity of these protons (Figure 4 and Figure S40). The NOE interaction between H-3 and H_3_-16 (*δ*_H_ 2.07) suggested a Z geometry of *Δ*^3^. The absolute configuration of **5** was elucidated as 1*R*, 2*S*, 8*S*, 10*R*, 11*R* according to the ECD calculation (Figure 5).

The ^13^C APT data (Table 2) of clavularol B (**6**) exhibited a total of 20 carbon resonances, including one conjugated ketone at *δ*_C_ 196.8, six olefinic carbons for three double bonds, and five methyl, four methylene, two methine, and two nonprotonated *sp*3 signals. In combination, the unsaturated degree deduced from the molecular formula as well as NMR features suggested **6** as a bicyclic dolabellane, which is structurally related to the previously reported clavirolide U [16]. A 4,5-ene-6-keto fragment also existed and was confirmed by HMBC correlations from H_3_-16 (*δ*_H_ 1.83) to C-4 (*δ*_C_ 153.2) and C-5 (*δ*_C_ 129.3), as well as from both H-5 (*δ*_H_ 6.36) and H_2_-7 (*δ*_H_ 3.70 and 2.63) to C-6 (*δ*_C_ 196.8) (Figure S47). The other two double bonds were assigned between C-8/C-9 and C-12/C-13, from ^1^H-^1^H COSY correlations between H-9 (*δ*_H_ 6.44) and H-10 (*δ*_H_ 4.88) and H-13 (*δ*_H_ 6.00) and H_2_-14 (*δ*_H_ 2.45 and 2.08) (Figure 2). In particular, a peroxyl rather than hydroxyl group was located at C-18, confirmed by its chemical shift at *δ*_C_ 81.0 and HMBC correlations from germinal methyl protons (H_3_-19 at *δ*_H_ 1.18 and H_3_-20 at *δ*_H_ 1.58) to C-18. The NOE data also supported the *trans*fusion of the two rings. The geometry of *Δ*^4^ was determined as *E* based on NOE interactions from H_3_-16 to H-11 (*δ*_H_ 2.43) and from H-5 to H_3_-15 (*δ*_H_ 1.48), which showed an interaction with H-10 as well, suggesting the same orientation of H_3_-15 and H-10 (Figure 4 and Figure S48). The geometry of the double bond between C-8 and C-9 was determined as *Z* by the NOE interaction from H-9 to H_3_-17 (*δ*_H_ 1.91). The absolute configuration of **6** was also unveiled by the ECD method as 1*R*, 10*R*, 11*S* (Figure 5).

The NMR data of clavularol C (**7**) (Table 2 and Table 3) also featured a dolabellane skeleton and was extremely similar to that of clavirolide R [16]. The only difference was a C-12/C-13 epoxide rather than C-12 hydroxylation in **7**, which was confirmed from the molecular formula and HMBC correlations from H-13 (*δ*_H_ 3.16) to C-14 (*δ*_C_ 36.7) and C-1 (*δ*_C_ 40.9), in addition to that from both H_3_-19 (*δ*_H_ 1.14) and H_3_-20 (*δ*_H_ 1.47) to C-12 (*δ*_C_ 73.3) (Figure 2). The stereochemistry of **7** was elucidated by the X-ray diffraction of the single crystal with a Flack parameter of −0.05(4), indicating 1*R*, 10*R*, 11*R*, 12*S*, 13*R* configurations (Figure 3).

Clavularol D (**8**) possessed the same molecular formula as that of **7**. Comprehensive NMR elucidation indicated the relocation of epoxide from C-12/C-13 to C-7/C-8, accompanied by double bond rearrangement from C-7/C-8 to C-11/C-12. In the NOESY spectra, H_3_-17 (*δ*_H_ 1.38) displayed interaction with H-10 (*δ*_H_ 3.99), which in turn interacts with H_3_-15 (*δ*_H_ 1.17), suggesting the same orientation of these protons. H-7 (*δ*_H_ 2.85) and H_3_-17 showed NOE interactions with H-9b (*δ*_H_ 2.33) and H-9a (*δ*_H_ 2.47), respectively, indicating a *trans*fusion of epoxide between C-7 and C-8 (Figure S64). The absolute configuration of **8** was also verified by the X-ray diffraction of the single crystal as 1*R*, 7*S*, 8*S*, 10*R* with a Flack parameter of −0.05(8) (Figure 3).

Clavularol E (**9**) has the same planar structure as that of **8**. A comparison of their NOESY spectrums turned out to be the configurational reverse for epoxide, certificated by the interaction between H-7 (*δ*_H_ 3.04) and H-10 (*δ*_H_ 4.15) together with that between H_3_-17 (*δ*_H_ 1.34) and H_2_-9 (*δ*_H_ 3.23 and 1.79) (Figure 4 and Figure S72). The absolute configuration of **9** was determined by the ECD method as 1*R*, 7*R*, 8*R*, 10*R* (Figure 5).

The structure of clavularol F (**10**) was almost same as that of **8** based on the 2D NMR and MS data, with the exception of the double bond migration from C-11/C-12 to C-12/C-13, confirmed by ^1^H-^1^H COSY correlations between H-13 (*δ*_H_ 5.71) and H_2_-14 (*δ*_H_ 2.23 and 2.10) (Figure S78). The relative configuration was established from NOE data (Figure 4 and Figure S80), of which H-10 (*δ*_H_ 3.91) showed interaction with H_3_-15 (*δ*_H_ 1.17) and H_3_-17 (*δ*_H_ 1.43), indicating that they were spatially close. Meanwhile, the NOE interaction between H-7 (*δ*_H_ 3.14) and H-11 (*δ*_H_ 3.00) suggested the same orientation. The ECD calculation was also applied for absolute configuration assignment. According to the comparison of the experimental CD curve with simulated ones, 1*R*, 7*S*, 8*S*, 10*R*, 11*S* was determined for corresponding stereogenic centers (Figure 5).

Clavularol G (**11**) has the same molecular formula and was determined as another isomer of **8**–**10**. The structural difference was verified as a relocation of a hydroxyl group from C-18 to C-12 with a double bond transfer to C-18/C-19, confirmed by key HMBC correlations from H_3_-20 (*δ*_H_ 2.03) to C-12 (*δ*_C_ 86.7), as well as C-18 (*δ*_C_ 151.4) and C-19 (*δ*_C_ 113.3) (Figure S87). The NOE interactions from H-9b (*δ*_H_ 1.61) to H-7 (*δ*_H_ 2.95) and H-10 (*δ*_H_ 4.03) and from H-10 to H_3_-15 (*δ*_H_ 1.05) and H_3_-20 indicated a *β* orientation of these protons. On the contrary, H_3_-17 (*δ*_H_ 1.51) displayed NOE interaction with H-11 (*δ*_H_ 2.53), suggesting an *α* orientation of these protons (Figure 4 and Figure S88). The absolute configuration of **11** was finally determined by the ECD method as 1*R*, 7*R*, 8*R*, 10*R*, 11*R*, 12*S* (Figure 5).

Clavularol H (**12**) was regarded as a dehydrated analog of **8** in terms of its molecular formula deduced from HRESIMS. The HMBC correlation from H-10 (*δ*_H_ 5.36) to C-18 (*δ*_C_ 82.6) confirmed the linkage of C-10 and C-18 through an ether bond (Figure 2). Moreover, the other structural differences were observed as C-7 hydroxylation and C-8/C-9 olefination, which were confirmed by HMBC correlations from H_3_-17 (*δ*_H_ 1.72) to C-7 (*δ*_C_ 67.7), as well as C-8 (*δ*_C_ 135.8) and C-9 (*δ*_C_ 132.7), combining with ^1^H-^1^H COSY correlation between H-9 (*δ*_H_ 5.17) and H-10. The NOE interactions from H-10 to H-7 (*δ*_H_ 4.88) and H_3_-15 (*δ*_H_ 1.05) indicated their relative relationship, and the absolute configuration was elucidated by X-ray diffraction of the single crystal as 1*R*, 7*R*, 10*R* with a Flack parameter of 0.02(6) (Figure 3).

### 2.2. Biological Activity Assessment

The above new compounds together with co-isolated clavularinlide E (**13**) [19], clavudiol A (**14**) [20], and clavirolide C (**15**) [17] were first tested for a cytotoxic effect in mouse microglia BV-2 cell lines using the Cell Counting Kit-8 (CCK-8) assay. All of them showed weak to no activity under a concentration of 50 μM. To evaluate the anti-inflammatory effect, all compounds were screened on an LPS-stimulated BV2 model to determine the inhibitory effects against NO production [21]. At a dose of 20 μM, compounds **3**, **10**, **12**, **13**, and **15** exhibited inhibitions between 42.7% to 68.9%. Further examination suggested their IC_50_ values as shown in Table 4, with L-NMMA as the positive control (IC_50_ = 17.8 μM). In association, activity data with structures reveals that the presence of an *α*/*β*-unsaturated lactone or furan ring significantly enhances bioactivity, as demonstrated by compounds **3**, **12**, and **15**. However, oxidation at any position between C-11 and C-13 severely reduces activity. The presence of a peroxyl group does not improve activity and may even diminish it. The stereogenic center at C-11 seems essential for these compounds to exert NO inhibitory effects. Desaturation between C-11 and C-12 reduces or abolishes the effect, which was evidenced by activity differences between compounds **8/9** and **10**, as well as that between compounds **13** and **14**. These preliminary SAR findings provide valuable insights for future structural optimization and mechanistic studies.

## 3. Materials and Methods

### 3.1. General Experimental Procedures

Optical rotations were measured on an Autopol III automatic polarimeter (Rudolph Research Co., Ltd. (Flanders, NJ, USA)). UV spectra were measured on a Cary 300 spectrometer (Agilent, Santa Clara, CA, USA). IR spectra were recorded on a Thermo Nicolet Nexus 470 FT-IR spectrometer (Waltham, MA, USA). ECD spectra were recorded on a JASCO-815 CD spectrometer (Tokyo, Japan). The ^1^H and ^13^C NMR spectra were recorded on a Bruker Advance 400 (400 MHz for ^1^H and 100 MHz for ^13^C, respectively). HRESIMS spectra were obtained from an Autospec Ultima-TFO spectrometer (Milford, MA, USA). Single crystal X-ray diffraction data were measured on an XtaLAB Synergy R HyPix diffractometer (Rigaku, Tokyo, Japan) equipped with a microfocus Cu Kα X-ray source (1.54184 Å) and a HyPix-6000C area detector (Rigaku, Tokyo, Japan). Silica gels (160–200 and 200–300 mesh, Qingdao Marine Chemistry Co., Ltd. (Qingdao, China)) and ODS (50 μm, YMC, Devens, MA, USA) were used for column chromatography. Sephadex LH-20 (18–110 μm) was purchased from Pharmacia (Peapack, NJ, USA). Semi-preparative HPLC chromatography was performed on an Alltech instrument ((426-HPLC pump), Nicholasville, KY, USA) equipped with an Alltech uvis-200 detector at 210 nm, and the Prevail C18 column ((semipreparative, 5 μm), Avantor, Radnor, PA, USA) was used for separation.

### 3.2. Animal Material

Soft coral *Clavularia viridis* was collected from the Yongxing island of Xisha, South China Sea, in May of 2017. The coral samples were frozen immediately after collection. The specimen coded XSA-115 was identified by Prof. Leen van Ofwegen (National Museum of National History Naturalis, Netherlands) and was deposited at the State Key Laboratory of Natural and Biomimetic Drugs, Peking University.

### 3.3. Extraction and Isolation

Frozen coral *C. viridis* (1.6 kg) was homogenized and extracted with 95% EtOH (2 L × 3). The concentrated EtOH extract (32.0 g) was desalted by dissolving in MeOH to obtain a residue, which was further partitioned between H_2_O and EtOAc. The EtOAc fraction was analyzed by LC-MS/MS, and annotation of the molecular cluster of the networking established by the GNPS platform implied the EtOAc fraction contained dolabellane-related terpenes. Thus, the EtOAc fraction (31.0 g) was subjected to column chromatography (12 × 90 cm) using silica gel (160–200 mesh, 1000 g) with a gradient of cyclohexane/acetone (from 100:0 to 0:100) to obtain five fractions (FA-FE). The ^1^H NMR detection revealed fractions FC to FD showing terpene features. FC (10.8 g) was separated by column chromatography (8.5 × 60 cm) using silica gel (300–400 mesh, 500 g) with a gradient of cyclohexane/acetone (9:2) to obtain five fractions (FCa-FCe). FCd (6.3 g) was separated by column chromatography (5.0 × 45 cm) using silica gel (300–400 mesh, 160 g) with a gradient of cyclohexane/acetone (9:2) to obtain five fractions (FCd1-FCd5). FCd3 (1.0 g) was chromatographed by semipreparetive HPLC with acetonitrile–H_2_O (7:3) to yield **14** (23.3 mg, retention time: 28.9 min), **15** (34.7 mg, retention time: 45.9 min), **6** (1.7 mg, retention time: 35.8 min), and **5** (0.8 mg, retention time: 19.1 min). FCd-5 (1.8 g) was chromatographed by Sephadex LH-20 gel with petroleum ether–EtOAc–MeOH (5:5:1) to obtain five fractions (FCd5.1-FCd5.5). FCd5.3 (345.4 mg) was chromatographed by semipreparetive HPLC with acetonitrile–H_2_O (8:2) to yield **11** (3.6 mg, retention time: 31.5 min), **7** (1.6 mg, retention time: 33.6 min), **9** (1.1 mg, retention time: 35.7 min), **4** (0.6 mg, retention time: 18.4 min), **1** (1.8 mg, retention time: 15.8 min), and **2** (5.1 mg, retention time: 21.7 min). FCe (2.2 g) was separated by an ODS column eluting with MeOH-H_2_O (8:2) as a mobile phase to yield **3** (1.9 mg, retention time: 33.8 min), **8** (25.2 mg, retention time: 36.6 min), and **10** (3.2 mg, retention time: 32.8 min). FD (14.4 g) was subjected to column chromatography (8.5 × 60 cm) using silica gel (160–200 mesh, 800 g) with a gradient of cyclohexane/acetone (from 100:0 to 0:100) to obtain four fractions (FDa-FDd). FDa (2.5 g) was purified on semipreparative HPLC with MeOH-H_2_O (8:2) as a mobile phase to obtain **13** (1.5 mg, retention time: 40.6 min) and **12** (6.5 mg, retention time: 42.3 min).

Clavirolide W (**1**): colorless crystal, mp 131.0–132.0 °C, ${\left[\alpha \right]}_{D}^{20}$ 10.6 (c 0.2, MeOH). IR (KBr) ν_max_ 3452, 2956, 1702 cm^−1^; ^1^H and ^13^C NMR data, see Table 1 and Table 3; HRESIMS *m*/*z* 335.2214 [M + H]^+^ (calcd. for C_20_H_31_O_4_, 335.2217).

Clavirolide X (**2**): colorless crystal, mp 103.5–104.5 °C, ${\left[\alpha \right]}_{D}^{20}$ −78.0 (c 0.4, MeOH). IR (KBr) ν_max_ 3333, 2955, 1699 cm^−1^; ^1^H and ^13^C NMR data, see Table 1 and Table 3; HRESIMS *m*/*z* 351.2162 [M + H]^+^ (calcd. for C_20_H_31_O_5_, 351.2166).

Clavirolide Y (**3**): colorless oil, ${\left[\alpha \right]}_{D}^{20}$ −69.0 (c 0.2, MeOH). IR (KBr) ν_max_ 2926, 1711, 1633 cm^−1^; ^1^H and ^13^C NMR data, see Table 1 and Table 3; HRESIMS *m*/*z* 315.1961 [M + H]^+^ (calcd. for C_20_H_27_O_3_, 315.1960).

Clavirolide Z (**4**): colorless oil, ${\left[\alpha \right]}_{D}^{20}$ −18.0 (c 0.1, MeOH). IR (KBr) ν_max_ 3396, 2928, 1702 cm^−1^; ^1^H and ^13^C NMR data, see Table 1 and Table 3; HRESIMS *m*/*z* 349.2006 [M + H]^+^ (calcd. for C_20_H_29_O_5_, 349.2009).

Clavularols A (**5**): colorless oil, ${\left[\alpha \right]}_{D}^{20}$ −23.0 (c 0.2, MeOH). IR (KBr) ν_max_ 3436, 2958, 1706 cm^−1^; ^1^H and ^13^C NMR data, see Table 1 and Table 3; HRESIMS *m*/*z* 349.2009 [M + H]^+^ (calcd. for C_20_H_29_O_5_, 349.2009).

Clavularols B (**6**): colorless oil, ${\left[\alpha \right]}_{D}^{20}$ 13.5 (c 0.1, MeOH). IR (KBr) ν_max_ 3334, 2931, 1697 cm^−1^; ^1^H and ^13^C NMR data, see Table 2 and Table 3; HRESIMS *m*/*z* 333.2074 [M − H]^−^ (calcd. for C_20_H_29_O_4_, 333.2066).

Clavularols C (**7**): colorless crystal, mp 144.2–145.0 °C, ${\left[\alpha \right]}_{D}^{20}$ 22.7 (c 0.1, MeOH). IR (KBr) ν_max_ 3280, 2927 cm^−1^; ^1^H and ^13^C NMR data, see Table 2 and Table 3; HRESIMS *m*/*z* 319.2283[M − H]^−^ (calcd. for C_20_H_31_O_3_, 319.2278).

Clavularols D (**8**): colorless crystal, mp 176.0–177.0 °C, ${\left[\alpha \right]}_{D}^{20}$ 10.2 (c 0.1, MeOH). IR (KBr) ν_max_ 3319, 2946, 1453 cm^−1^; ^1^H and ^13^C NMR data, see Table 2 and Table 3; HRESIMS *m*/*z* 365.2328[M + HCOO]^−^ (calcd. for C_21_H_33_O_5_, 365.2328).

Clavularols E (**9**): colorless oil, ${\left[\alpha \right]}_{D}^{20}$ 10.6 (c 0.2, MeOH). IR (KBr) ν_max_ 3364, 2931, 1703 cm^−1^; ^1^H and ^13^C NMR data, see Table 2 and Table 3; HRESIMS *m*/*z* 321.2424 [M + H]^+^ (calcd. for C_20_H_33_O_3_, 321.2424).

Clavularols F (**10**): colorless oil, ${\left[\alpha \right]}_{D}^{20}$ −12.0 (c 0.1, MeOH). IR (KBr) ν_max_ 3421, 2935, 1702 cm^−1^; ^1^H and ^13^C NMR data, see Table 2 and Table 3; HRESIMS *m*/*z* 321.2422 [M + H]^+^ (calcd. for C_20_H_33_O_3_, 321.2424).

Clavularols G (**11**): colorless oil, ${\left[\alpha \right]}_{D}^{20}$ 14.0 (c 0.1, MeOH). IR (KBr) ν_max_ 3390, 2971, 1452 cm^−1^; ^1^H and ^13^C NMR data, see Table 2 and Table 3; HRESIMS *m*/*z* 321.2417 [M + H]^+^ (calcd. for C_20_H_33_O_3_, 321.2424).

Clavularols H (**12**): colorless crystal, mp 125.1–125.9 °C, ${\left[\alpha \right]}_{D}^{20}$ −10.8 (c 0.1, MeOH). IR (KBr) ν_max_ 3386, 2928, 1451 cm^−1^; ^1^H and ^13^C NMR data, see Table 2 and Table 3; HRESIMS *m*/*z* 303.2315[M + H]^+^ (calcd. for C_20_H_31_O_2_, 303.2318).

### 3.4. X-Ray Crystallography Data

Single-crystal X-ray diffraction data were collected on a ROD, Synergy Custom DW system, HyPix diffractometer (Rigaku, Tokyo, Japan) equipped with a microfocus Cu Kα X-ray source (1.54184 Å) and a HyPix-6000HE area detector (Rigaku, Tokyo, Japan). Crystal was cooled to 100K using a cold nitrogen stream (Cobra by Oxford Cryosystems, Long Hanborough, UK). Date reduction, cell refinement, and experimental absorption correction were performed in CrysAlisPro. Using the Olex2 program package, the structure was solved with the SHELXS structure solution program using direct methods and refined against F2 with the SHELXL refinement package using full-matrix least squares minimization. A multi-scan method was used for the absorption correction. All non-hydrogen atoms were refined anisotropically. Hydrogen atoms were generated geometrically at the idealized position and constrained to ride on their parent.

The detailed crystallographic data of compounds **1**, **2**, **7**, **8**, and **12** are presented in the Supplementary Materials and deposited in the Cambridge Crystallographic Data Centre with accession numbers CCDC-2450602 for **1**, CCDC-2450605 for **2**, CCDC-2450601 for **7**, CCDC-2450604 for **8**, and CCDC-2450603 for **12**.

### 3.5. ECD Calculation

The TDDFT ECD calculations for compounds **4**–**6** and **9**–**11** were performed with the Gaussian 09 program. The conformational search was conducted by Spartan 14 software using the MMFF force field within an energy window of 5.0 kcal/mol. The conformers with the Boltzmann population above 5% were re-optimized at the B3LYP/631G(d) level in vacuo or M06-2X/6-311G(d,p). TDDFT ECD calculations were run at the CAM-B3LYP/TZVP or M06-2X/TZVP level with the SMD solvent model for methanol [22]. ECD spectra were generated using SpecDis Version 1.71 with 0.3 sigma/gamma (eV) after UV correction [23].

### 3.6. Cell Culture

The BV-2 mouse microglia cell line was purchased from the Cell Bank of Shanghai Institute of Biochemistry and Cell Biology, Chinese Academy of Sciences (Shanghai, China), and was cultured in Dulbecco’s modified Eagle medium (DMEM, Gibco Invitrogen Corp., Suzhou, China) supplemented with 10% fetal bovine serum (FBS), 100 units/mL penicillin, and 100 mg/mL streptomycin. The cells were placed at 37 °C in a humidified incubator containing 5% CO_2_.

### 3.7. CCK-8 Assay

Cells were pretreated with or without compounds for 2 h and then induced with 40 μg/mL LPS for another 48 h. Thereafter, cell proliferation was assessed using the cell counting kit-8 (CCK-8) assay (CobBio, Beijing, China) according to the manufacturer’s instructions. The absorbance was then measured at a wavelength of 450 nm with a microplate reader (Thermo Fisher Scientific, Marietta, OH, USA). The cell proliferation was expressed as a ratio of control.

### 3.8. Inhibitory Activity Against NO Production

The inhibitory activity of the isolated compounds toward NO production was determined using the Griess reagent system (Beyotime). Cells were cultured in each well of 96-well plates at a density of 5 × 10^4^ cells/well with 100 mL of DMEM for 24 h. Test samples dissolved in DMSO and diluted with DMEM were added, as well as 2 mg/mL LPS as a stimulus. The final drug concentration was 50 μM with 1% DMSO. Wells treated with only LPS served as model controls, and wells treated with neither LPS nor test samples served as blank controls (all contained 1% DMSO). After 24 h incubation, half of the medium (50 mL) in each well was harvested.

### 3.9. Statistical Analysis

The data were presented as mean ± standard deviation (mean ± SD). Data from more than two groups were analyzed with a one-way ANOVA with the Bonferroni post hoc test, and only results with *p* < 0.05 were considered statistically significant. GraphPad Prism 8 software (version 8) was used for statistical analysis.

## 4. Conclusions

In conclusion, we unambiguously identified 12 new dolabellane diterpenoids, including their absolute configurations. Compounds **3**, **10**, **12**, **13**, and **15** exerted potential inhibitory effects against NO production induced by LPS on BV2 cell lines. The preliminary SAR analysis indicated that oxidative modifications on the five-membered ring drastically reduce biological activity, while the stereogenic center at C-11 is crucial. Correspondingly, the eleven-membered macrocycle exhibits relative conformational flexibility, showing less significant activities despite the different substitutions on the macrocycle. These results demonstrate that the high structural rigidity within the eastern moiety of the dolabellane core plays a vital role in enabling bioactivity, while the specific mechanistic details remain to be elucidated. In contrast to terrestrial plants, it is worth investigating the ecological significance of corals producing such high oxygenated metabolites under oxygen-depleted marine conditions. This understanding could offer valuable clues for uncovering the unique bioactivities associated with these compounds.

## Figures and Tables

**Table 1:** ^1^H NMR data of **1**–**5** (CDCl_3_, *δ* (ppm), *J* (Hz), 400 MHz).

No.	1	2	3	4	5
2	1.46, m	1.47, m 1.32, m	1.80, m 1.75, m	2.70, dd (15.2, 11.5) 1.87, d (15.2)	5.18, d (11.0)
3	1.46, m 1.31, m	1.33, m 1.00, m	2.75, m 1.82, m	5.56, d (11.5)	5.47, d (11.0)
4	2.52, m	2.20, m			
5	2.72, dd (15.0, 12.0) 2.09, m	2.37, dd (14.5, 10.8) 2.26, dd (14.5, 2.7)	5.86, br s	3.47, d (10.8) 2.86, d (10.8)	4.17, d (12.2) 2.62, d (12.2)
7	2.54, m 2.10, m	2.35, m	6.36, br s	2.59, m 2.21, dd (14.1, 11.0)	2.71, dd (13.8, 4.2) 2.14, d (13.8)
8	2.12, m	2.10, m		2.32, m	2.43, m
9	1.94, m 1.70, m	2.33, m 1.69, m	2.86, dd (14.5, 4.6) 2.57, dd (14.5, 4.6)	2.45, ddd (15.6, 3.6, 2.4) 1.49, ddd (15.6, 10.2, 5.2)	1.72, m 1.61, m
10	4.18, dd (10.8, 9.3)	4.49, t (4.4)	4.56, dt (11.2, 4.6)	4.46, dd (5.2, 2.4)	4.29, dd (11.0, 9.5)
11	2.78, d (10.8)		2.71, d (11.2)		2.65, d (11.0)
13	4.82, m	2.56, m	2.52, m	2.58, m	2.48, m
14	2.01, m; 1.69, m	2.06, m 1.52, m	1.92, m 1.43, m	2.14, m 1.60, m	1.81, m 1.73, m
15	0.87, s	0.91, s	0.95, s	1.09, s	1.16, s
16	0.99, d (6.8)	1.01, d (6.8)	1.92, d (1.2)	1.77, br s	2.07, s
17	1.17, d (6.0)	1.28, d (6.5)	2.07, s	1.17, d (6.6)	1.12, d (6.6)
19	1.99, s	1.92, brs	1.87, brs	1.95, br s	1.84, s

**Table 2:** ^13^C NMR data of compounds **1**–**12** (CDCl_3_, *δ* (ppm), 100 MHz).

No.	1	2	3	4	5	6	7	8	9	10	11	12
1	41.9	49.6	45.1	51.3	48.1	45.1	40.9	52.4	53.1	45.8	43.3	45.3
2	32.3	30.9	36.2	33.5	86.8	40.1	38.0	37.8	37.0	38.5	38.9	34.3
3	27.0	32.0	29.2	126.5	120.9	36.7	30.7	31.8	31.4	31.8	31.4	34.8
4	29.1	31.3	153.2	130.1	140.7	153.2	151.8	150.3	150.4	148.8	149.1	149.5
5	46.2	50.9	128.9	54.0	43.1	129.3	37.3	33.5	31.3	32.0	32.1	30.9
6	211.8	212.5	196.4	207.2	206.4	196.8	29.7	30.9	27.8	26.4	26.8	32.1
7	53.5	52.3	133.1	53.4	56.1	46.5	128.7	65.3	64.1	62.9	68.1	67.7
8	26.9	31.3	142.9	31.3	26.2	129.3	131.7	60.2	60.5	59.4	60.2	135.8
9	43.2	36.8	45.1	37.2	43.0	132.5	47.0	51.6	47.6	48.2	44.4	132.7
10	78.2	80.8	79.9	81.1	77.4	69.5	68.3	64.1	65.9	67.1	69.4	75.5
11	50.8	88.4	46.2	88.1	52.7	56.5	46.6	143.0	141.7	55.1	57.1	150.2
12	159.0	153.0	161.3	153.4	159.3	141.0	73.3	144.7	145.8	146.7	86.7	149.1
13	69.6	26.1	27.5	25.3	26.9	133.7	59.9	33.6	33.4	127.7	39.3	23.7
14	47.9	37.0	35.1	39.2	31.1	50.9	36.7	33.8	32.4	44.9	36.7	47.1
15	23.9	23.1	23.1	23.2	20.3	22.2	28.6	28.7	28.1	24.9	25.4	21.9
16	19.9	21.5	25.5	16.7	25.1	17.7	111.9	110.7	111.5	111.3	112.0	109.9
17	21.7	22.9	18.2	20.9	20.1	26.2	17.5	17.3	19.8	17.6	19.6	17.4
18	123.6	126.3	120.4	126.7	120.7	81.0	71.1	73.0	73.2	71.1	151.4	82.6
19	11.9	12.9	12.8	13.1	12.7	24.6	26.3	31.6	32.0	31.6	113.3	28.0
20	166.6	165.2	165.7	164.3	165.7	25.2	28.6	30.1	29.2	31.2	22.0	28.1

**Table 3:** ^1^H NMR data of **6**–**12** (CDCl_3_, *δ* (ppm), *J* (Hz), 400 MHz).

No.	6	7	8	9	10	11	12
2	1.96, dd (14.0, 3.6) 1.37, dt (14.0, 4.3)	1.33, m 1.09, dt (12.0, 2.0)	1.49, m	1.79, m	1.61, m	1.75, ddd (14.5, 10.0, 5.8) 1.38, ddd (14.5, 10.0, 6.4)	1.54, m 1.41, m
3	2.54, td (13.0, 3.6) 2.12, m	2.13, m 1.78, t (10.8)	2.19, m 1.78, m	2.19, m 1.88, m	2.28, m 1.97, m	2.25, m	2.10, m 1.85, m
5	6.36, s	2.41, m 2.11, m	2.35, m 2.04, m	2.36, m 2.04, m	2.29, m	2.48, m 2.29, m	1.99, m
6		2.33, m 2.13, m	2.10, m 1.38, m	2.21, m 1.19 m	2.28, m 1.48, m	2.07, m 1.49, m	1.89, m
7	3.70, d (10.6) 2.63, d (10.6)	5.44, dd (9.2, 5.6)	2.85, dd (10.2, 2.9)	3.04, d (10.5)	3.14, t dd (9.4, 2.3)	2.95, dd (10.0, 1.1)	4.88, m
9	6.44, d (9.8)	2.77, dd (12.6, 11.6) 2.26, dd (12.6, 1.9)	2.47, d (13.7) 2.33, m	3.23, t (12.7) 1.79, m	2.22, m 2.02, m	2.44, dd (14.8, 7.0) 1.61, dd (14.8, 3.9)	5.17, d (9.6)
10	4.88, d (9.8)	3.89, dt (11.6, 1.9)	3.99, d (9.9)	4.15, dd (11.0, 4.7)	3.91, dd (11.8, 1.5)	4.03, ddd (7.4, 7.0, 3.9)	5.36, dd (9.6, 2.9)
11	2.43, s	2.77, d (1.9)			3.00, d (1.5)	2.53, d (7.4)	
13	6.00, m	3.16, br s	2.34, m 2.22, m	2.29, m	5.71, dd (4.4, 2.4)	2.20, m 1.67, m	2.31, m 2.03, m
14	2.45, m 2.08, m	1.78, d (15.4) 1.71, d (15.4)	1.78, m 1.56, m	2.43, m 1.88, m	2.23, m 2.10, d (16.5)	1.95, dt (12.8, 7.5) 1.47, m	2.03, m
15	1.48, s	1.10, s	1.17, s	1.10, s	1.17, s	1.05, s	1.05, s
16	1.83, s	4.80, brs 4.69, brs	4.80, brs 4.78, brs	4.71, brs 4.69, brs	4.90, brs 4.85, brs	4.90, brs 4.81, brs	4.79, brs 4.74, brs
17	1.91, d (1.4)	1.73, s	1.38, s	1.34, s	1.43, s	1.51, s	1.72, s
19	1.18, s	1.14, s	1.38, s	1.39, s	1.34, s	5.32, brs 5.13, brs	1.24, s
20	1.58, s	1.47, s	1.40, s	1.47, s	1.55, s	2.03, s	1.42,rs

**Table 4:** NO inhibitory activity of compounds **1**–**15**.

Compound	NO Inhibitory Activity		CC_50_ (μM)
	% Inhibition (20 μM)	IC_50_ (μM)	
**1**	15.1 ± 2.4		>50
**2**	36.4 ± 1.7		>50
**3**	42.7 ± 3.5	27.0 ± 0.6	>50
**4**	29.0 ± 2.8		>50
**5**	26.8 ± 3.6		>50
**6**	14.6 ± 1.2		>50
**7**	24.0 ± 3.8		>50
**8**	35.9 ± 4.2		>50
**9**	12.6 ± 2.6		>50
**10**	68.9 ± 3.6	18.3 ± 0.6	>50
**11**	18.4 ± 2.8		>50
**12**	48.8 ± 4.2	20.3 ± 0.3	>50
**13**	45.6 ± 3.5	20.3 ± 0.3	>50
**14**	25.4 ± 2.4		>50
**15**	48.3 ± 3.6	23.4 ± 0.4	>50
L-NMMA ^a^	76.7 ± 4.2	17.8 ± 0.6	-

Results are expressed as average SD (*n* = 6). ^a^ L-NMMA: L-NG-monomethyl arginine, an inhibitor of nitric oxide synthase (NOS).

## Data Availability

Data is contained within the article and Supplementary Materials.

## Supplementary Materials

The following supporting information can be downloaded at: https://www.mdpi.com/article/10.3390/md23080312/s1, Figures S1–S96: HRESIMS, IR, 1D, and 2D NMR spectra of 1–12; X-ray crystallography data of compounds **1**, **2**, **7**, **8**, and **12**.
