# Supplementary material for: Dolabellane Diterpenoids from Soft Coral Clavularia viridis with Anti-Inflammatory Activities
Source: Mar Drugs. 2025 Jul 30;23(8):312. doi: 10.3390/md23080312 (PMC12387469; doi:10.3390/md23080312)
Supplement: Supplementary file 1 [file marinedrugs-23-00312-s001.zip › marinedrugs-3788585-supplementary.pdf]

Dolabellane diterpenoids from soft coral *Clavularia viridis* with anti-inflammatory activities

Chufan Gu [1], Hongli Jia [1], Kang Zhou [2], Bin Wang [3], Wenhan Lin [1,2,]* and Wei Cheng [1,]*

1 State Key Laboratory of Natural and Biomimetic Drugs, Peking University, Beijing, 100191, P.R. China; xsgcf@163.com (C. G.); jiahongli@bjmu.edu.cn (H. J.)

2 Ningbo Institute of Marine Medicine, Peking University, Beijing, 100191, P.R. China; kang.zhou@pkunimm.com (K. Z.)

3 Zhejiang Ocean University, Zhoushan, 316022, P.R. China; wangbin@zjou.edu.cn (B. W.)

* Correspondence: whlin@bjmu.edu.cn (W. L.); chengwei@bjmu.edu.cn (W. C.)

Supporting information

Table of contents

Figure S1. HRESIMS spectrum of compound **1**. 1

Figure S2. IR spectrum of compound **1**. 1

Figure S3. ^{1}H NMR spectrum of compound **1** in $CDCl_3$. 2

Figure S4. ^{13}C NMR(APT) spectrum of compound **1** in $CDCl_3$. 2

Figure S5. HSQC spectrum of compound **1** in $CDCl_3$. 3

Figure S6. ^{1}H-^{1}H-COSY spectrum of compound **1** in $CDCl_3$. 3

Figure S7. HMBC spectrum of compound **1** in $CDCl_3$. 4

Figure S8. NOESY spectrum of compound **1** in $CDCl_3$. 4

Figure S9. HRESIMS spectrum of compound **2**. 5

Figure S10. IR spectrum of compound **2**. 5

Figure S11. ^{1}H NMR spectrum of compound **2** in $CDCl_3$. 6

Figure S12. ^{13}C NMR(APT) spectrum of compound **2** in $CDCl_3$. 6

Figure S13. HSQC spectrum of compound **2** in $CDCl_3$. 7

Figure S14. ^{1}H-^{1}H-COSY spectrum of compound **2** in $CDCl_3$. 7

Figure S15. HMBC spectrum of compound **2** in $CDCl_3$. 8

Figure S16. NOESY spectrum of compound **2** in $CDCl_3$. 8

Figure S17. HRESIMS spectrum of compound **3**. 9

Figure S18. IR spectrum of compound **3**. 9

Figure S19. ^{1}H NMR spectrum of compound **3** in $CDCl_3$. 10

Figure S20. ^{13}C NMR(APT) spectrum of compound **3** in $CDCl_3$. 10

Figure S21 HSQC spectrum of compound **3** in $CDCl_3$. 11

Figure S22. ^{1}H-^{1}H-COSY spectrum of compound **3** in $CDCl_3$. 11

Figure S23. HMBC spectrum of compound **3** in $CDCl_3$. 12

Figure S24. NOESY spectrum of compound **3** in $CDCl_3$........................12

Figure S25. HRESIMS spectrum of compound **4**. 13

Figure S26. IR spectrum of compound **4**..13

Figure S27. ^{1}H NMR spectrum of compound **4** in $CDCl_3$.......................14

Figure S28. ^{13}C NMR(APT) spectrum of compound **4** in $CDCl_3$. 14

Figure S29 HSQC spectrum of compound **4** in $CDCl_3$. 15

Figure S30. ^{1}H-^{1}H-COSY spectrum of compound **4** in $CDCl_3$. 15

Figure S31. HMBC spectrum of compound **4** in $CDCl_3$. 16

Figure S32. NOESY spectrum of compound **4** in $CDCl_3$........................16

Figure S33. HRESIMS spectrum of compound **5**. 17

Figure S34. IR spectrum of compound **5**..17

Figure S35. ^{1}H NMR spectrum of compound **5** in $CDCl_3$.......................18

Figure S36. ^{13}C NMR(APT) spectrum of compound **5** in $CDCl_3$. 18

Figure S37 HSQC spectrum of compound **5** in $CDCl_3$. 19

Figure S38. ^{1}H-^{1}H-COSY spectrum of compound **5** in $CDCl_3$. 19

Figure S39. HMBC spectrum of compound **5** in $CDCl_3$. 20

Figure S40. NOESY spectrum of compound **5** in $CDCl_3$........................20

Figure S41. HRESIMS spectrum of compound **6**. 21

Figure S42. IR spectrum of compound **6**..21

Figure S43. ^{1}H NMR spectrum of compound **6** in $CDCl_3$.......................22

Figure S44. ^{13}C NMR(APT) spectrum of compound **6** in $CDCl_3$. 22

Figure S45 HSQC spectrum of compound **6** in $CDCl_3$. 23

Figure S46. ^{1}H-^{1}H COSY spectrum of compound **6** in $CDCl_3$...............23

Figure S47. HMBC spectrum of compound **6** in $CDCl_3$. 24

Figure S48. NOESY spectrum of compound **6** in $CDCl_3$........................24

Figure S49 HRESIMS spectrum of compound **7** 25

Figure S50 IR spectrum of compound **7**..25

Figure S51 ^{1}H NMR spectrum of compound **7** in $CDCl_3$........................26

Figure S52 ^{13}C NMR(APT) spectrum of compound **7** in $CDCl_3$ 26

Figure S53 HSQC spectrum of compound **7** in $CDCl_3$ 27

Figure S54 ^{1}H-^{1}H COSY spectrum of compound **7** in $CDCl_3$................27

Figure S55 HMBC spectrum of compound **7** in $CDCl_3$ 28

Figure S56 NOESY spectrum of compound **7** in $CDCl_3$........................28

Figure S57 HRESIMS spectrum of compound **8** 29

Figure S58 IR spectrum of compound **8**..29

Figure S59 ^{1}H NMR spectrum of compound **8** in $CDCl_3$........................30

Figure S60 ^{13}C NMR spectrum of compound **8** in $CDCl_3$.......................30

Figure S61 HSQC spectrum of compound **8** in $CDCl_3$ 31

Figure S62 ^{1}H-^{1}H COSY spectrum of compound **8** in $CDCl_3$................31

Figure S63 HMBC spectrum of compound **8** in $CDCl_3$ 32

Figure S64 NOESY spectrum of compound **8** in $CDCl_3$........................32

Figure S65 HRESIMS spectrum of compound **9** 33

Figure S66 IR spectrum of compound **9** 33

Figure S67 ^{1}H NMR spectrum of compound **9** in $CDCl_3$ 34

Figure S68 ^{13}C NMR (APT) spectrum of compound **9** in $CDCl_3$ 34

Figure S69 HSQC spectrum of compound **9** in $CDCl_3$ 35

Figure S70 ^{1}H-^{1}H COSY spectrum of compound **9** in $CDCl_3$ 35

Figure S71 HMBC spectrum of compound **9** in $CDCl_3$ 36

Figure S72 NOESY spectrum of compound **9** in $CDCl_3$ 36

Figure S73 HRESIMS spectrum of compound **10** 37

Figure S74 IR spectrum of compound **10** 37

Figure S75 ^{1}H NMR spectrum of compound **10** in $CDCl_3$ 38

Figure S76 ^{13}C NMR (APT) spectrum of compound **10** in $CDCl_3$ 38

Figure S77 HSQC spectrum of compound **10** in $CDCl_3$ 39

Figure S78 ^{1}H-^{1}H COSY spectrum of compound **10** in $CDCl_3$ 39

Figure S79 HMBC spectrum of compound **10** in $CDCl_3$ 40

Figure S80 NOESY spectrum of compound **10** in $CDCl_3$ 40

Figure S81 HRESIMS spectrum of compound **11** 41

Figure S82 IR spectrum of compound **11** 41

Figure S83 ^{1}H NMR spectrum of compound **11** in $CDCl_3$ 42

Figure S84 ^{13}C NMR (APT) spectrum of compound **11** in $CDCl_3$ 42

Figure S85 HSQC spectrum of compound **11** in $CDCl_3$ 43

Figure S86 ^{1}H-^{1}H COSY spectrum of compound **11** in $CDCl_3$ 43

Figure S87 HMBC spectrum of compound **11** in $CDCl_3$ 44

Figure S88 NOESY spectrum of compound **11** in $CDCl_3$ 44

Figure S89 HRESIMS spectrum of compound **12** 45

Figure S90 IR spectrum of compound **12** 45

Figure S91 ^1H NMR spectrum of compound **12** in $CDCl_3$......................46

Figure S92 ^{13}C NMR (APT) spectrum of compound **12** in $CDCl_3$ 46

Figure S93 HSQC spectrum of compound **12** in $CDCl_3$ 47

Figure S94 ^1H-^1H COSY spectrum of compound **12** in $CDCl_3$...............47

Figure S95 HMBC spectrum of compound **12** in $CDCl_3$ 48

Figure S96 NOESY spectrum of compound **12** in $CDCl_3$.......................48

X-ray crystallography data of compounds 1, 2, 7, 8 and 12 49

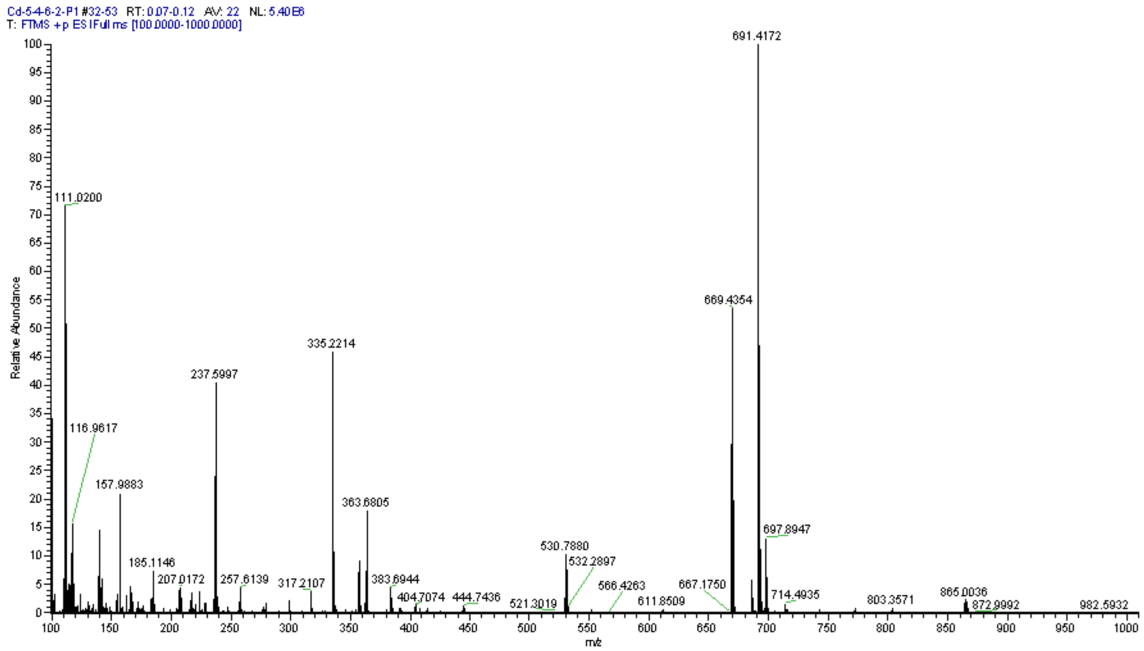

Mass	Calc. Mass	mDa	PPM	DBE	i-FIT	Norm	Conf(%)	Formula
335.2214	335.2217	0.0	-0.853	5.5	259.8	n/a	n/a	$C_{20}H_{31}O_4$

Figure S1. HRESIMS spectrum of compound **1**.

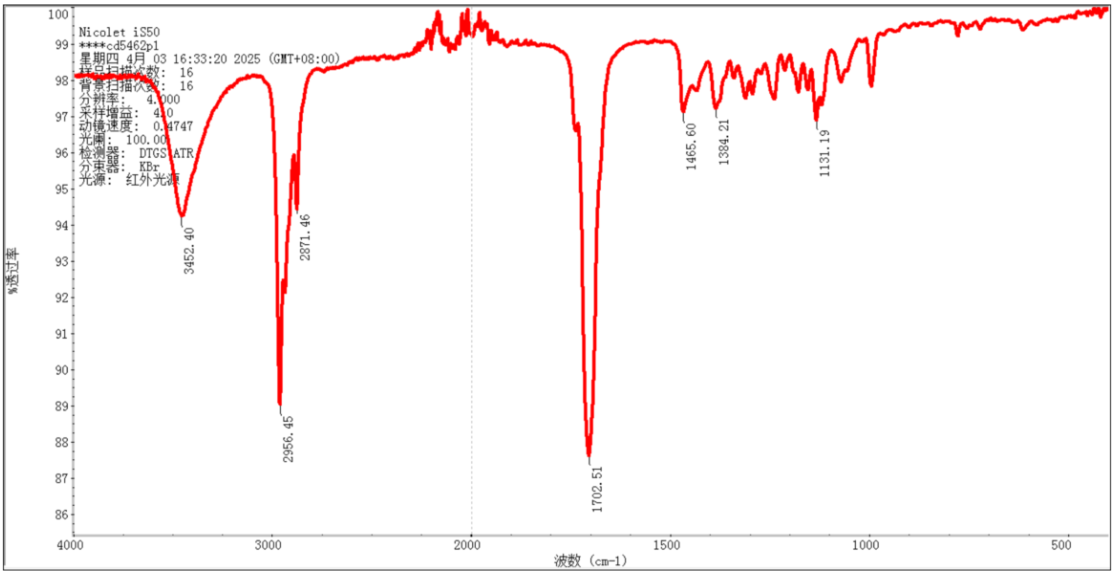

Figure S2. IR spectrum of compound **1**.

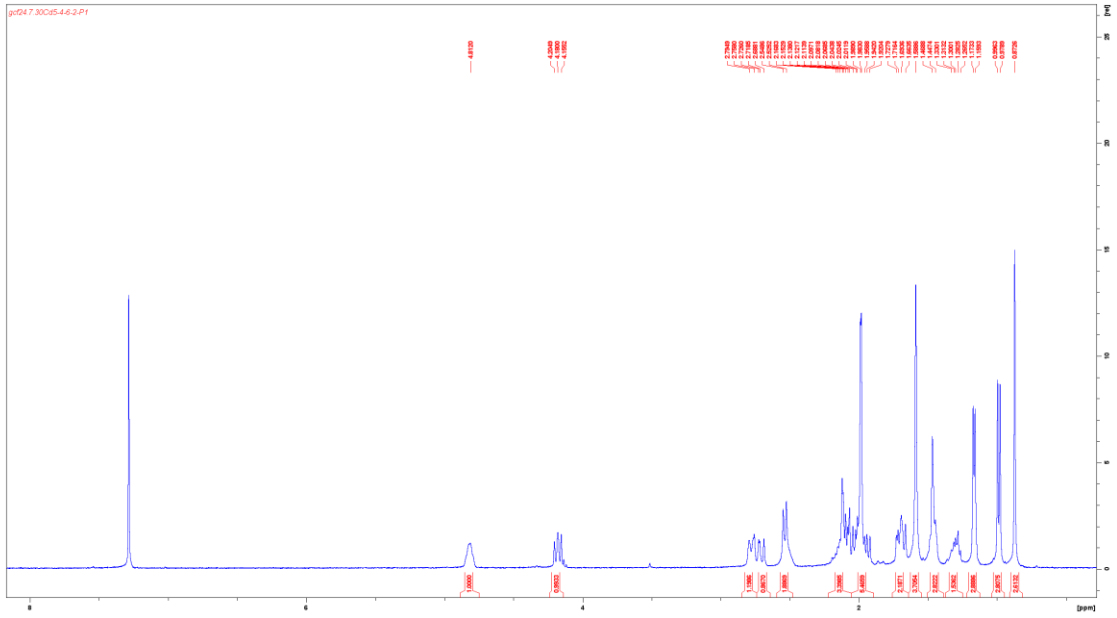

Figure S3. ^{1}H NMR spectrum of compound **1** in $CDCl_3$.

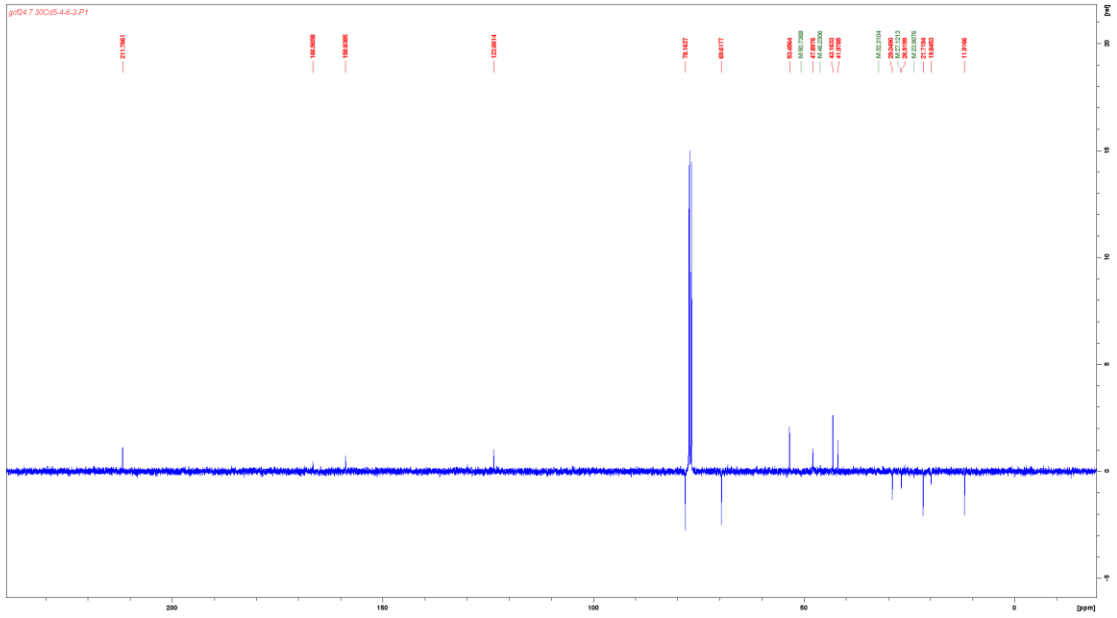

Figure S4. ^{13}C NMR(APT) spectrum of compound **1** in $CDCl_3$.

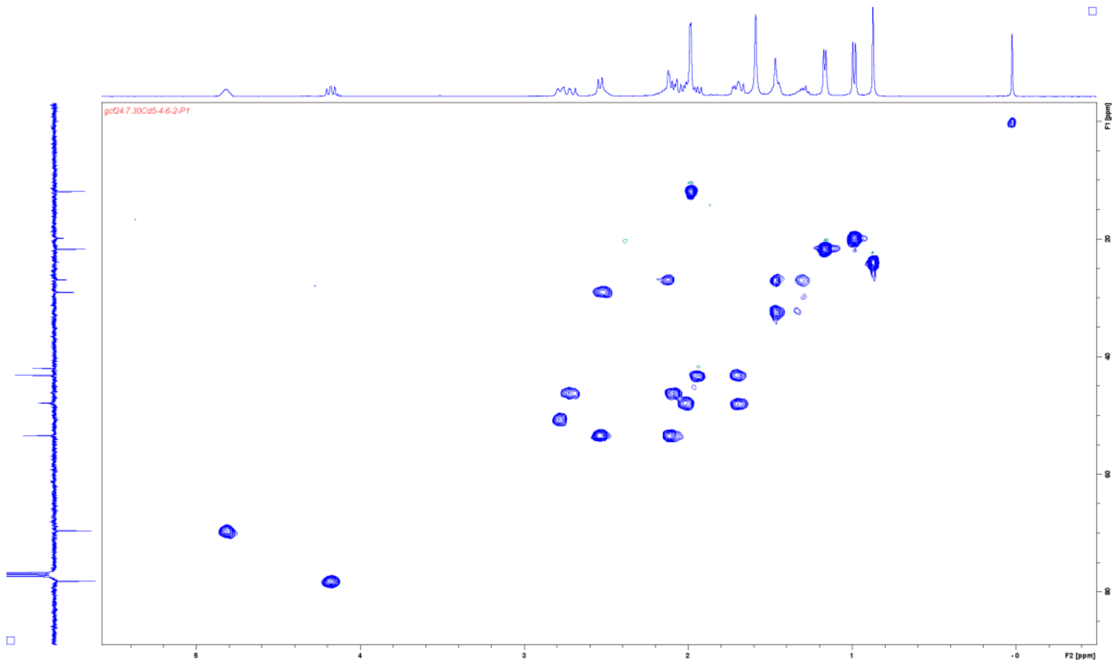

Figure S5. HSQC spectrum of compound **1** in $CDCl_3$.

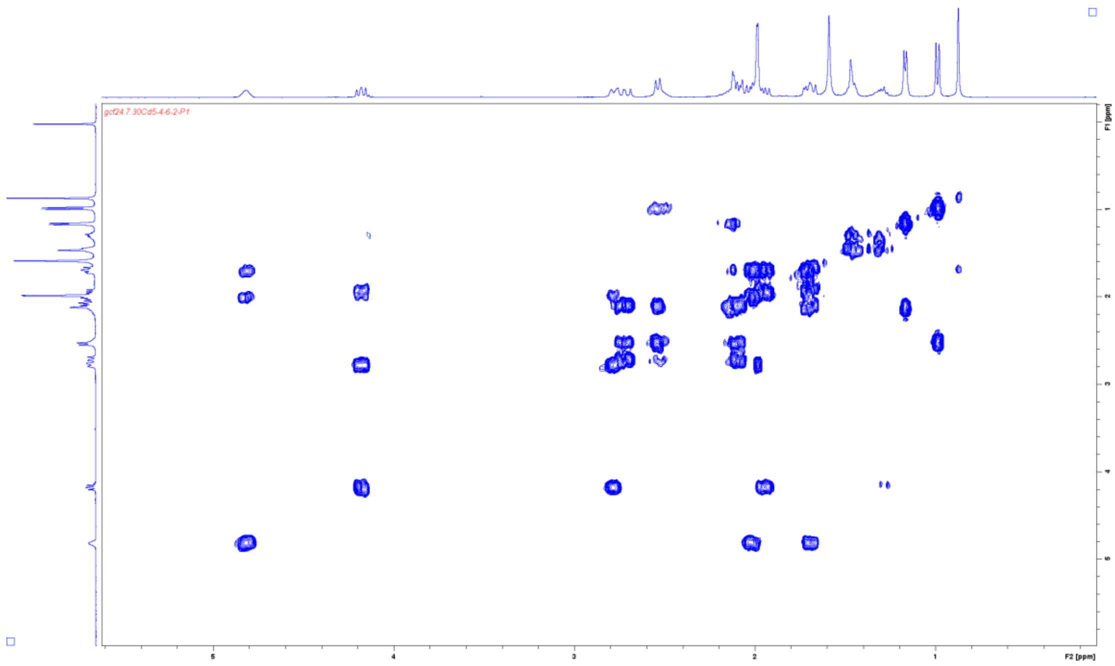

Figure S6. ^1H-^1H-COSY spectrum of compound **1** in $CDCl_3$.

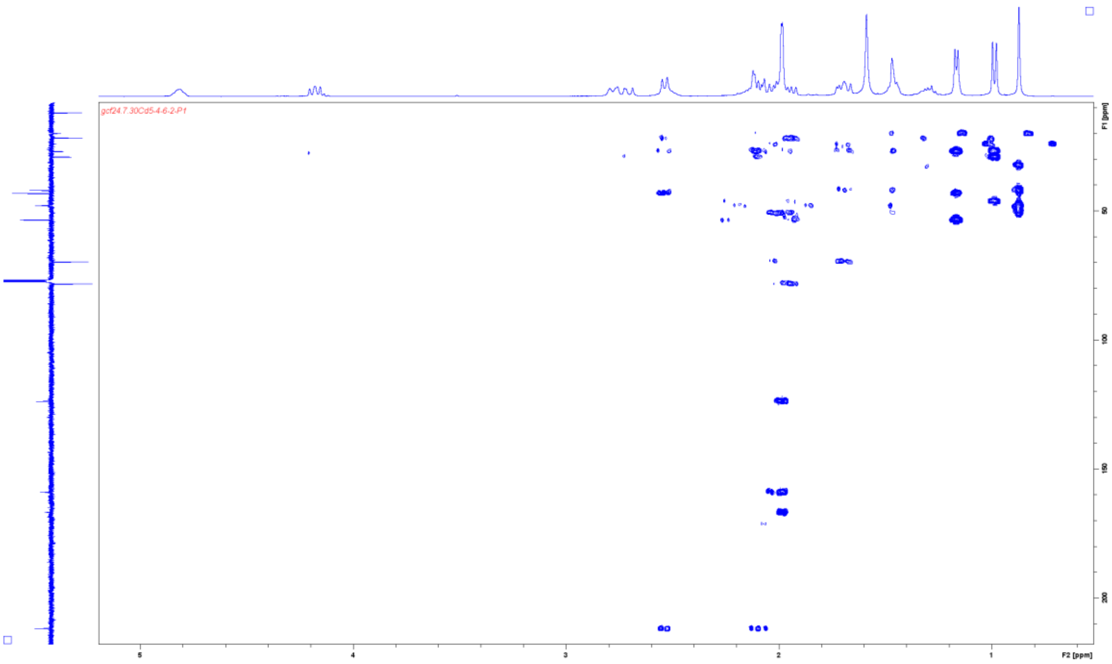

Figure S7. HMBC spectrum of compound **1** in $CDCl_3$.

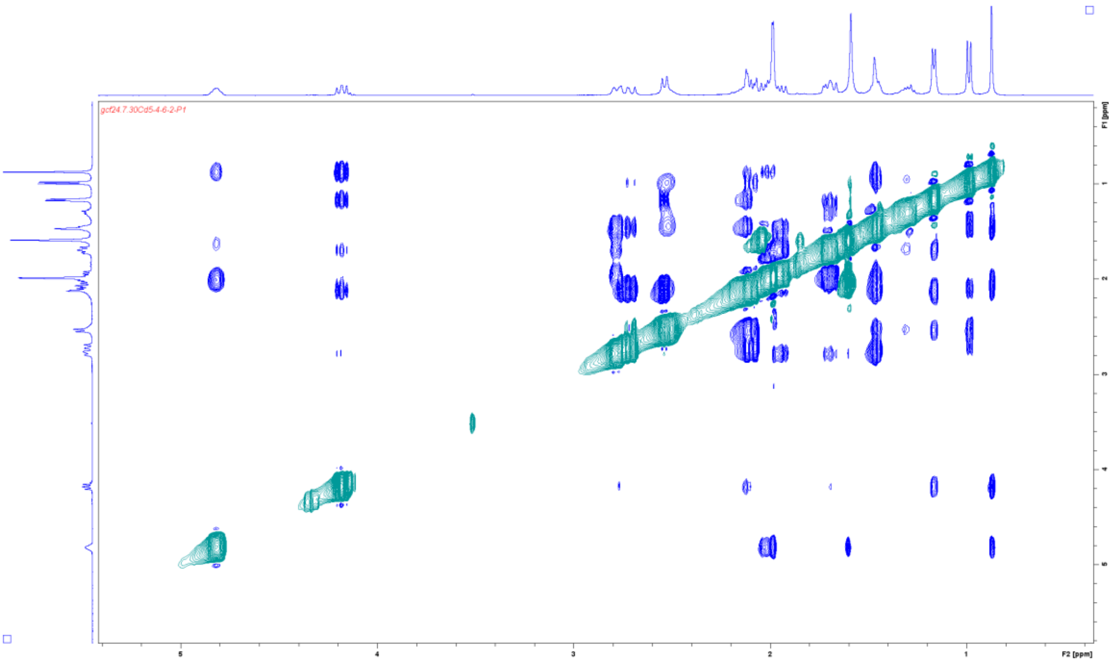

Figure S8. NOESY spectrum of compound **1** in $CDCl_3$.

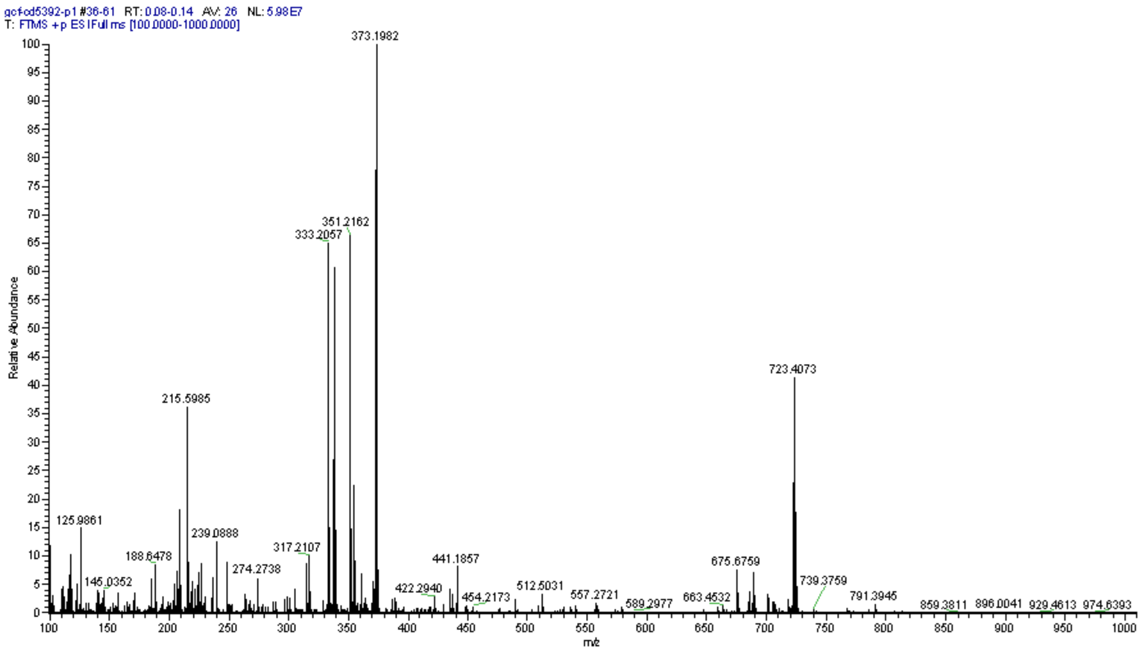

Mass	Calc. Mass	mDa	PPM	DBE	i-FIT	Norm	Conf(%)	Formula
351.2162	351.2166	0.0	-1.14	5.5	105.6	n/a	n/a	$C_{20}H_{31}O_5$

Figure S9. HRESIMS spectrum of compound **2**.

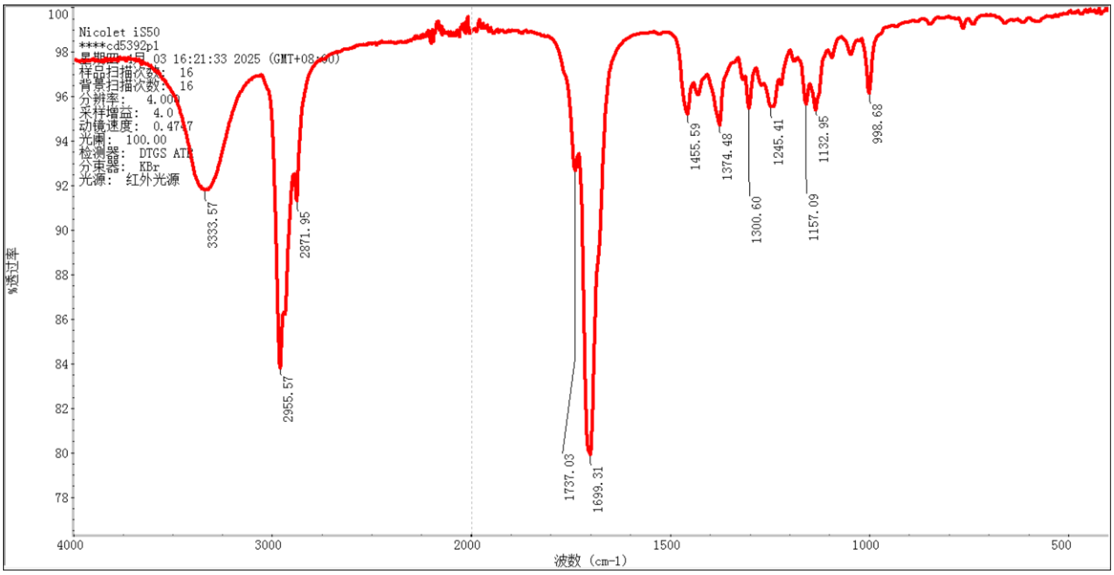

Figure S10. IR spectrum of compound **2**.

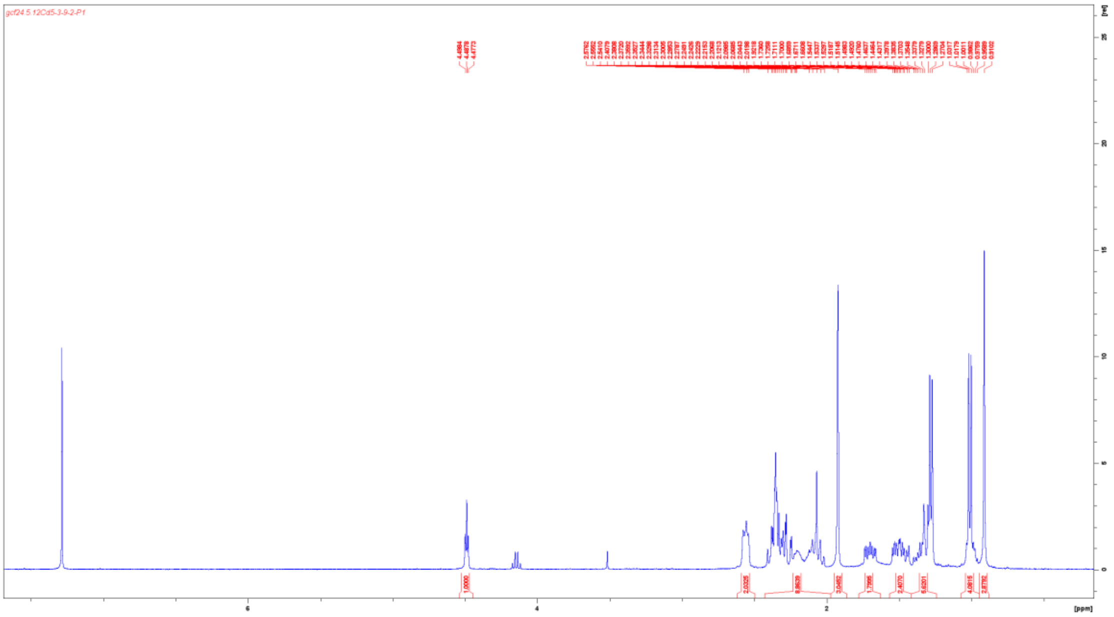

Figure S11. ^{1}H NMR spectrum of compound **2** in $CDCl_3$.

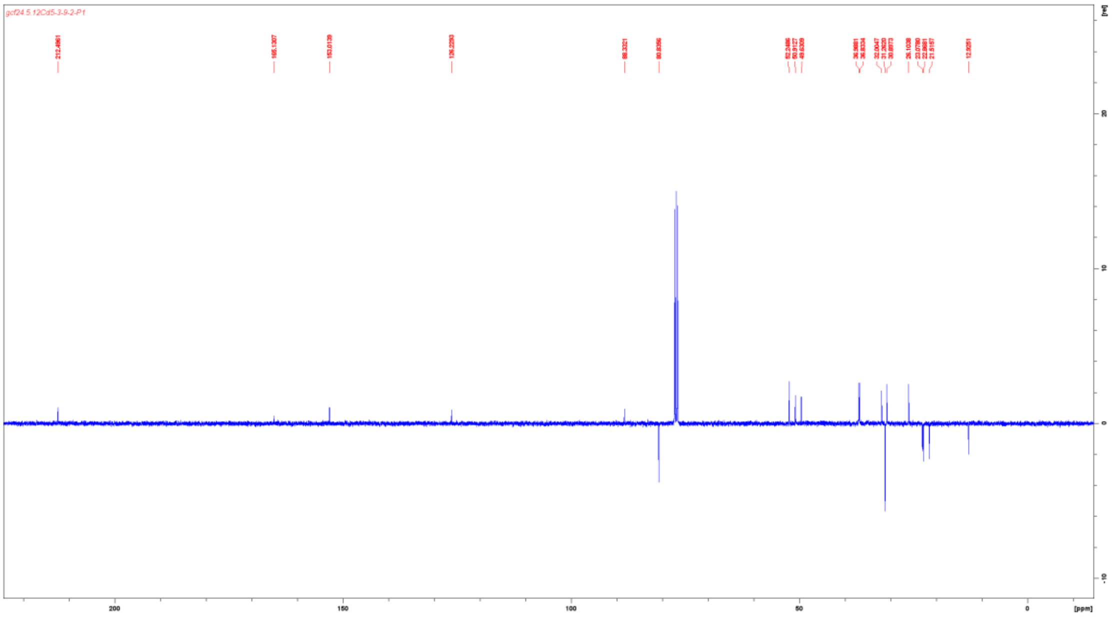

Figure S12. ^{13}C NMR(APT) spectrum of compound **2** in $CDCl_3$.

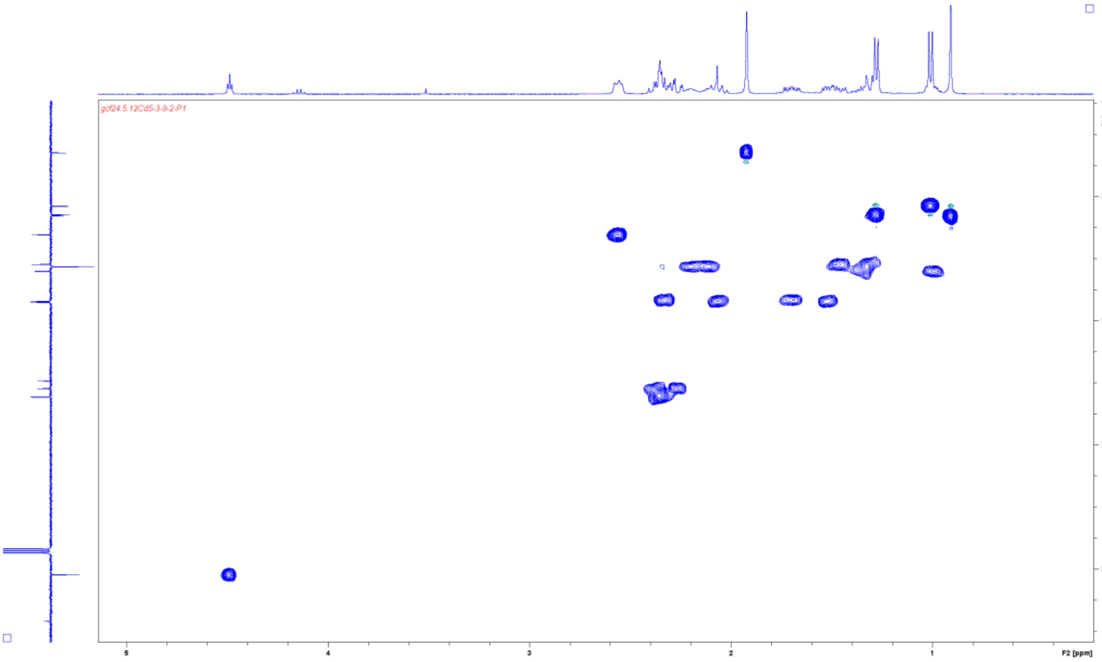

Figure S13. HSQC spectrum of compound **2** in $CDCl_3$.

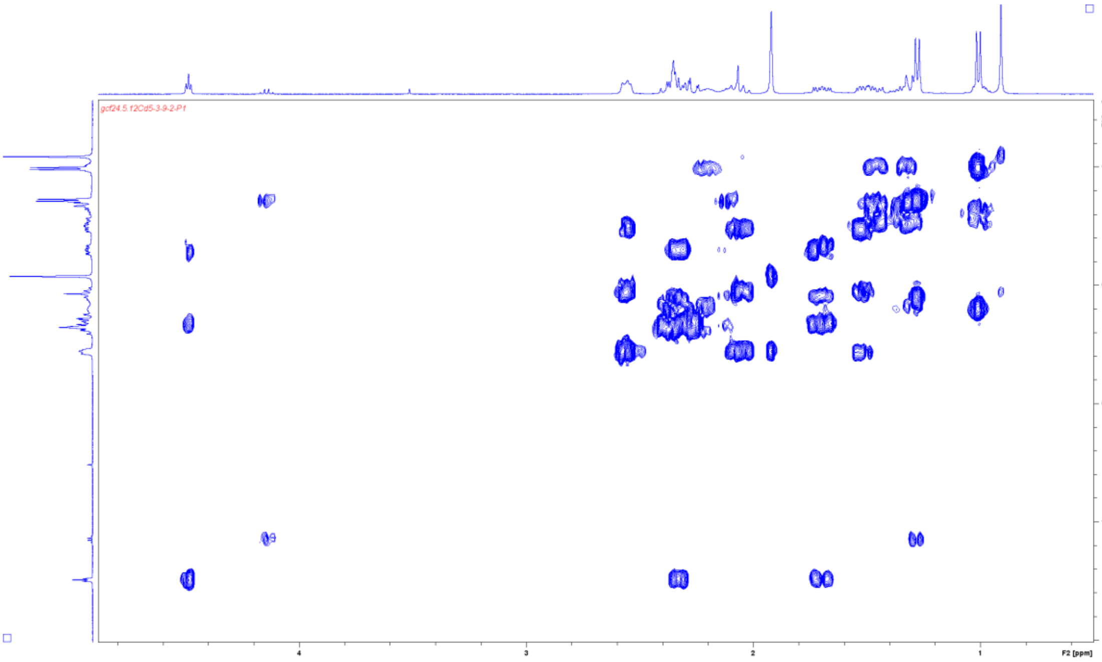

Figure S14. ^{1}H-^{1}H-COSY spectrum of compound **2** in $CDCl_3$.

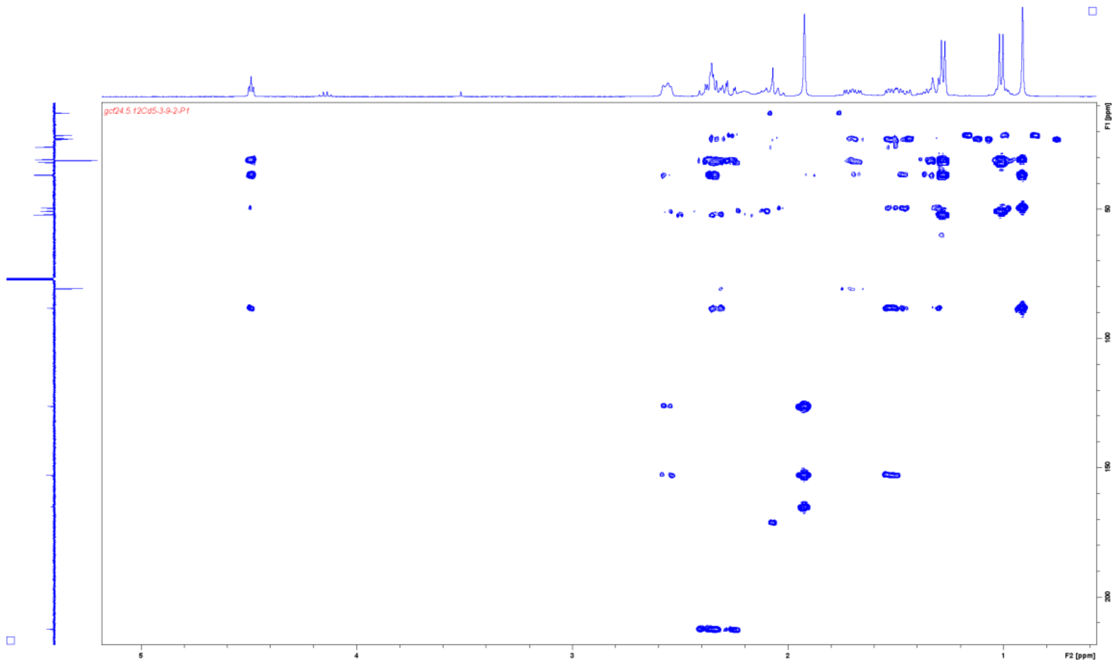

Figure S15. HMBC spectrum of compound **2** in $CDCl_3$.

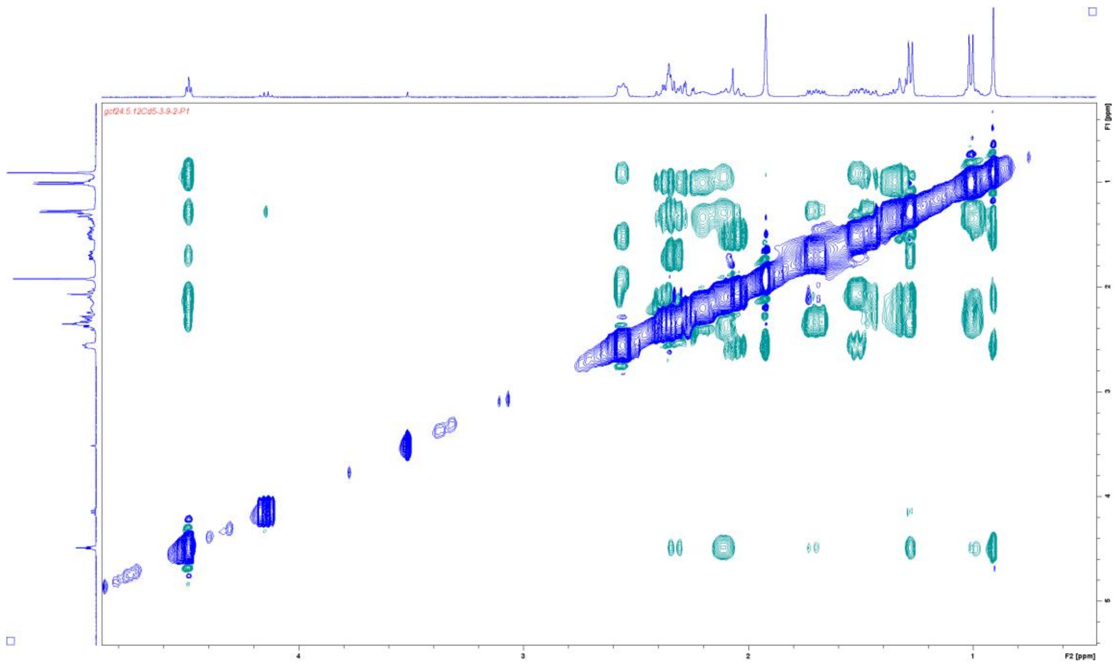

Figure S16. NOESY spectrum of compound **2** in $CDCl_3$.

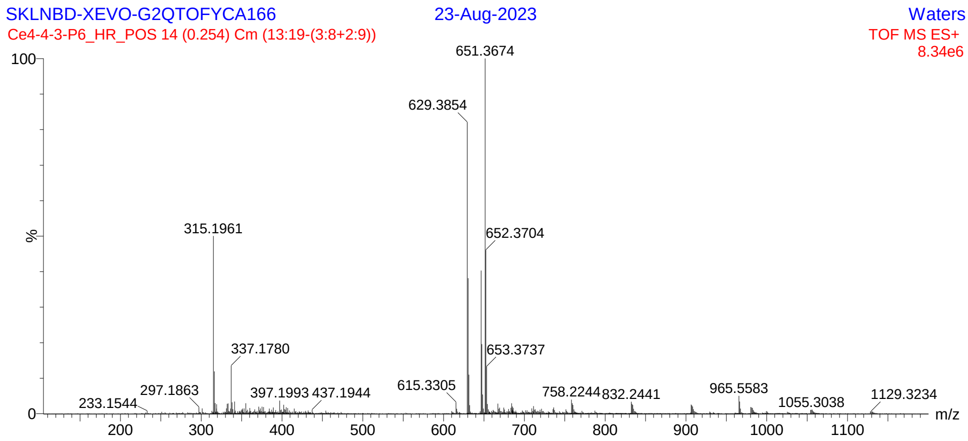

Mass	Calc. Mass	mDa	PPM	DBE	i-FIT	Norm	Conf(%)	Formula
315.1961	315.1960	0.1	7.5	0.0	186.0	n/a	n/a	$C_{20}H_{27}O_3$

Figure S17. HRESIMS spectrum of compound **3**.

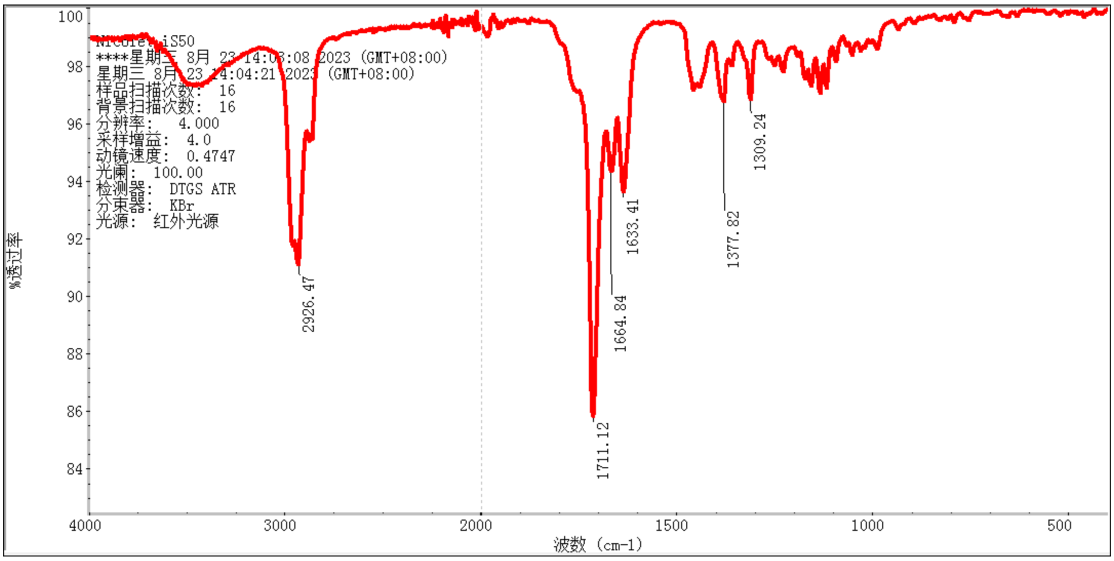

Figure S18. IR spectrum of compound **3**.

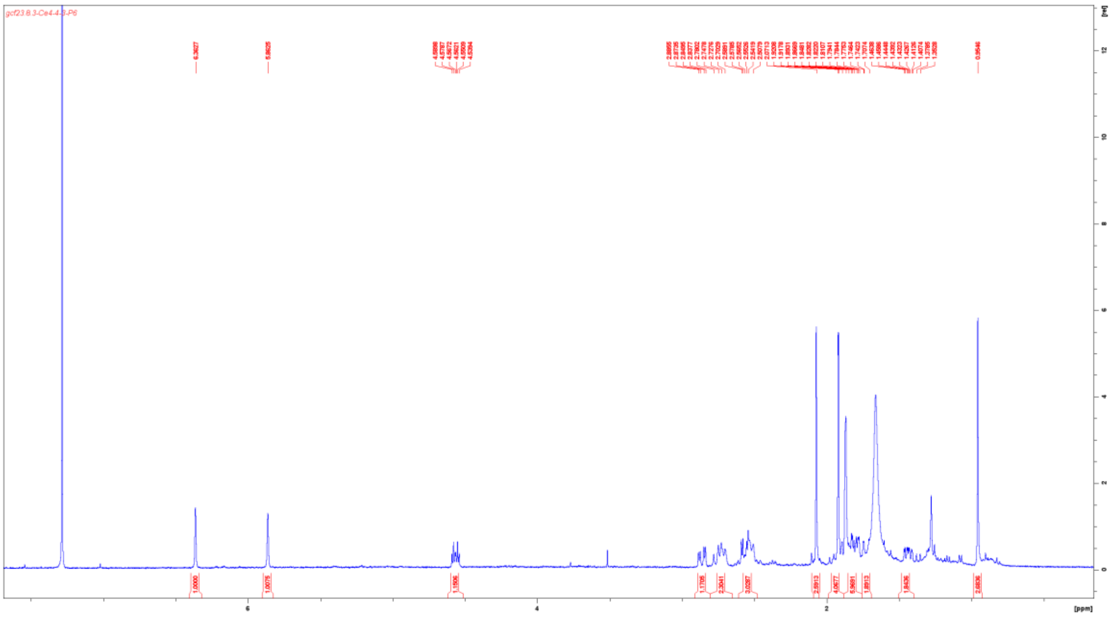

Figure S19. ^{1}H NMR spectrum of compound **3** in $CDCl_3$.

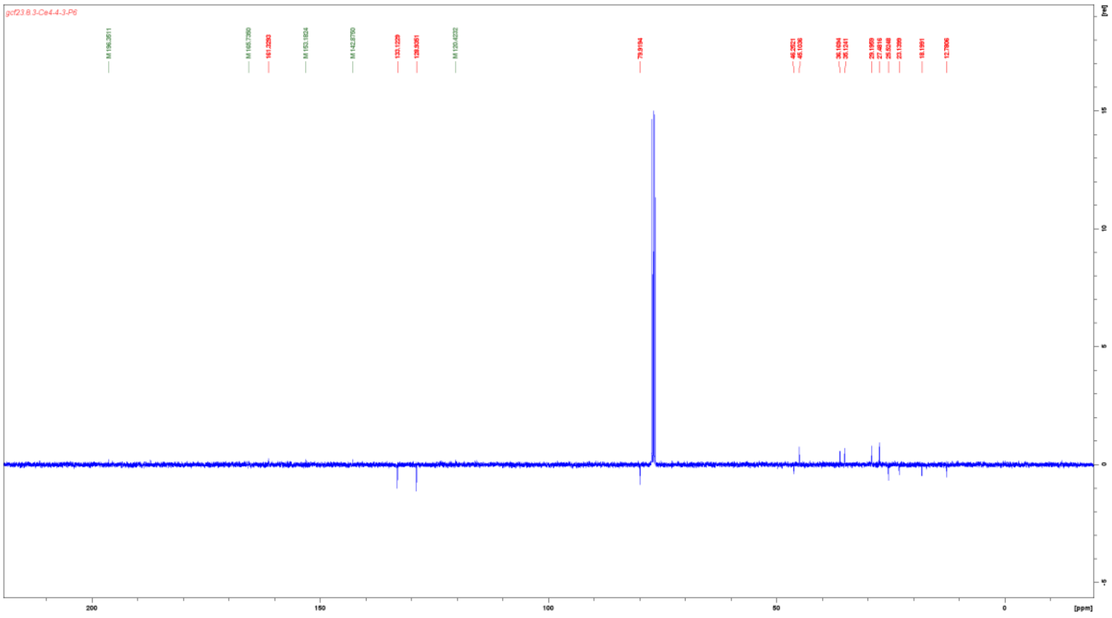

Figure S20. ^{13}C NMR(APT) spectrum of compound **3** in $CDCl_3$.

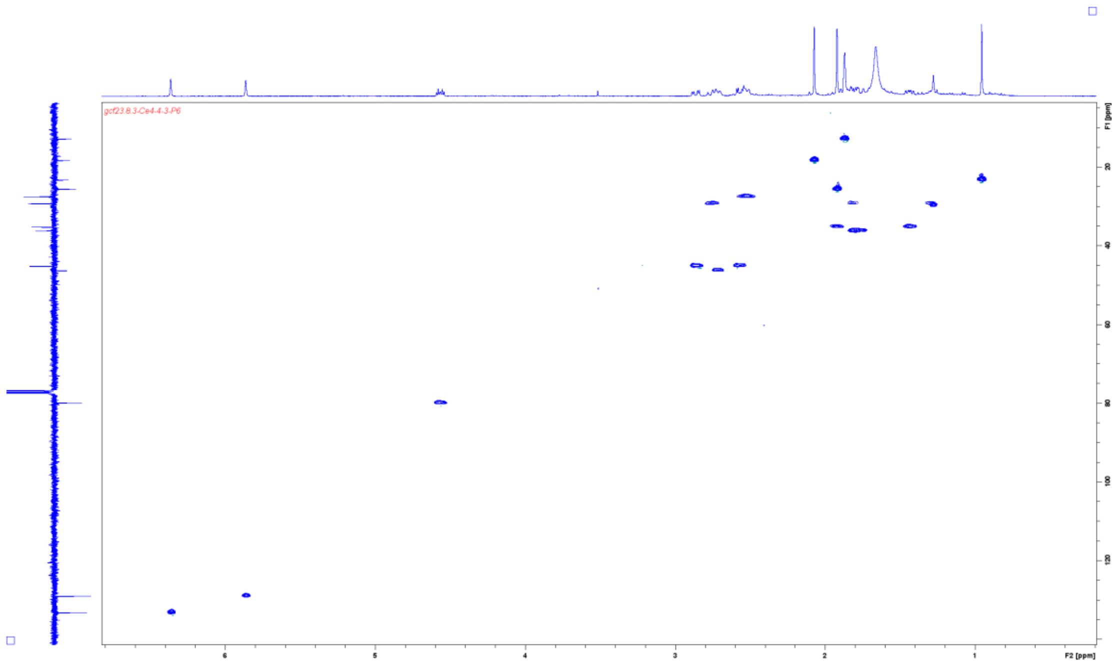

Figure S21 HSQC spectrum of compound **3** in $CDCl_3$.

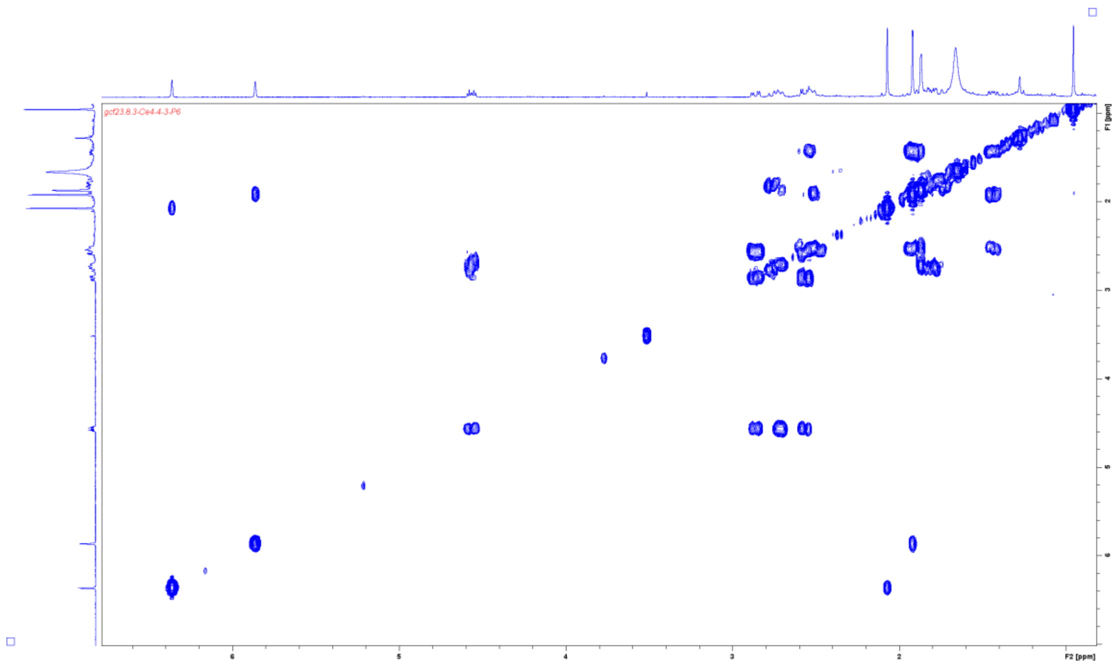

Figure S22. ^1H-^1H-COSY spectrum of compound **3** in $CDCl_3$.

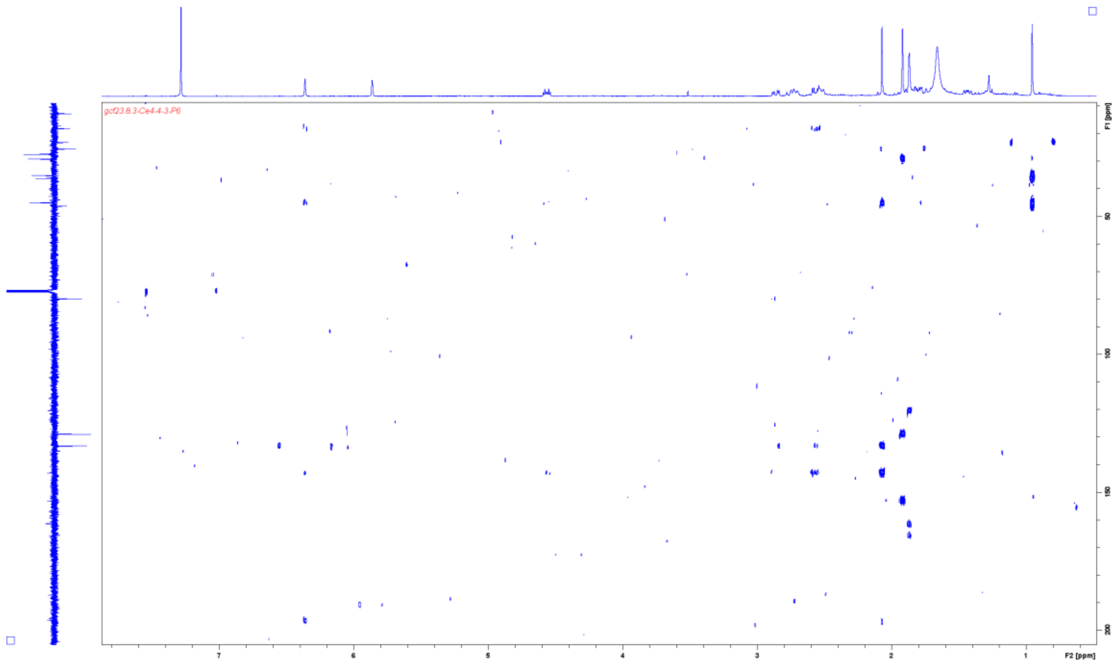

Figure S23. HMBC spectrum of compound **3** in $CDCl_3$.

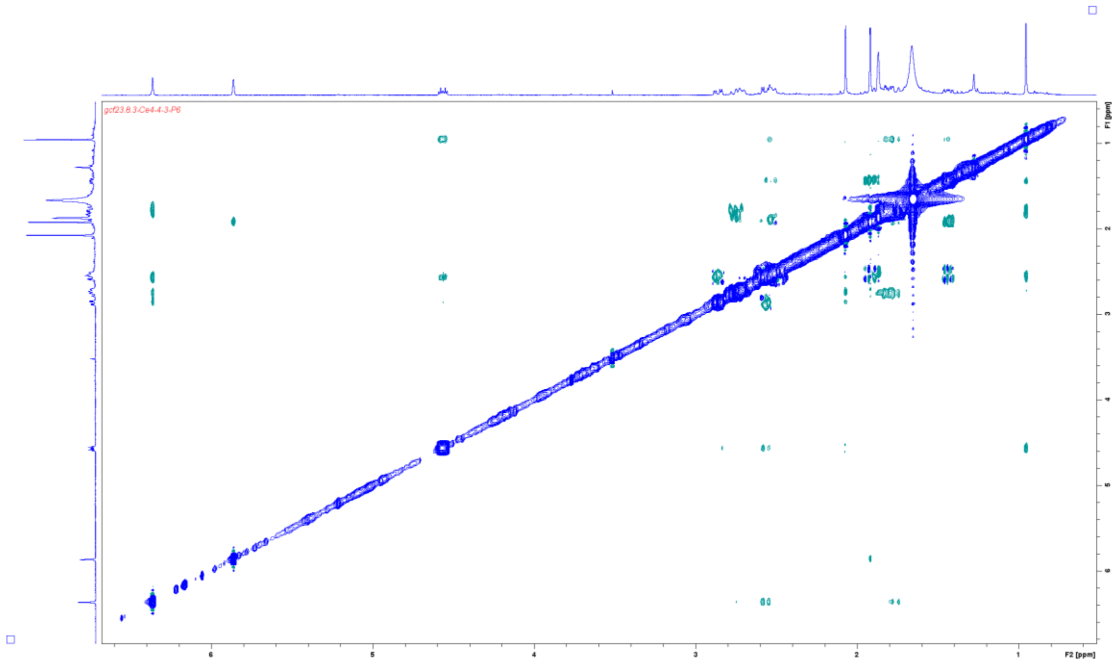

Figure S24. NOESY spectrum of compound **3** in $CDCl_3$.

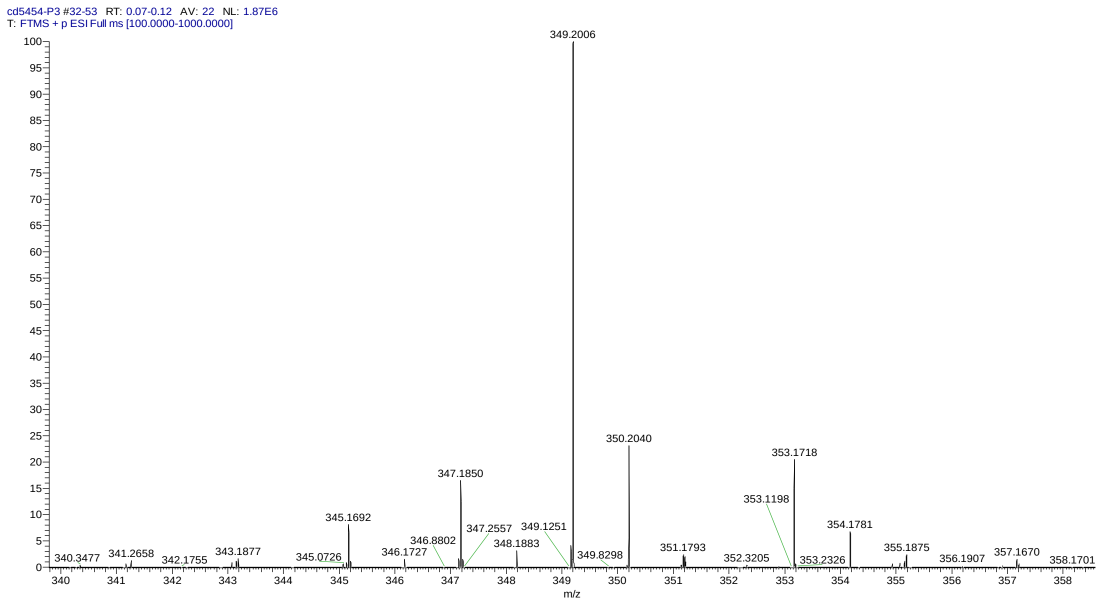

Mass	Calc. Mass	mDa	PPM	DBE	i-FIT	Norm	Conf(%)	Formula
349.2006	349.2009	0.0	-1.0	6.5	191.3	n/a	n/a	$C_{20}H_{29}O_5$

Figure S25. HRESIMS spectrum of compound **4**.

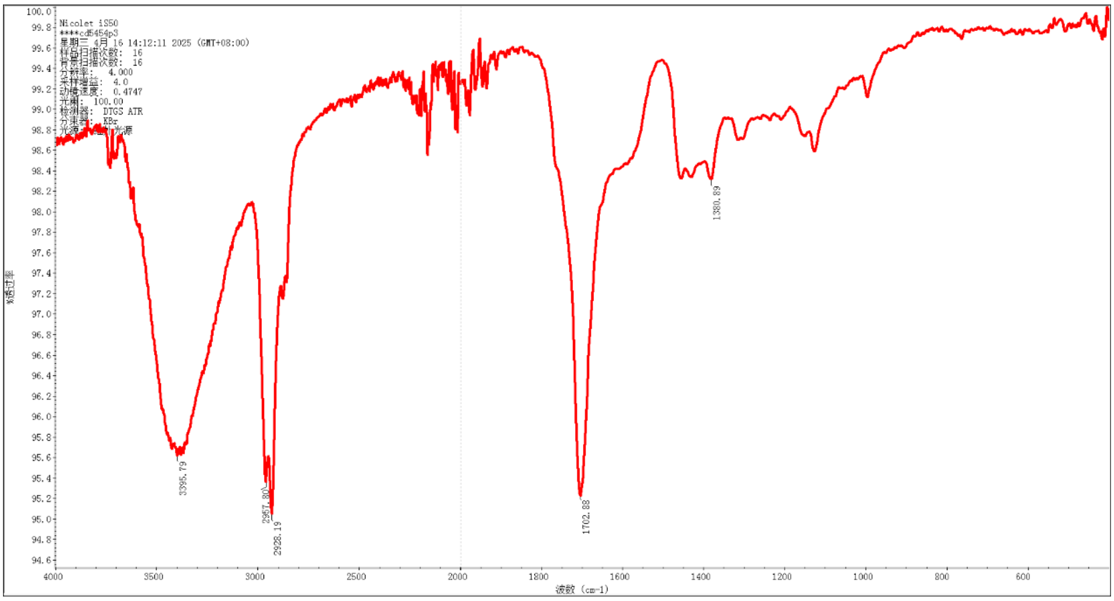

Figure S26. IR spectrum of compound **4**.

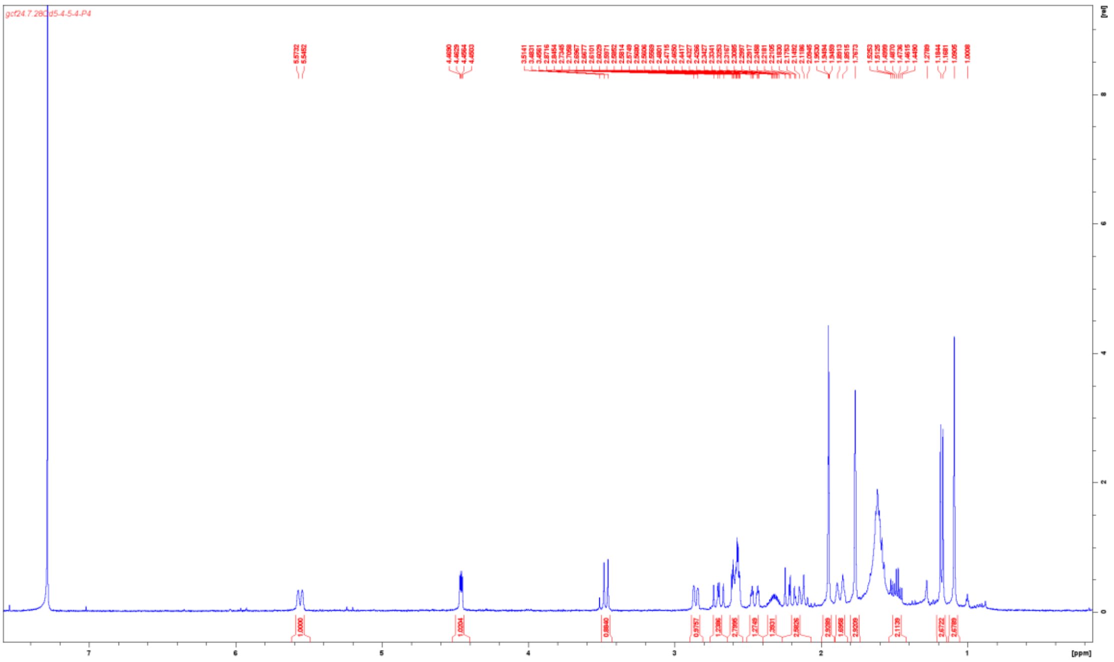

Figure S27. ^{1}H NMR spectrum of compound **4** in $CDCl_3$.

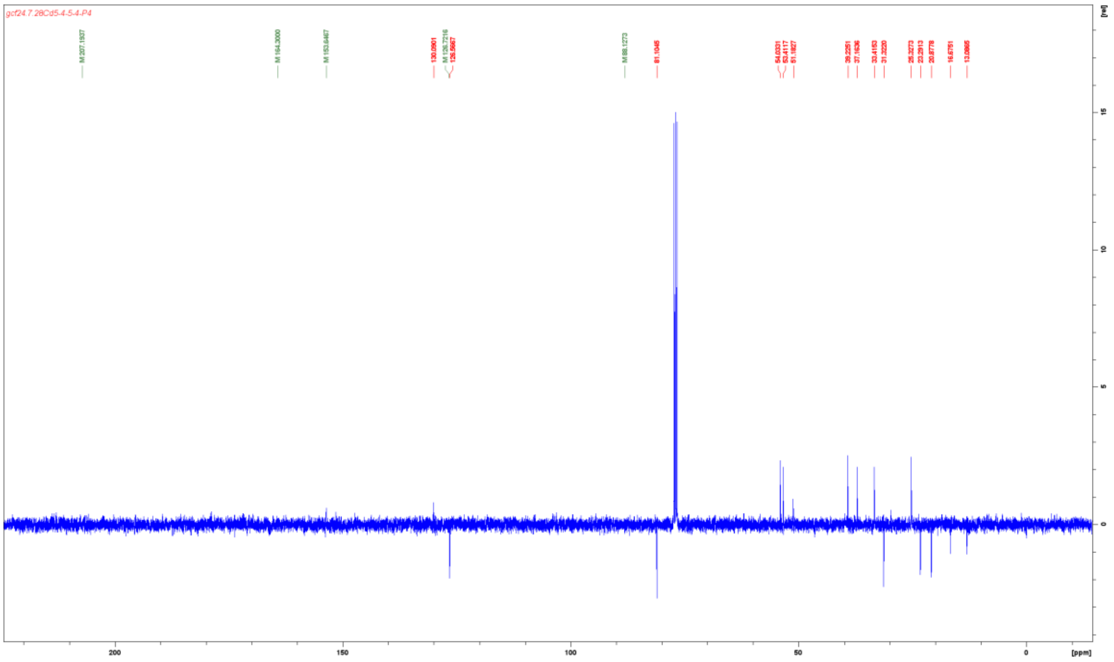

Figure S28. ^{13}C NMR(APT) spectrum of compound **4** in $CDCl_3$.

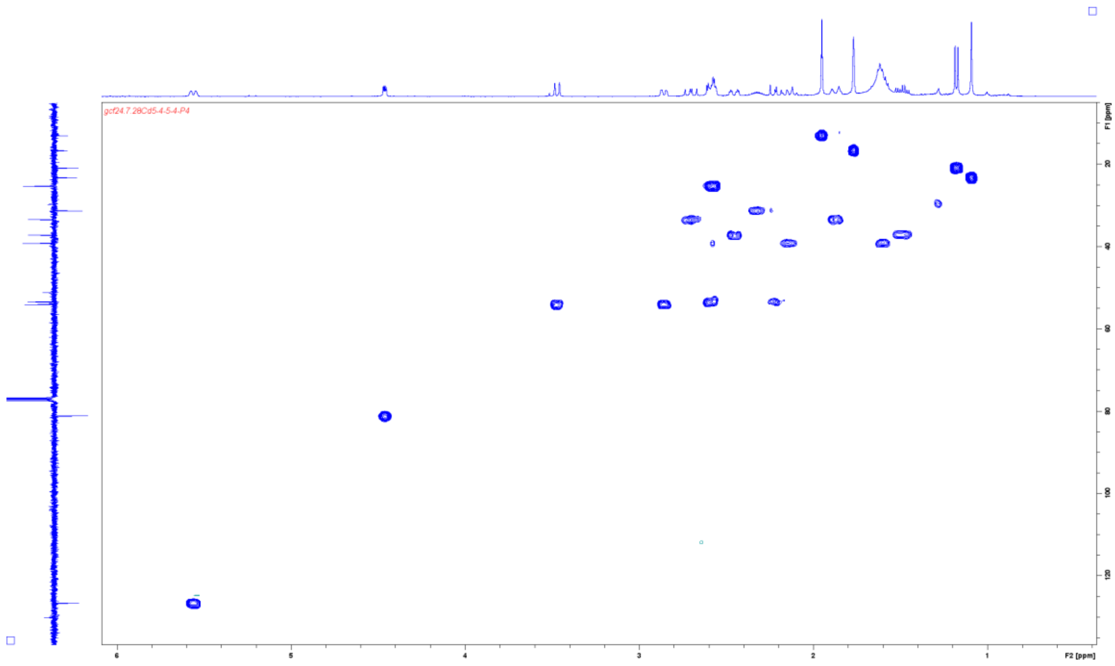

Figure S29 HSQC spectrum of compound **4** in $CDCl_3$.

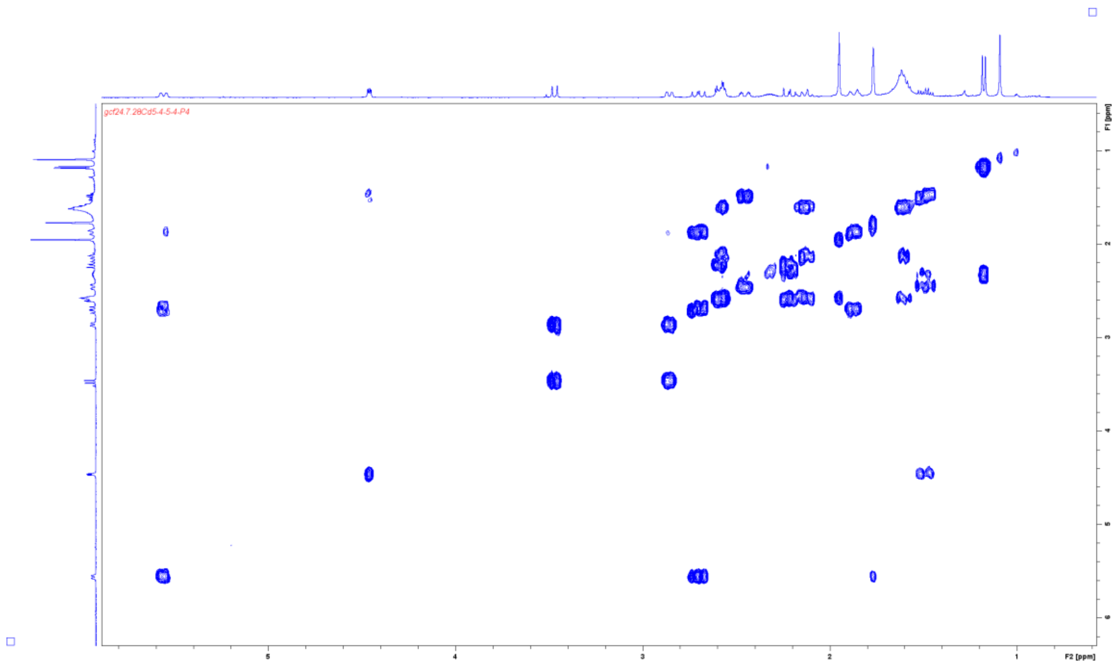

Figure S30. ^{1}H-^{1}H-COSY spectrum of compound **4** in $CDCl_3$.

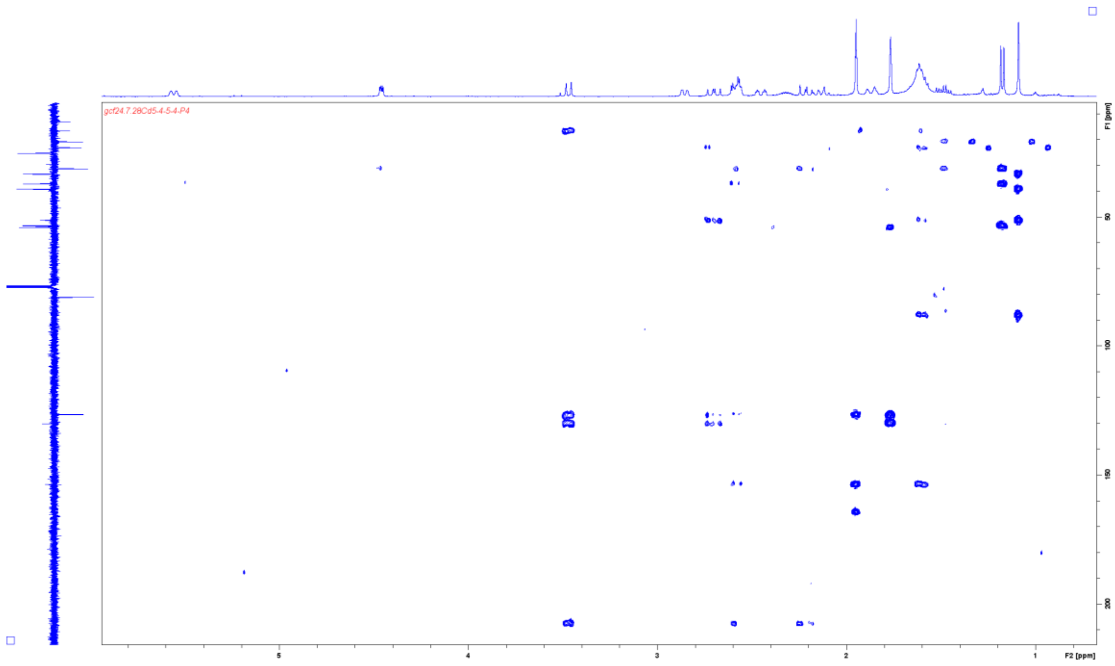

Figure S31. HMBC spectrum of compound **4** in $CDCl_3$.

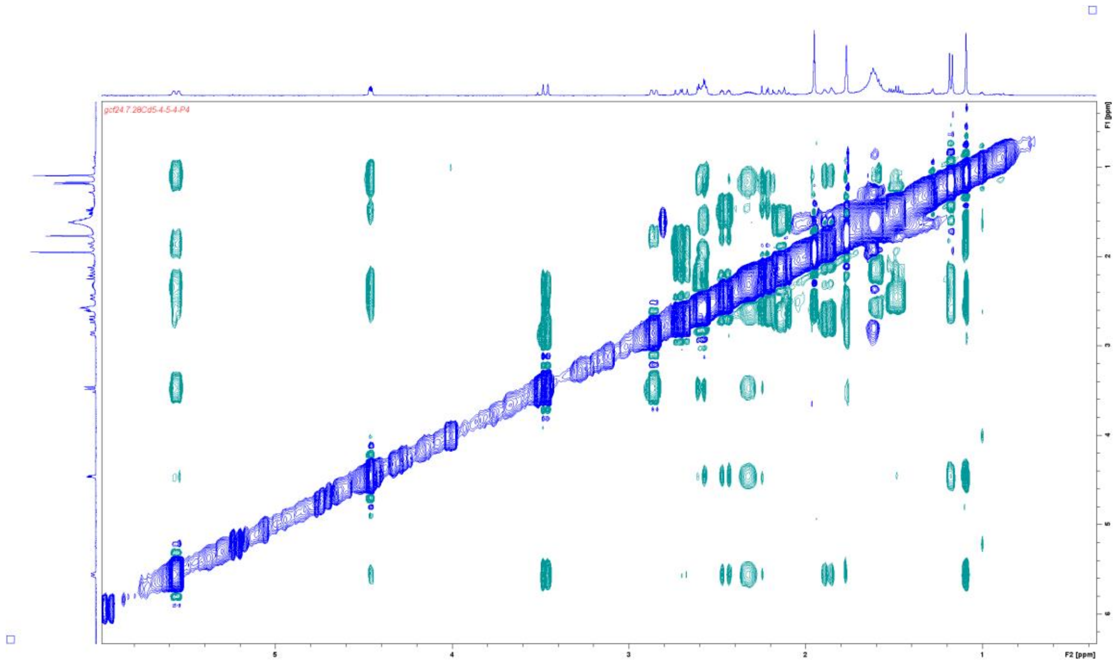

Figure S32. NOESY spectrum of compound **4** in $CDCl_3$.

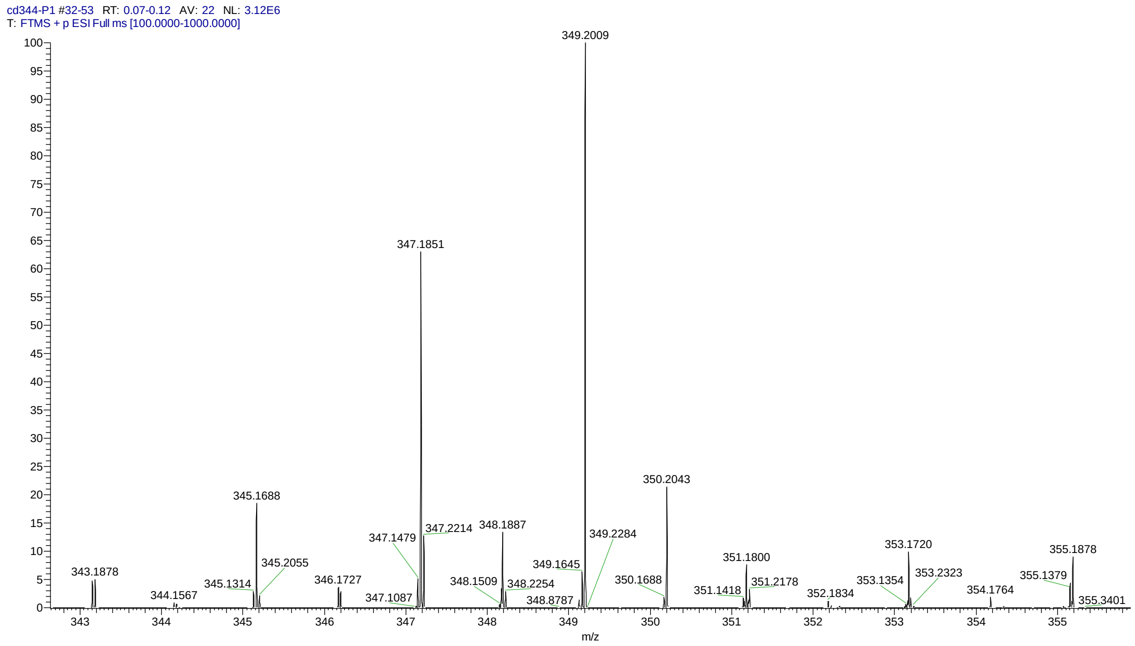

Mass	Calc. Mass	mDa	PPM	DBE	i-FIT	Norm	Conf(%)	Formula
349.2009	349.2009	0.0	-0.1	6.5	9.0	n/a	n/a	$C_{20}H_{29}O_5$

Figure S33. HRESIMS spectrum of compound **5**.

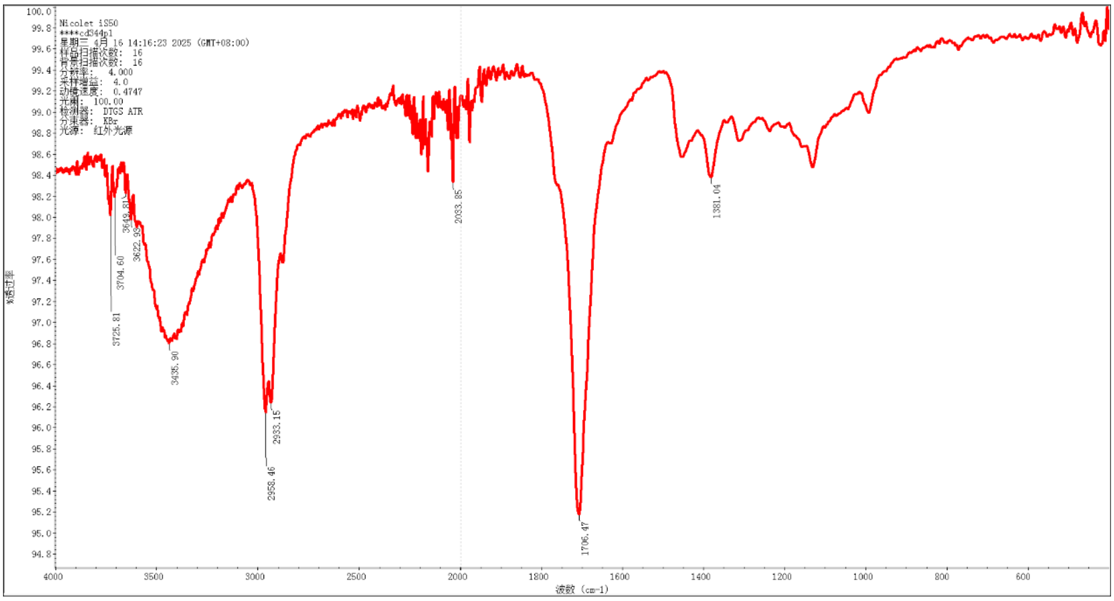

Figure S34. IR spectrum of compound **5**.

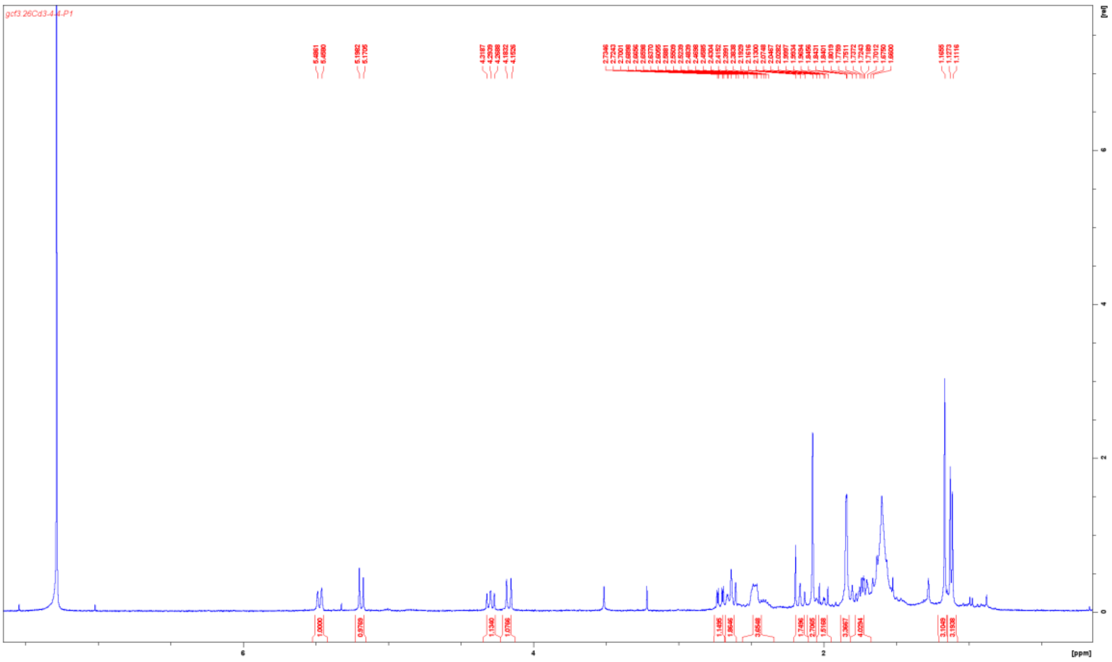

Figure S35. ^{1}H NMR spectrum of compound **5** in $CDCl_3$.

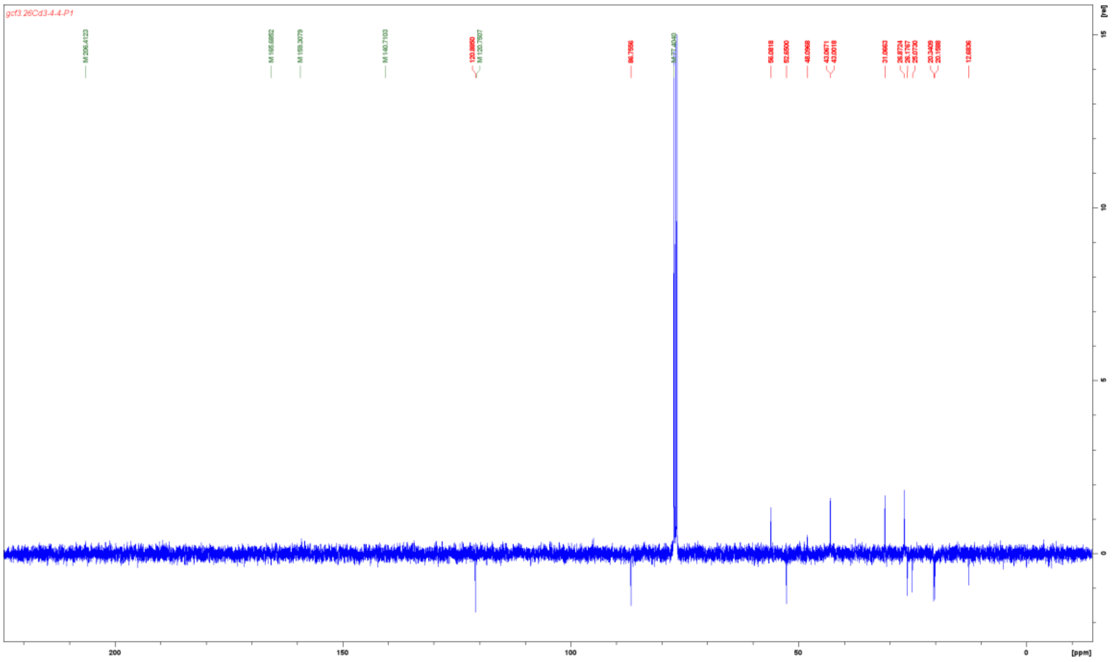

Figure S36. ^{13}C NMR(APT) spectrum of compound **5** in $CDCl_3$.

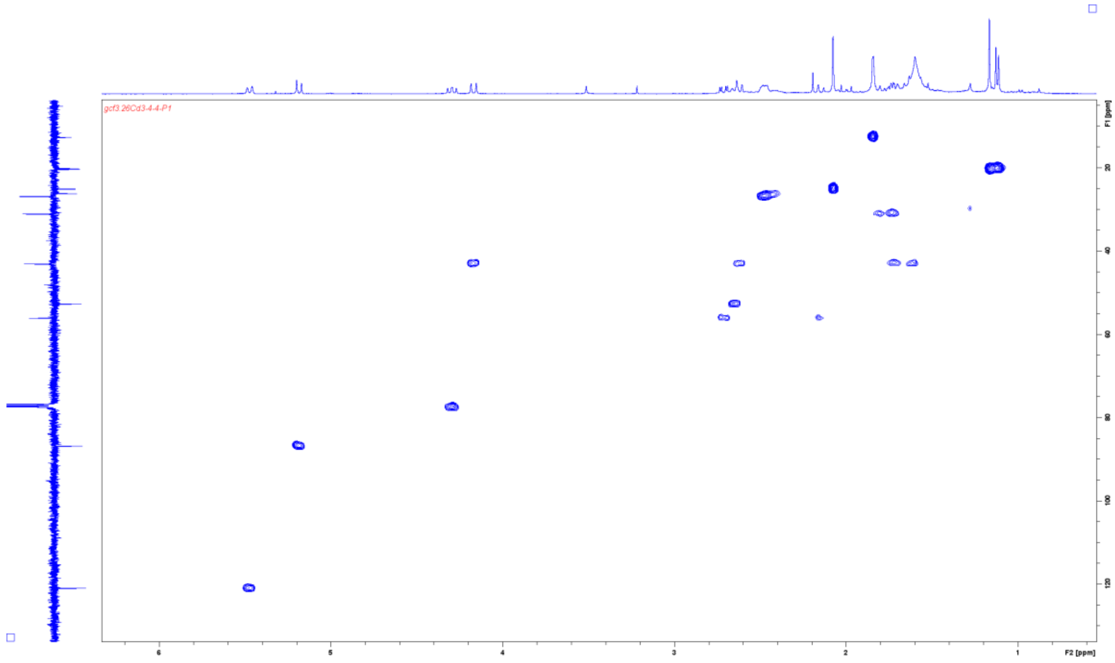

Figure S37 HSQC spectrum of compound **5** in $CDCl_3$.

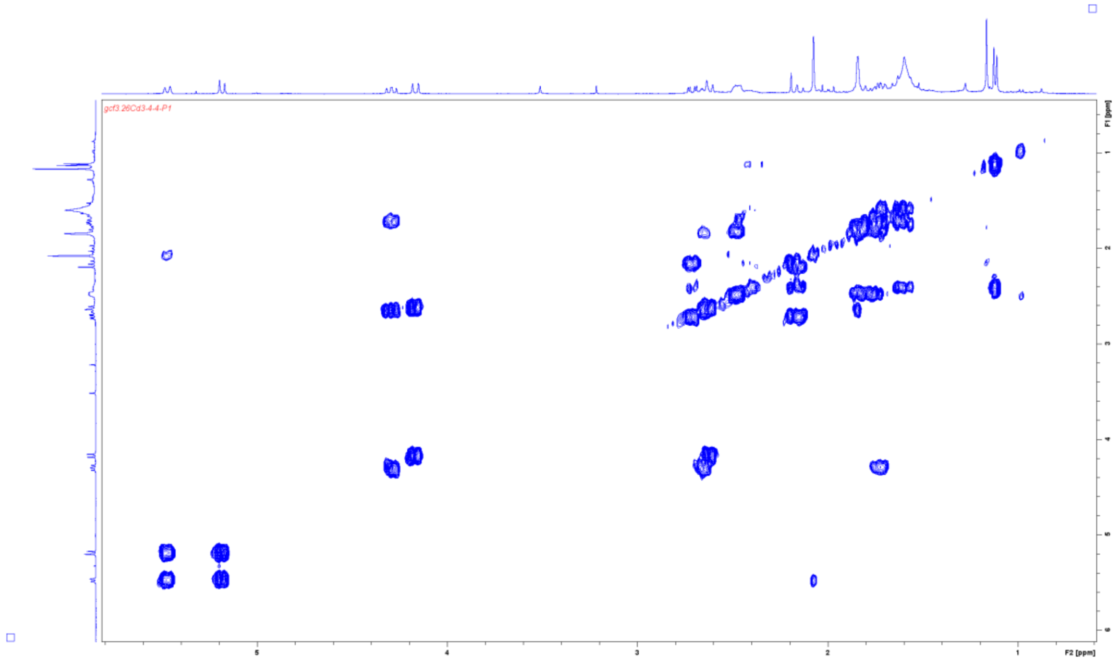

Figure S38. ^1H-^1H-COSY spectrum of compound **5** in $CDCl_3$.

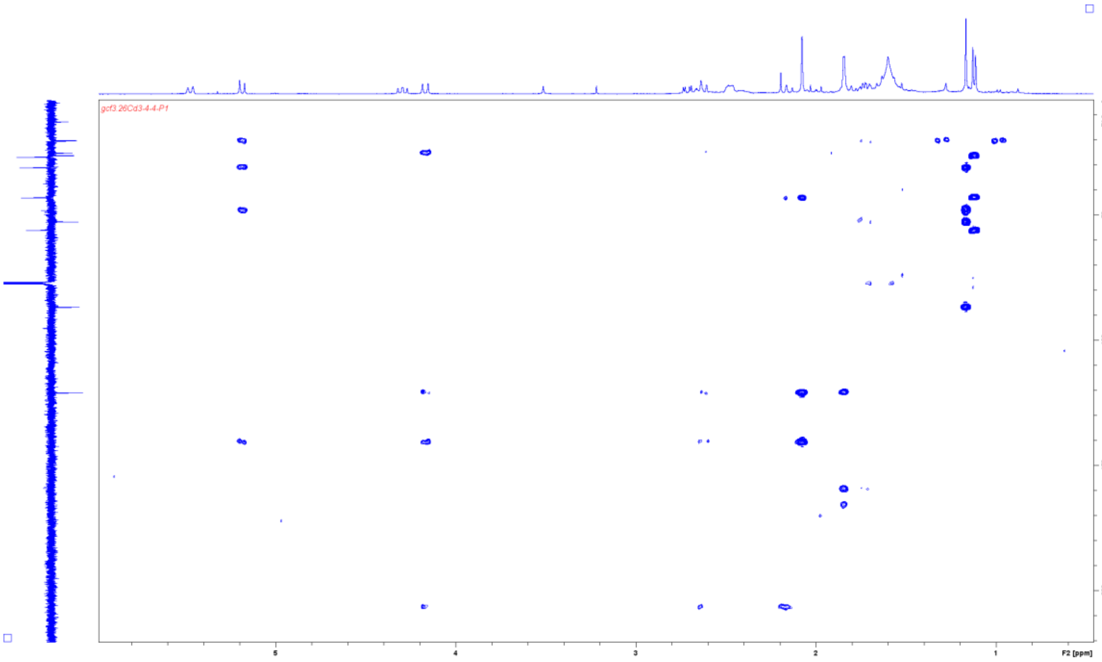

Figure S39. HMBC spectrum of compound **5** in $CDCl_3$.

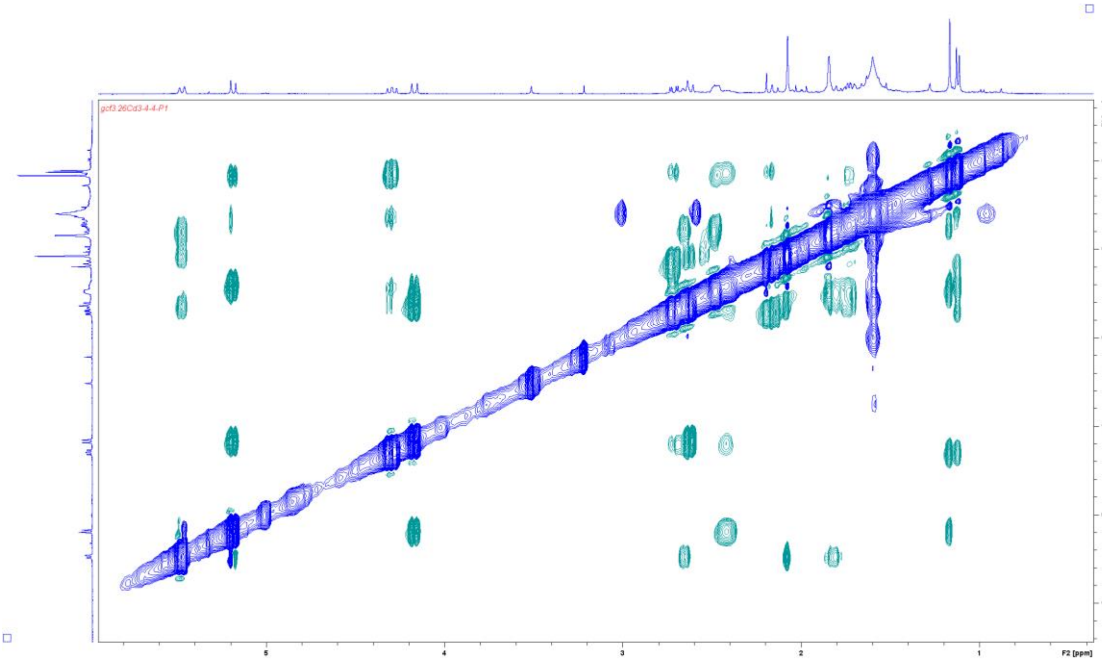

Figure S40. NOESY spectrum of compound **5** in $CDCl_3$.

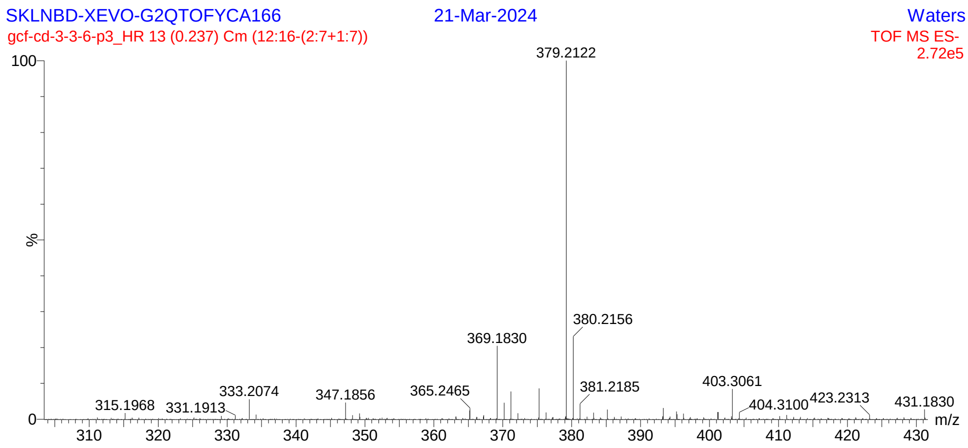

Mass	Calc. Mass	mDa	PPM	DBE	i-FIT	Norm	Conf(%)	Formula
333.2074	333.2066	0.8	6.5	4.5	0.1	n/a	n/a	$C_{20}H_{29}O_4$

Figure S41. HRESIMS spectrum of compound **6**.

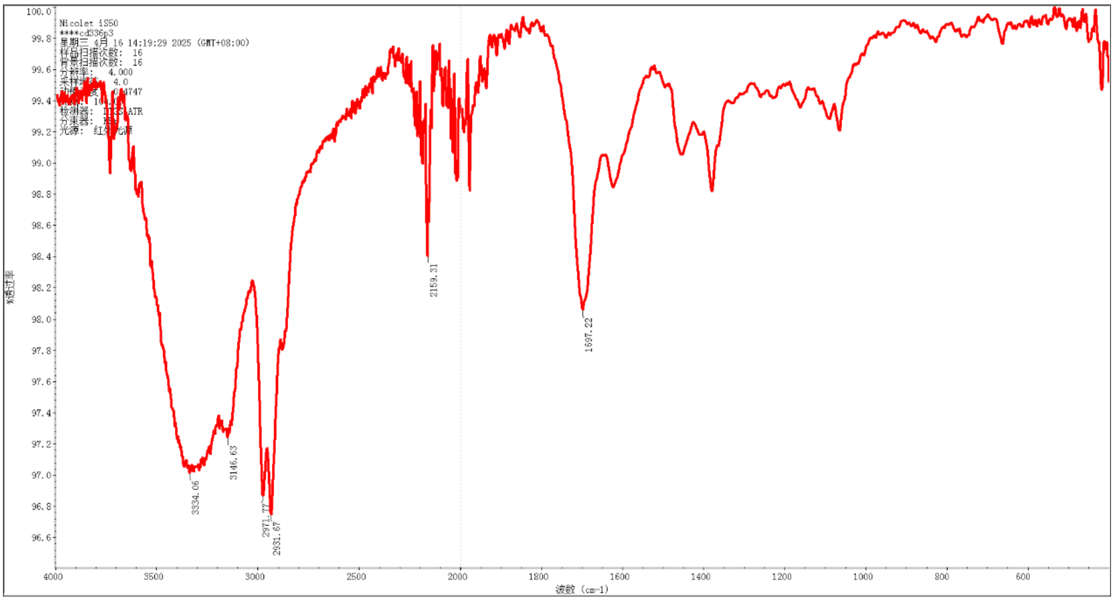

Figure S42. IR spectrum of compound **6**.

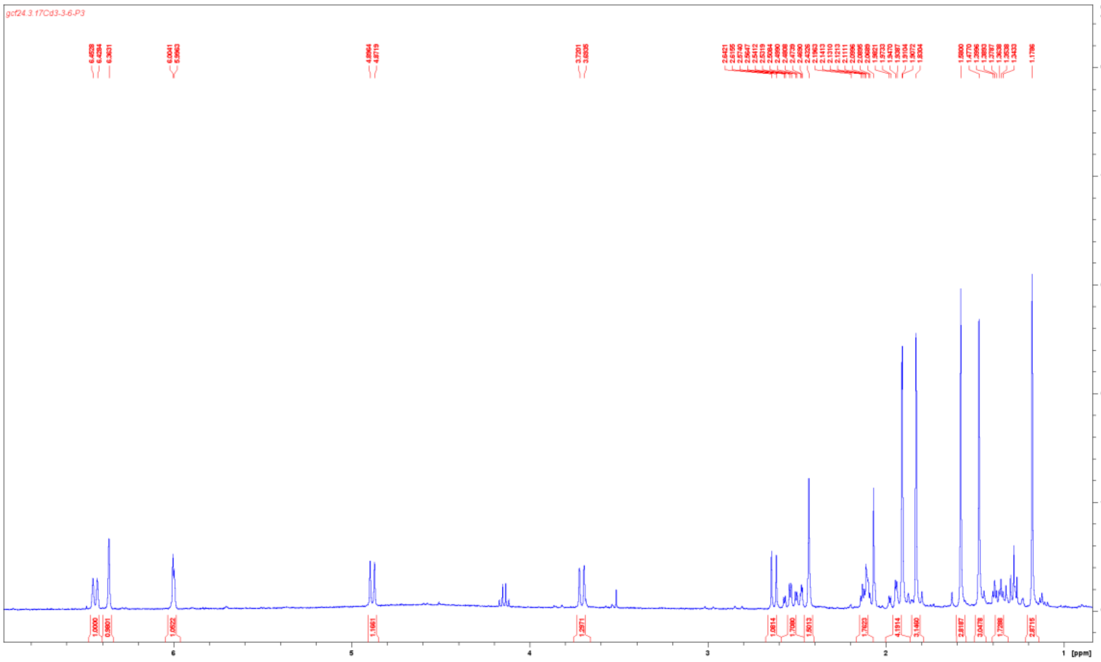

Figure S43. ^{1}H NMR spectrum of compound **6** in $CDCl_3$.

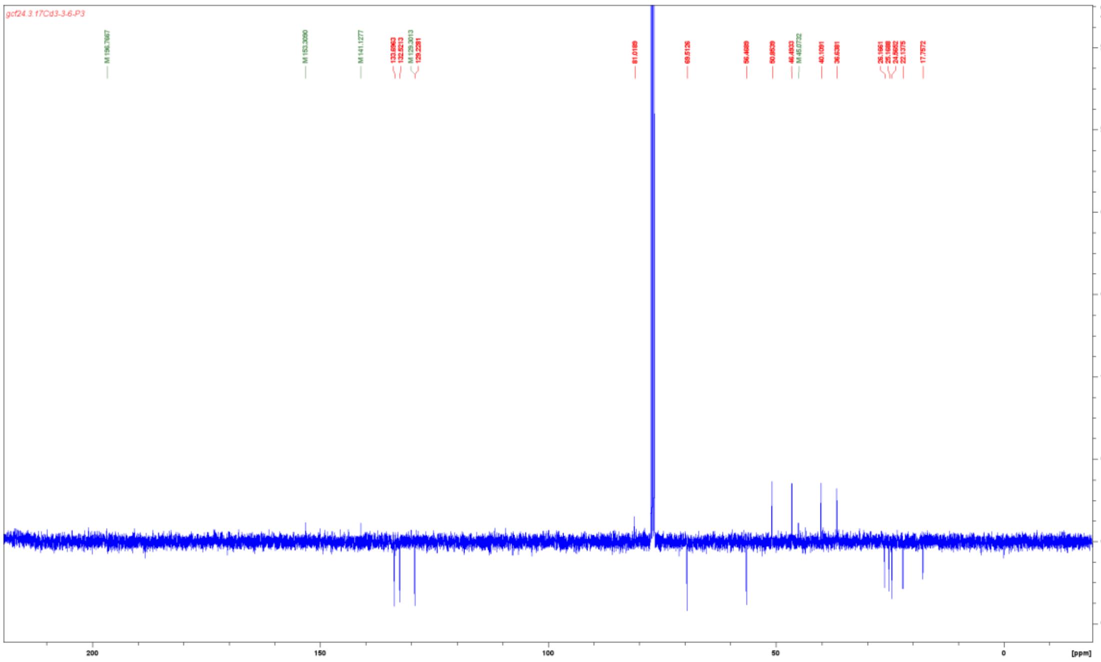

Figure S44. ^{13}C NMR(APT) spectrum of compound **6** in $CDCl_3$.

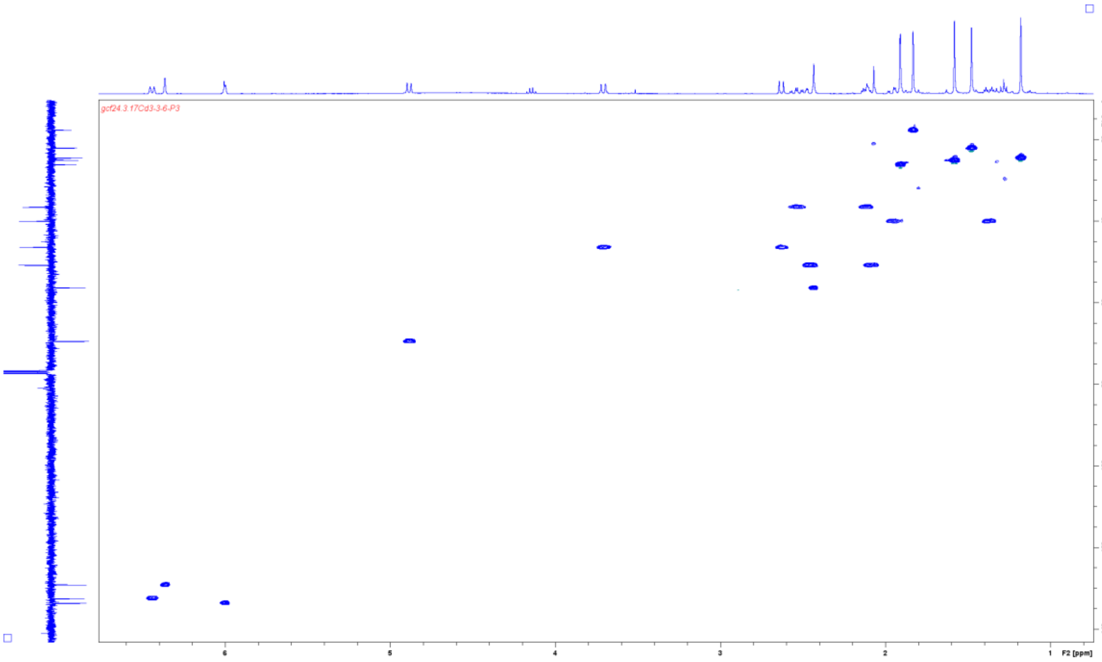

Figure S45 HSQC spectrum of compound **6** in $CDCl_3$.

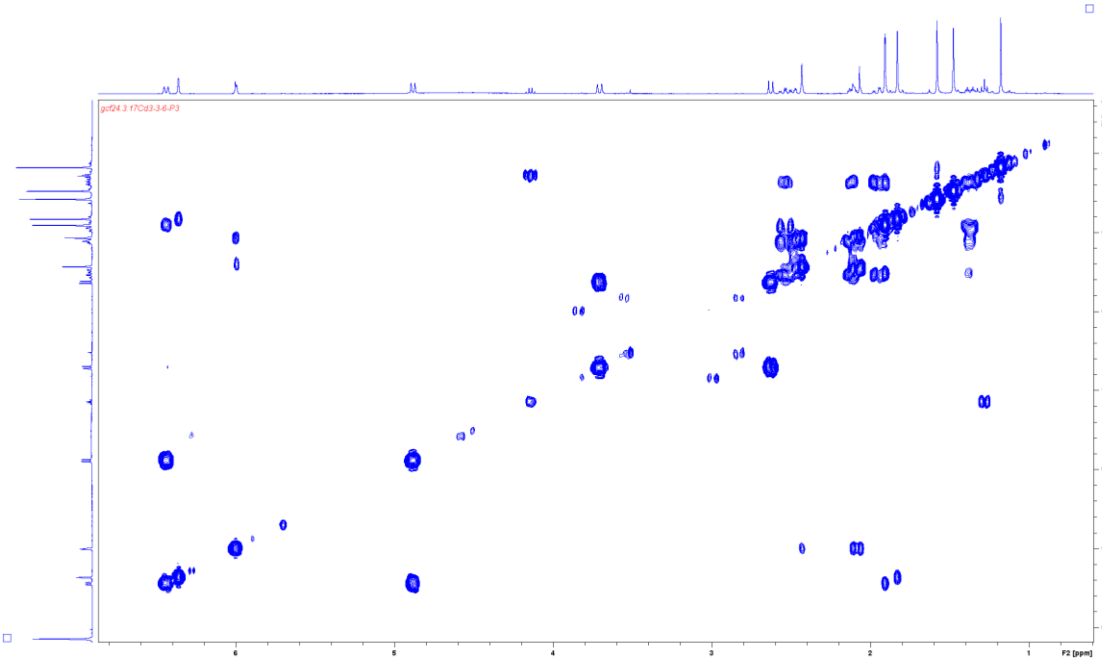

Figure S46. ^{1}H-^{1}H COSY spectrum of compound **6** in $CDCl_3$.

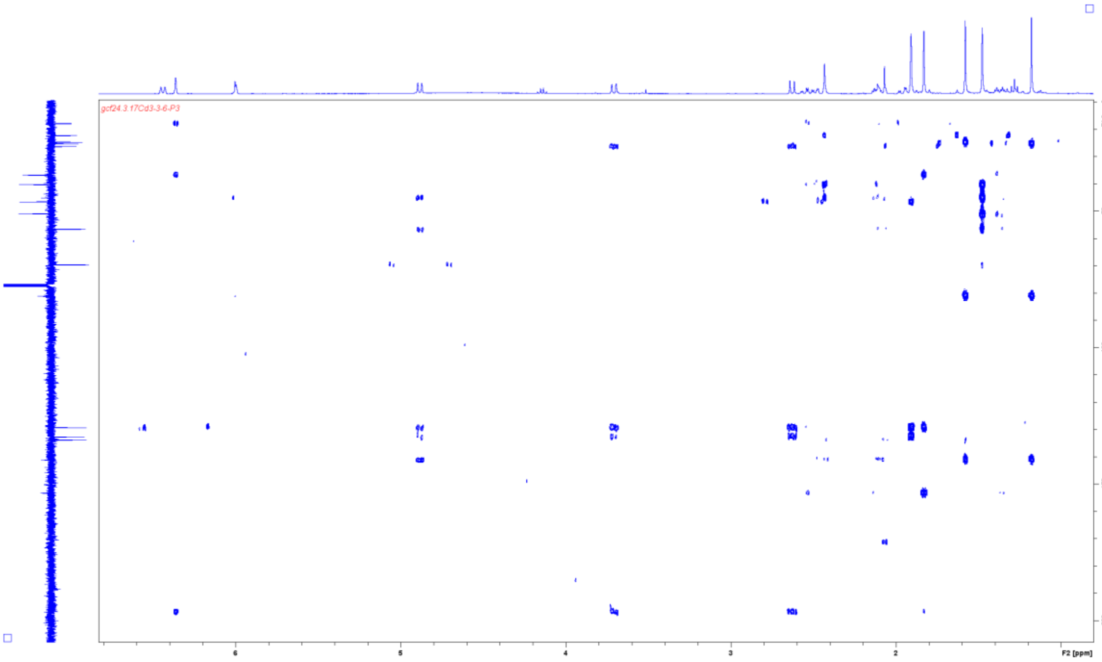

Figure S47. HMBC spectrum of compound **6** in $CDCl_3$.

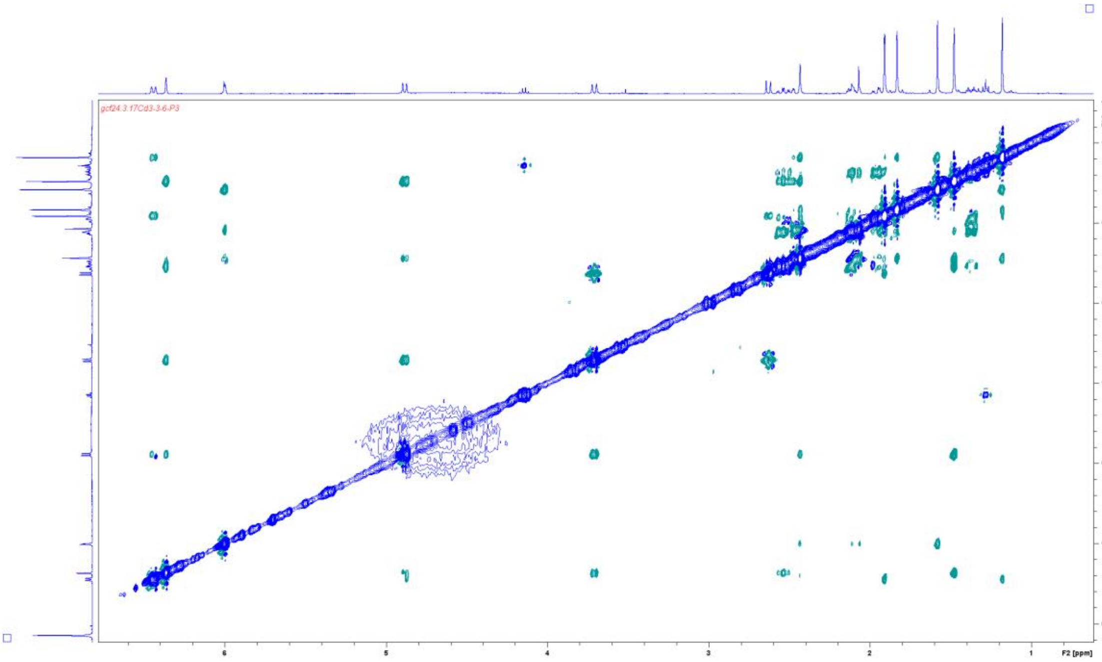

Figure S48. NOESY spectrum of compound **6** in $CDCl_3$.

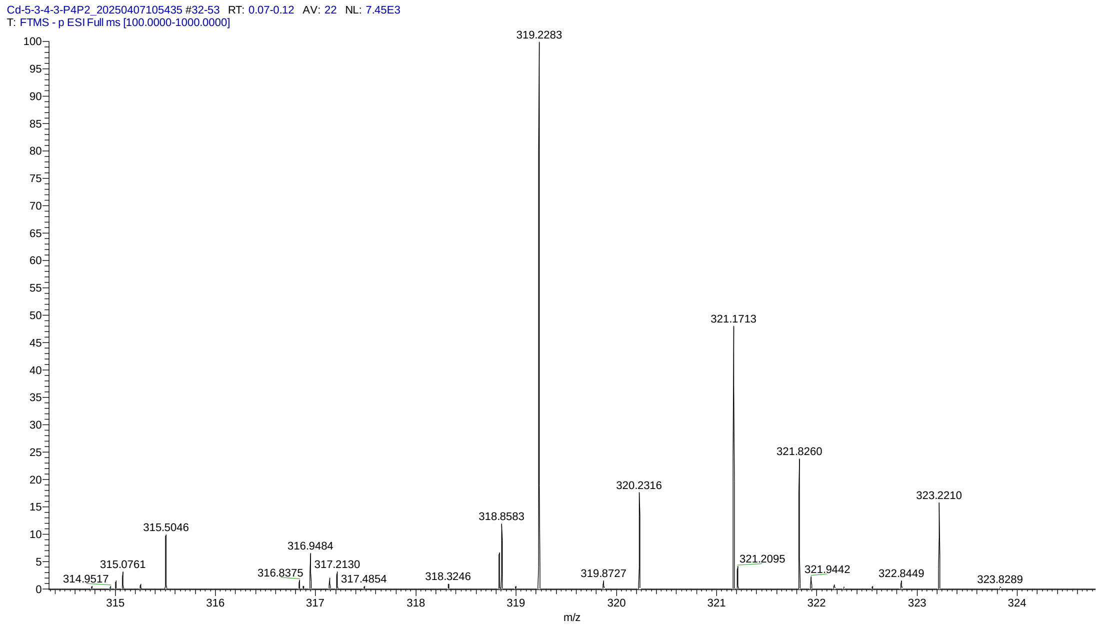

Mass	Calc. Mass	mDa	PPM	DBE	i-FIT	Norm	Conf(%)	Formula
319.2283	319.2278	0.1	1.4	5.5	231.8	n/a	n/a	$C_{20}H_{31}O_3$

Figure S49 HRESIMS spectrum of compound **7**

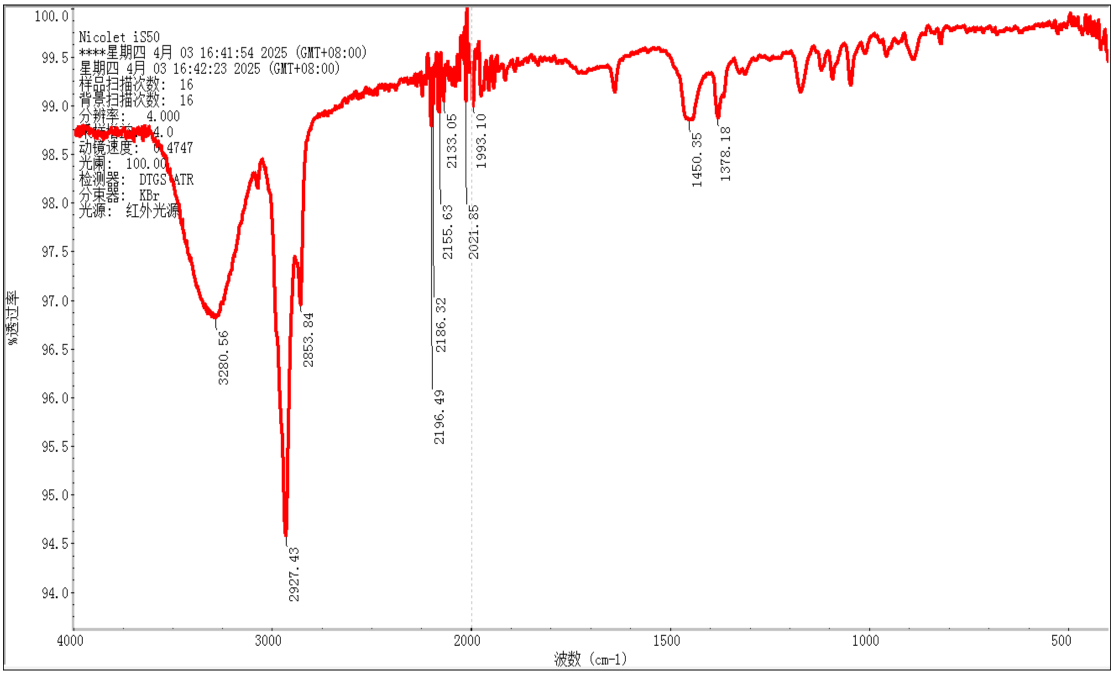

Figure S50 IR spectrum of compound **7**

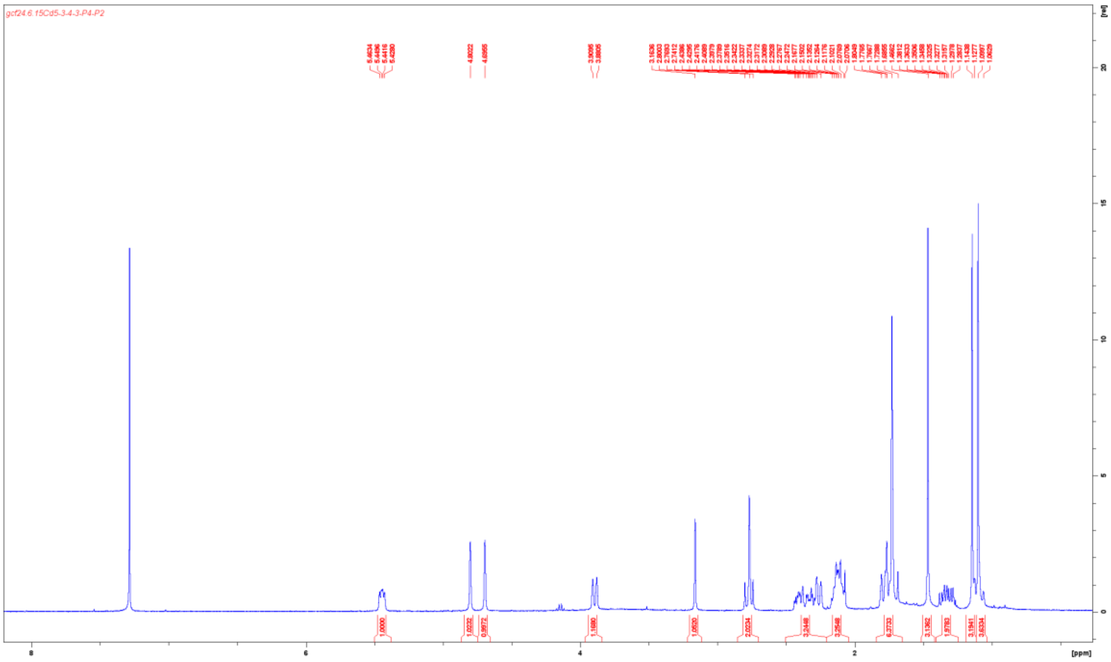

Figure S51 ^{1}H NMR spectrum of compound **7** in $CDCl_3$

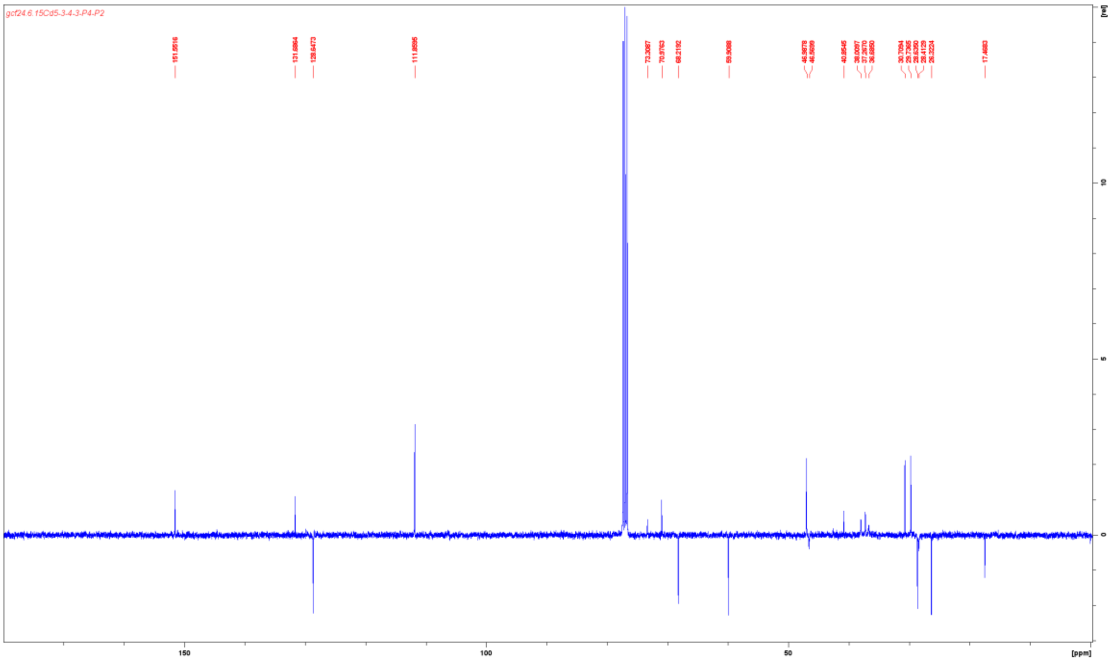

Figure S52 ^{13}C NMR(APT) spectrum of compound **7** in $CDCl_3$

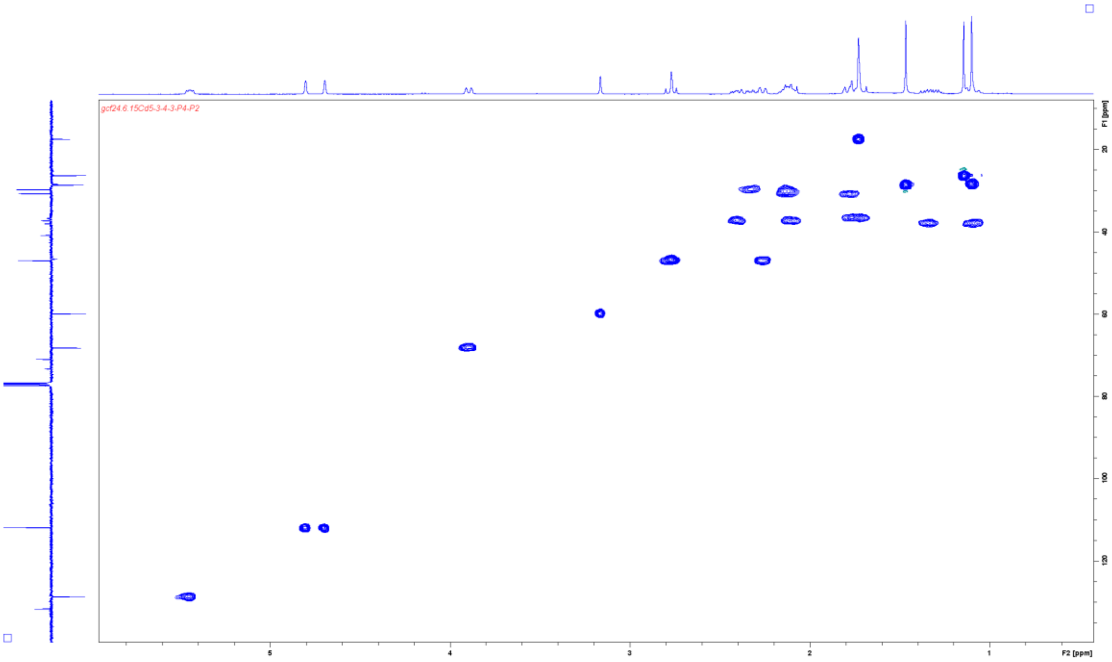

Figure S53 HSQC spectrum of compound **7** in $CDCl_3$

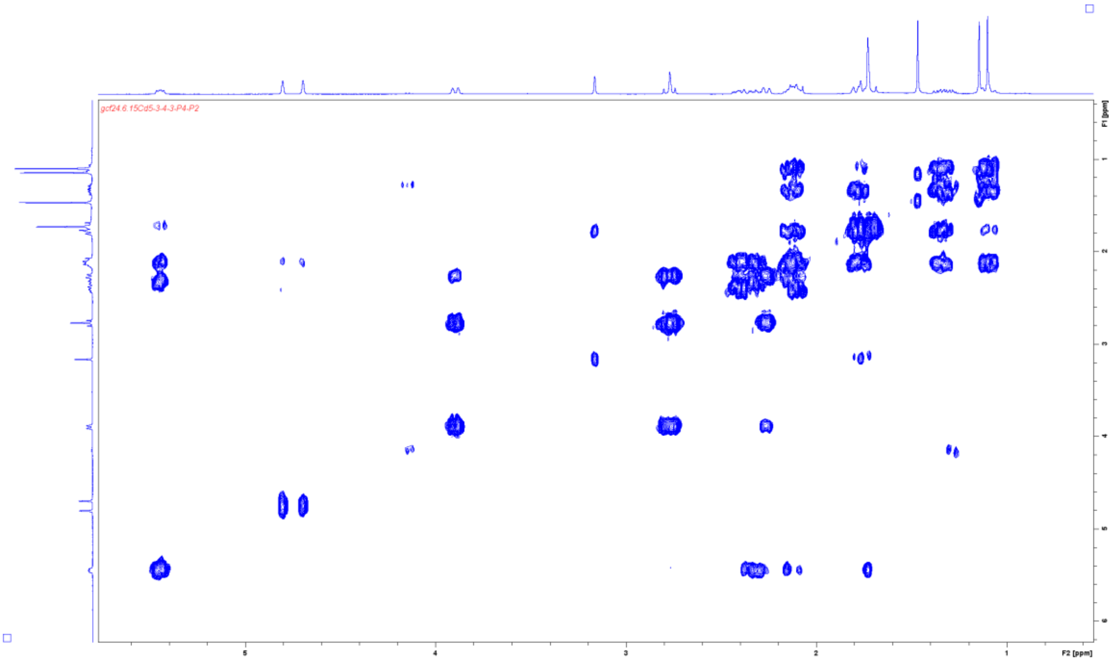

Figure S54 ^1H-^1H COSY spectrum of compound **7** in $CDCl_3$

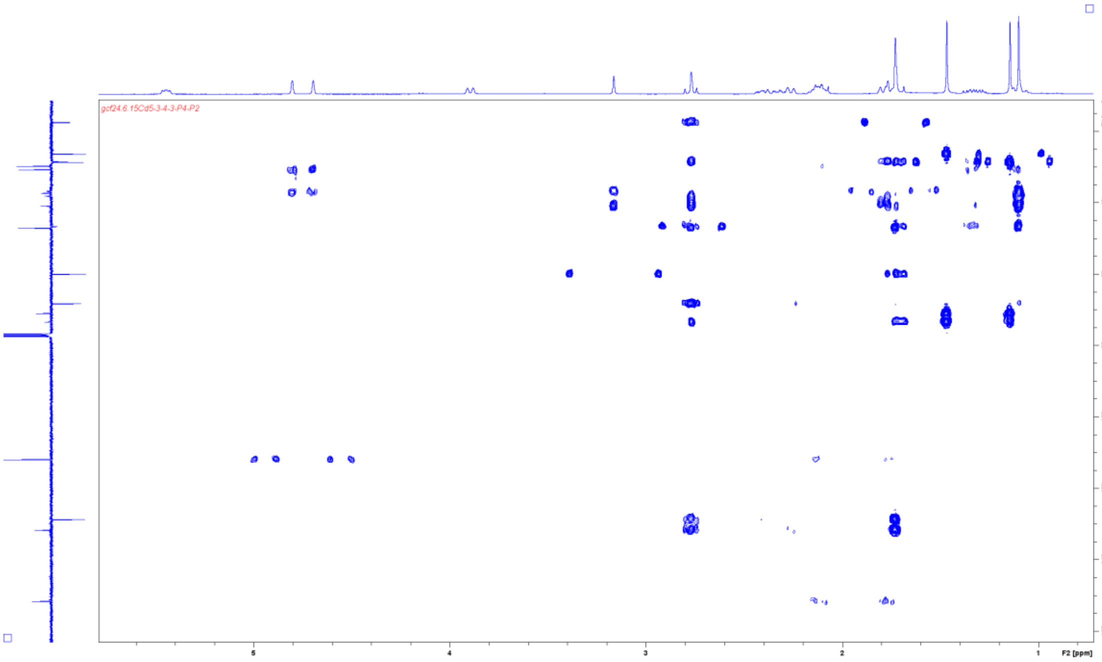

Figure S55 HMBC spectrum of compound **7** in $CDCl_3$

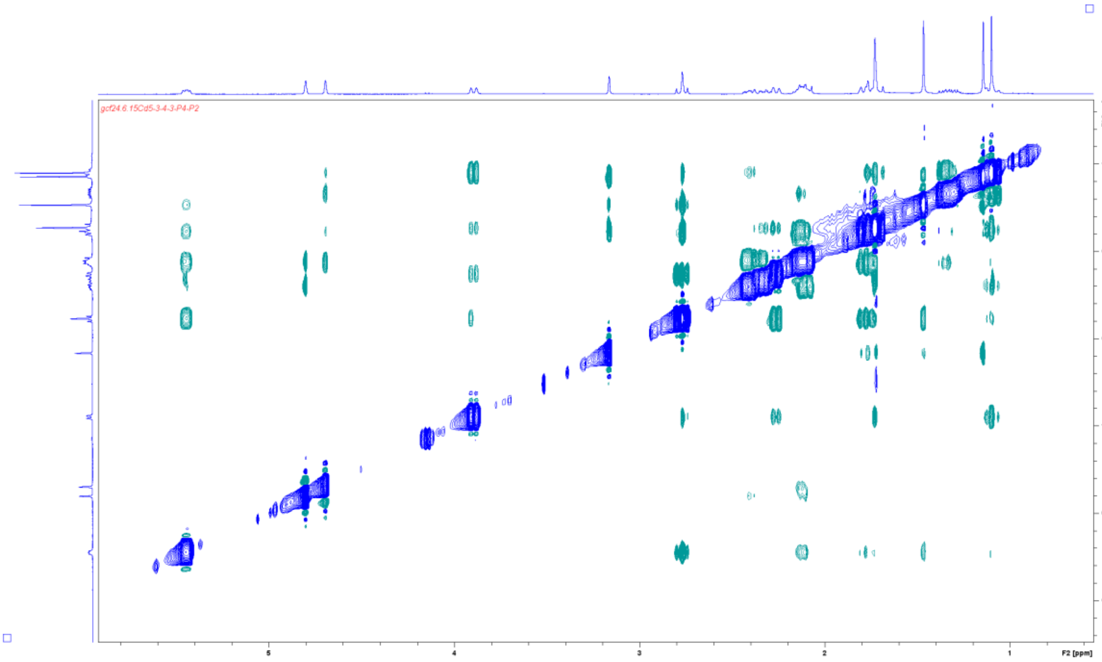

Figure S56 NOESY spectrum of compound **7** in $CDCl_3$

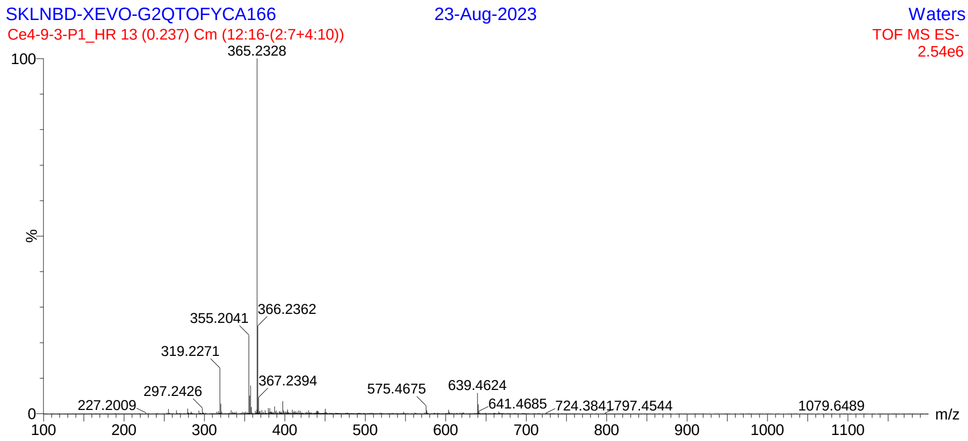

Mass	Calc. Mass	mDa	PPM	DBE	i-FIT	Norm	Conf(%)	Formula
365.2328	365.2328	0.0	-1.5	5.5	9.0	n/a	n/a	$C_{21}H_{33}O_5$

Figure S57 HRESIMS spectrum of compound **8**

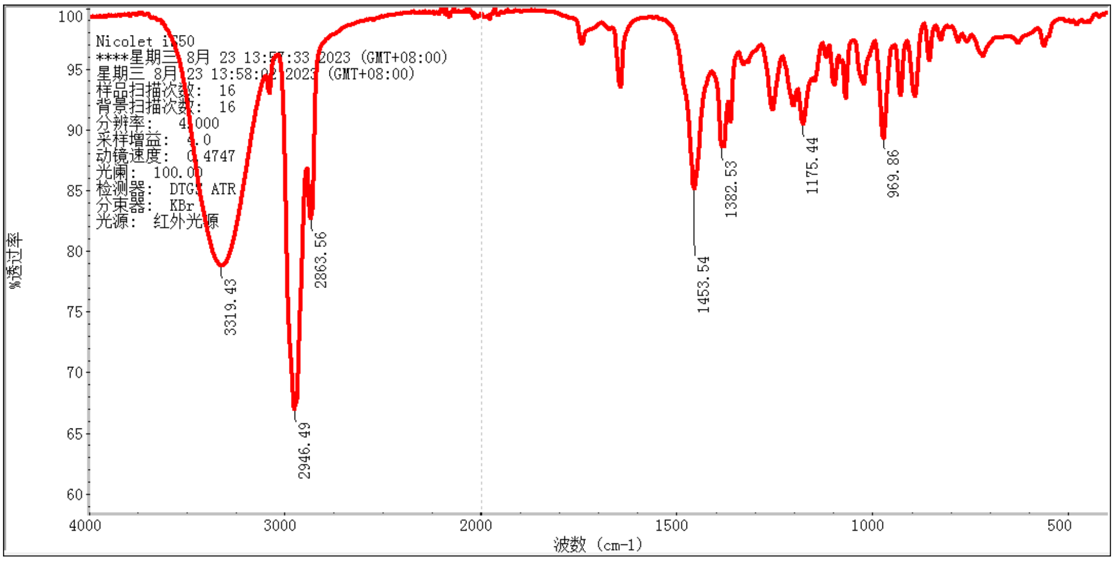

Figure S58 IR spectrum of compound **8**

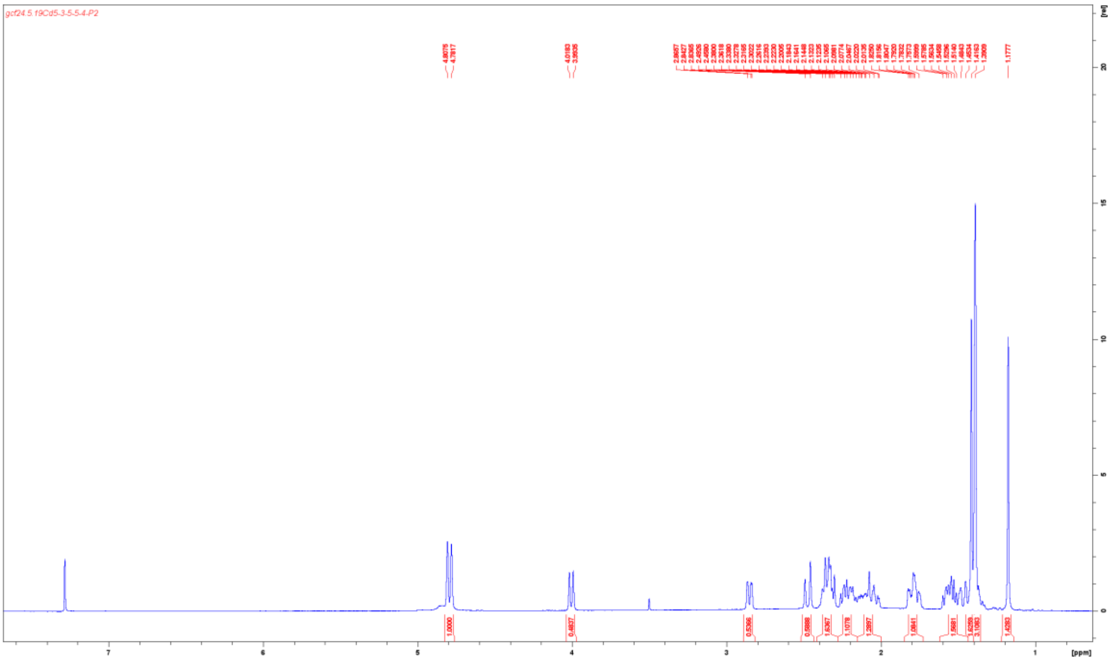

Figure S59 ^{1}H NMR spectrum of compound **8** in $CDCl_3$

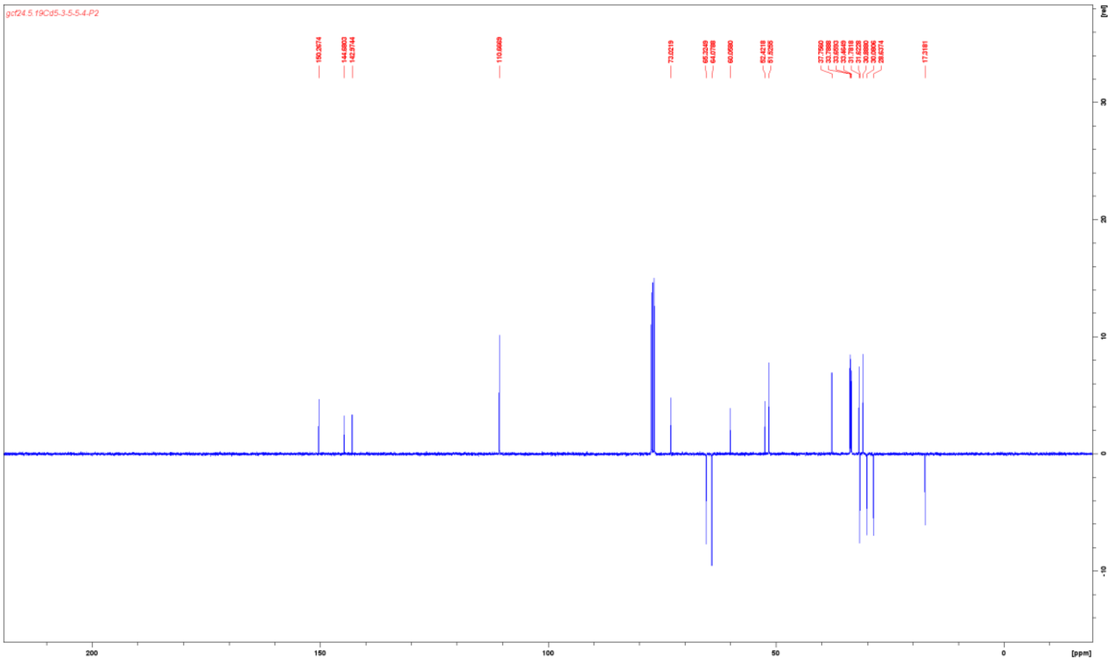

Figure S60 ^{13}C NMR spectrum of compound **8** in $CDCl_3$

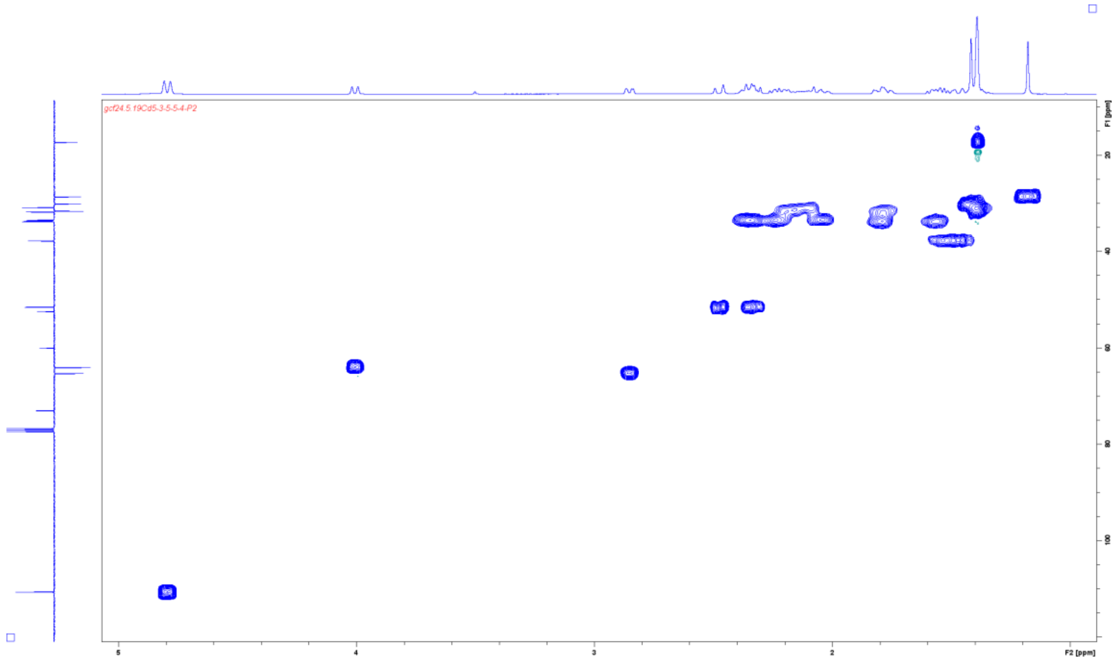

Figure S61 HSQC spectrum of compound **8** in $CDCl_3$

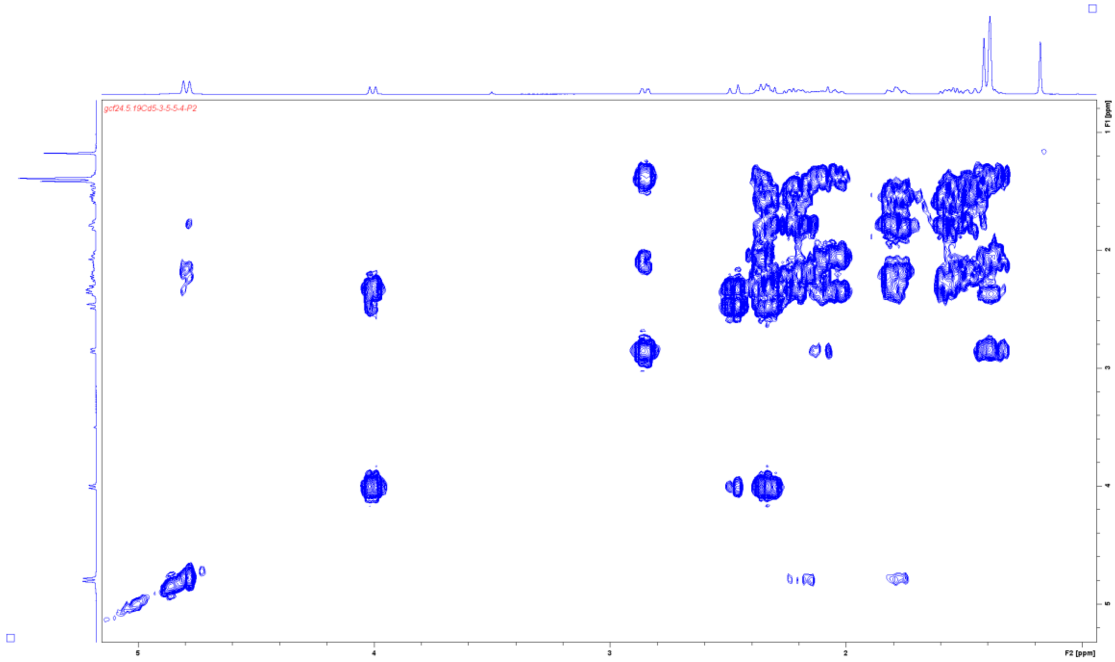

Figure S62 ^{1}H-^{1}H COSY spectrum of compound **8** in $CDCl_3$

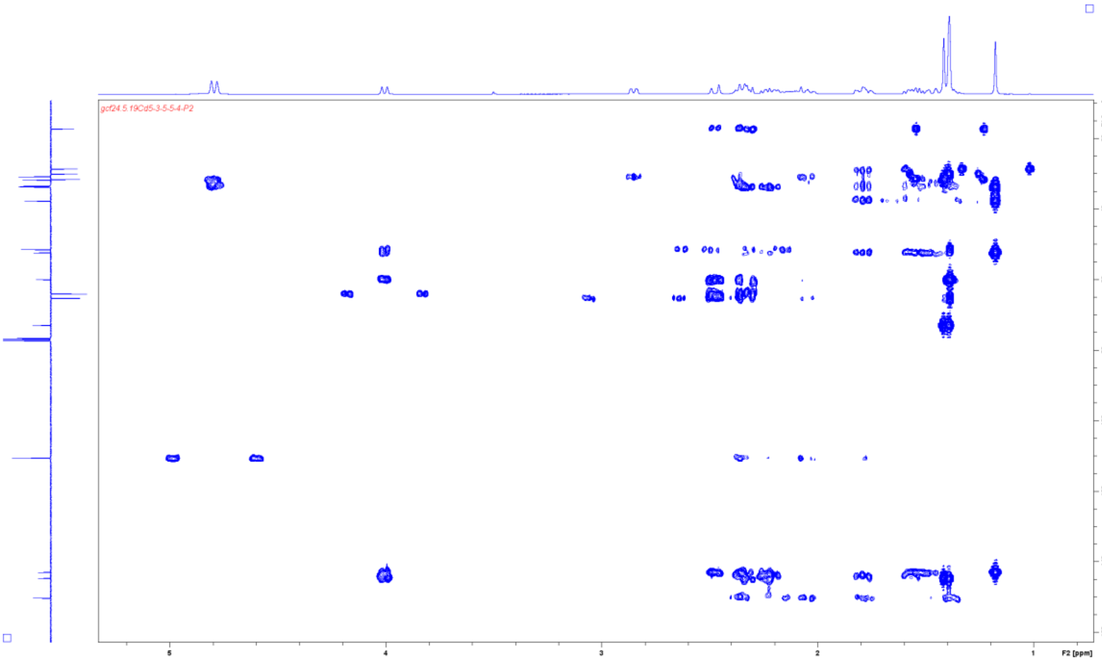

Figure S63 HMBC spectrum of compound **8** in $CDCl_3$

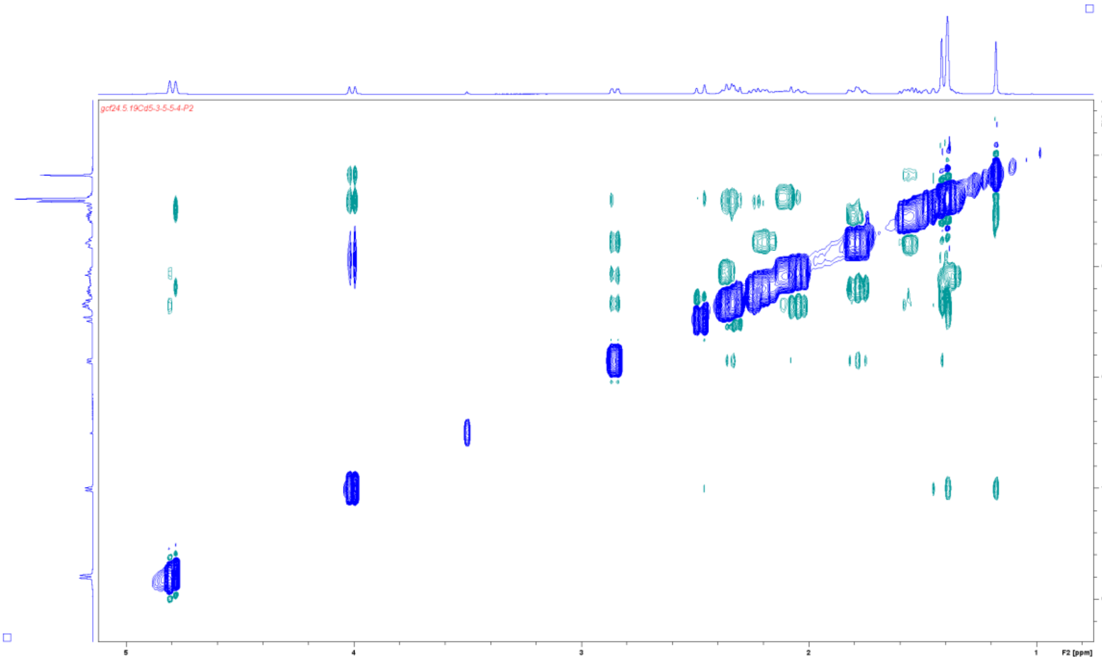

Figure S64 NOESY spectrum of compound **8** in $CDCl_3$

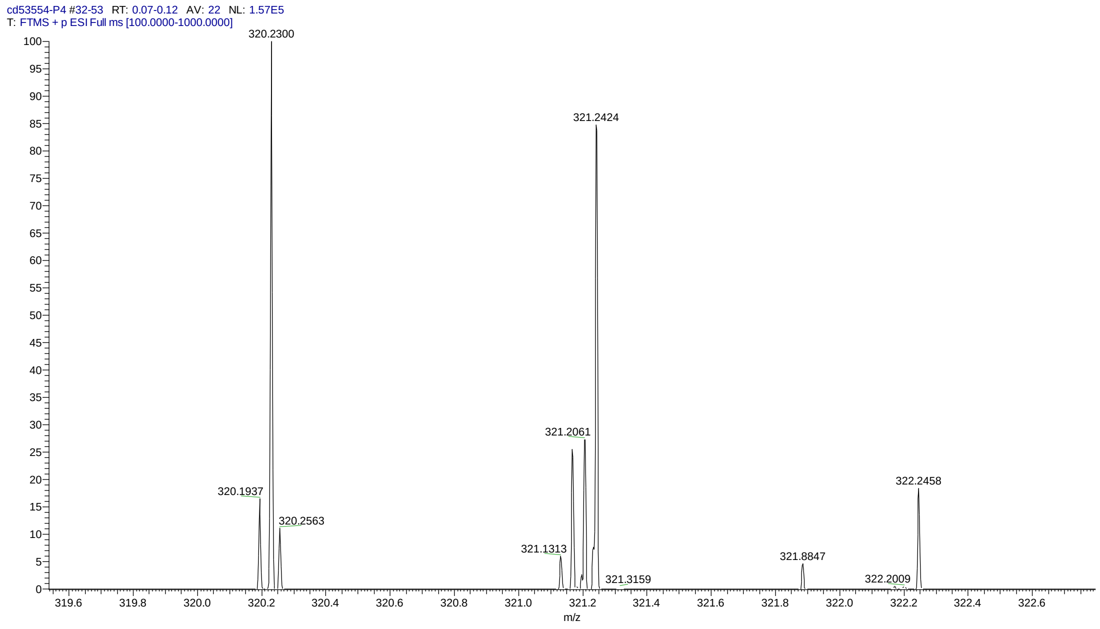

Mass	Calc. Mass	mDa	PPM	DBE	i-FIT	Norm	Conf(%)	Formula
321.2424	321.2424	0.0	-0.1	4.5	1.0	n/a	n/a	$C_{20}H_{33}O_3$

Figure S65 HRESIMS spectrum of compound **9**

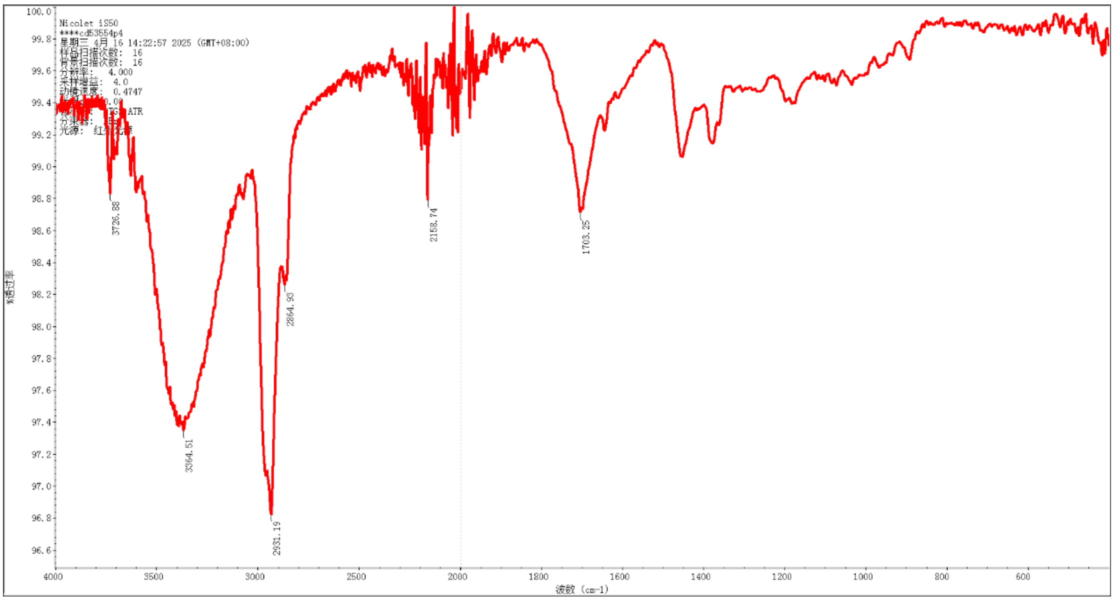

Figure S66 IR spectrum of compound **9**

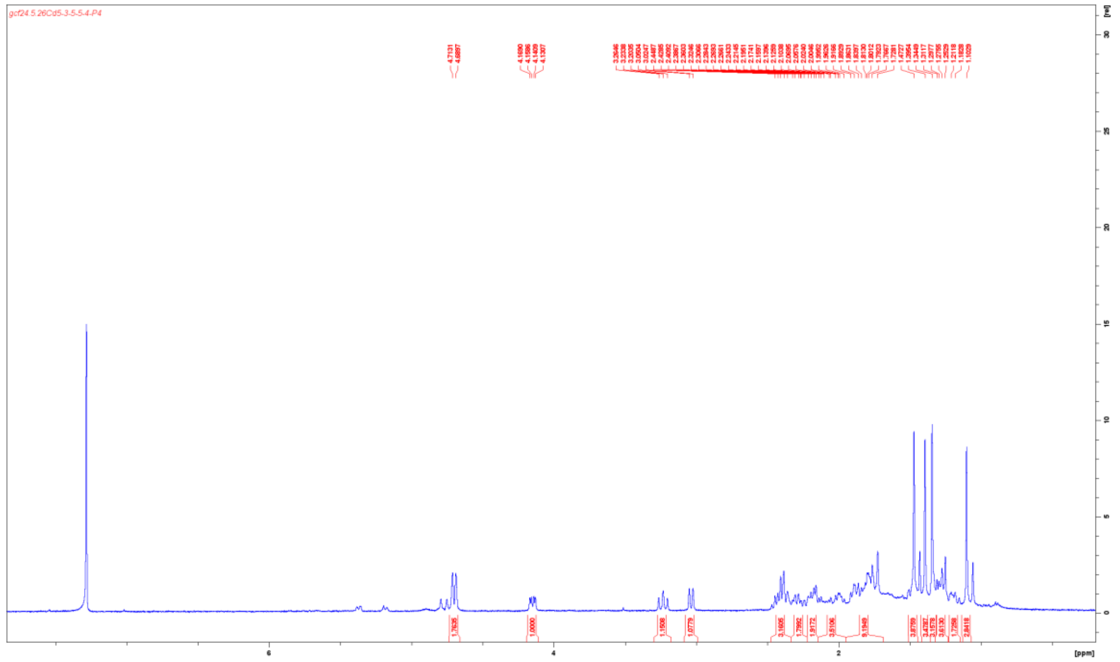

Figure S67 ^{1}H NMR spectrum of compound **9** in $CDCl_3$

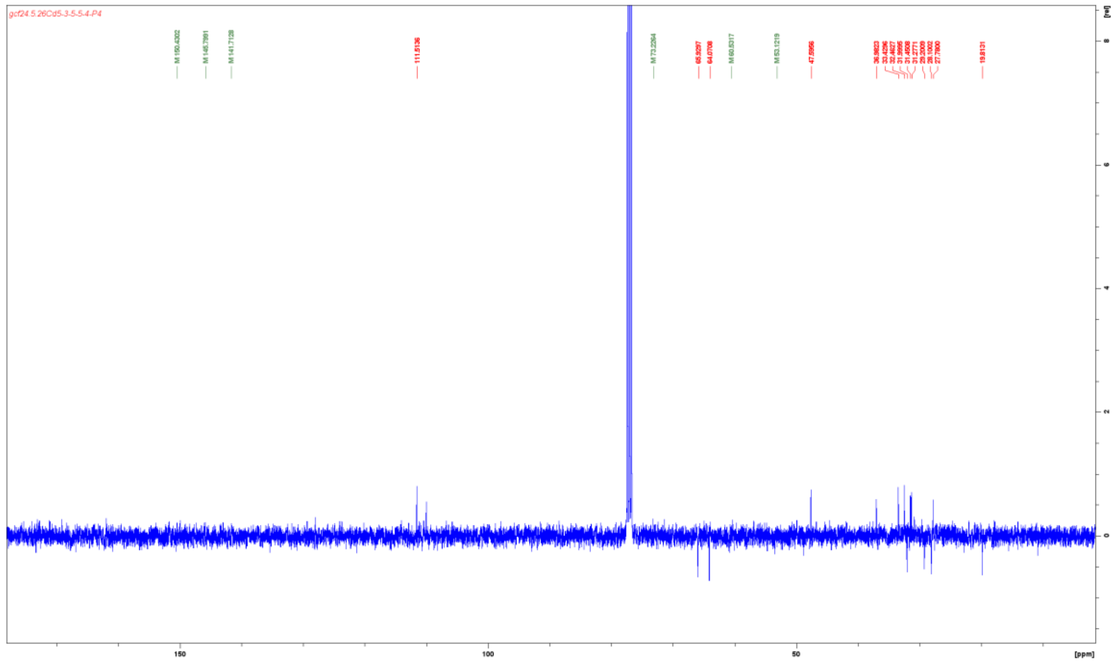

Figure S68 ^{13}C NMR (APT) spectrum of compound **9** in $CDCl_3$

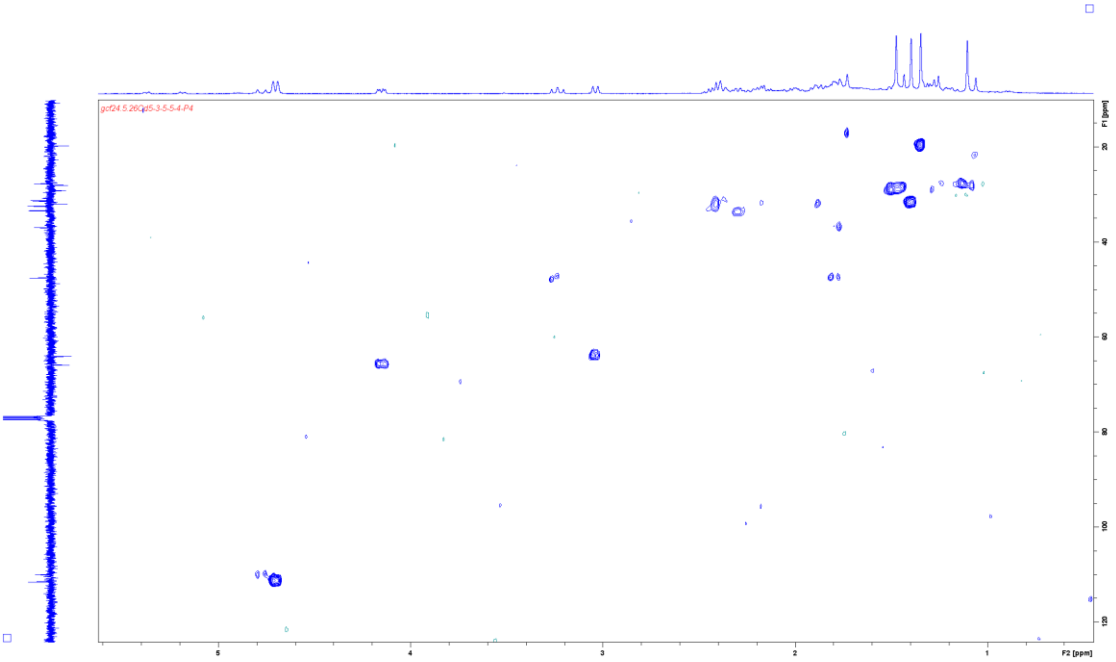

Figure S69 HSQC spectrum of compound **9** in $CDCl_3$

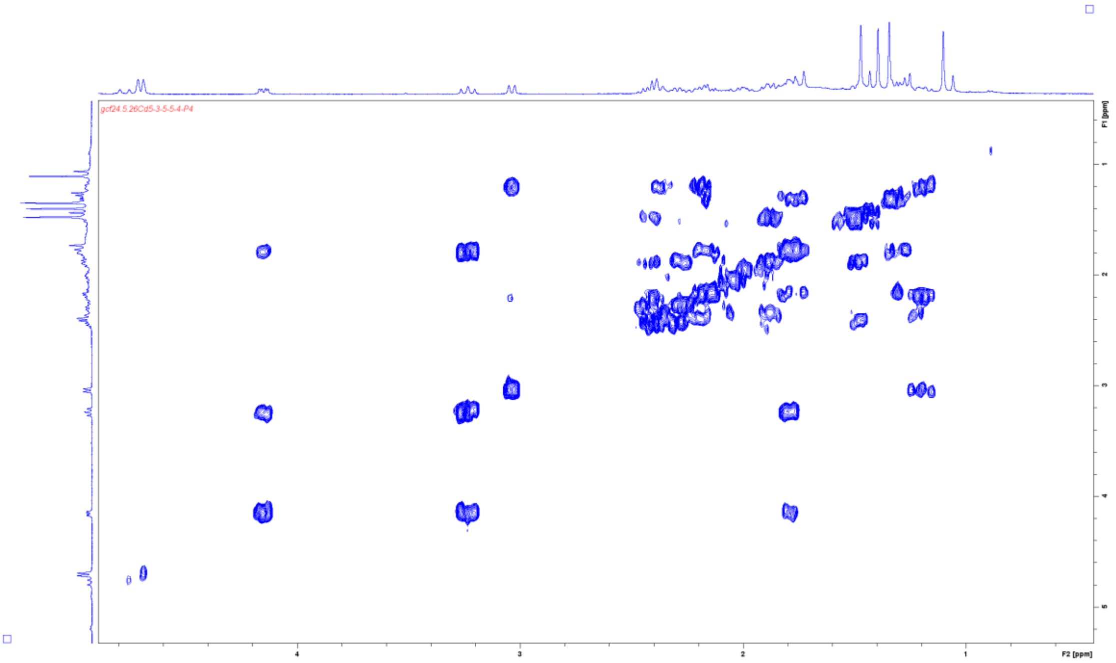

Figure S70 ^1H-^1H COSY spectrum of compound **9** in $CDCl_3$

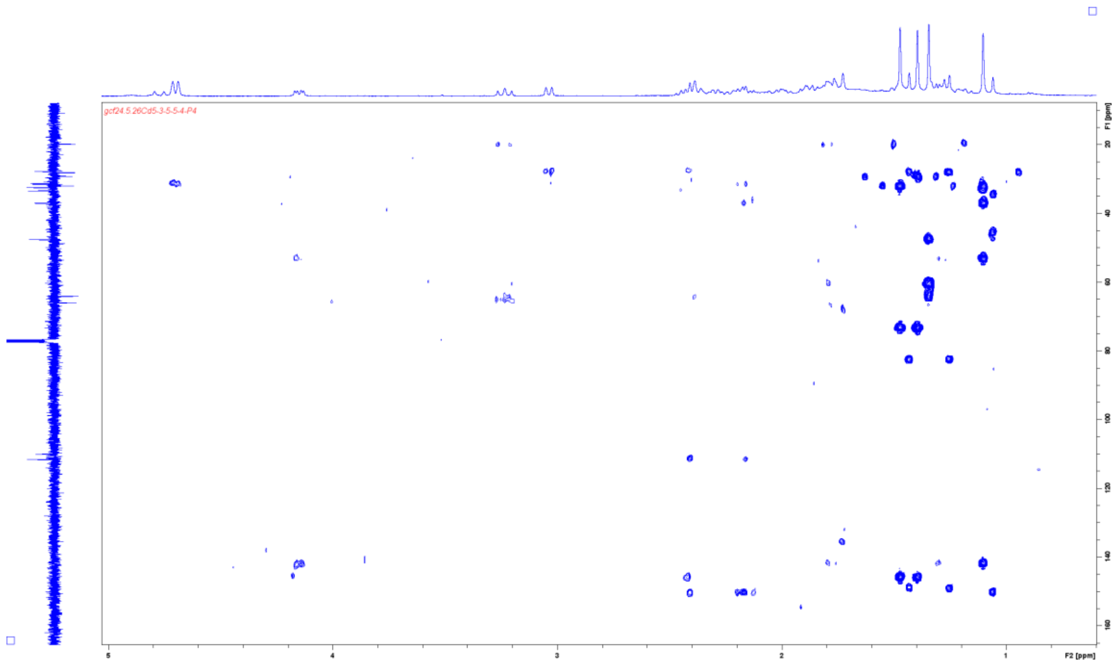

Figure S71 HMBC spectrum of compound **9** in $CDCl_3$

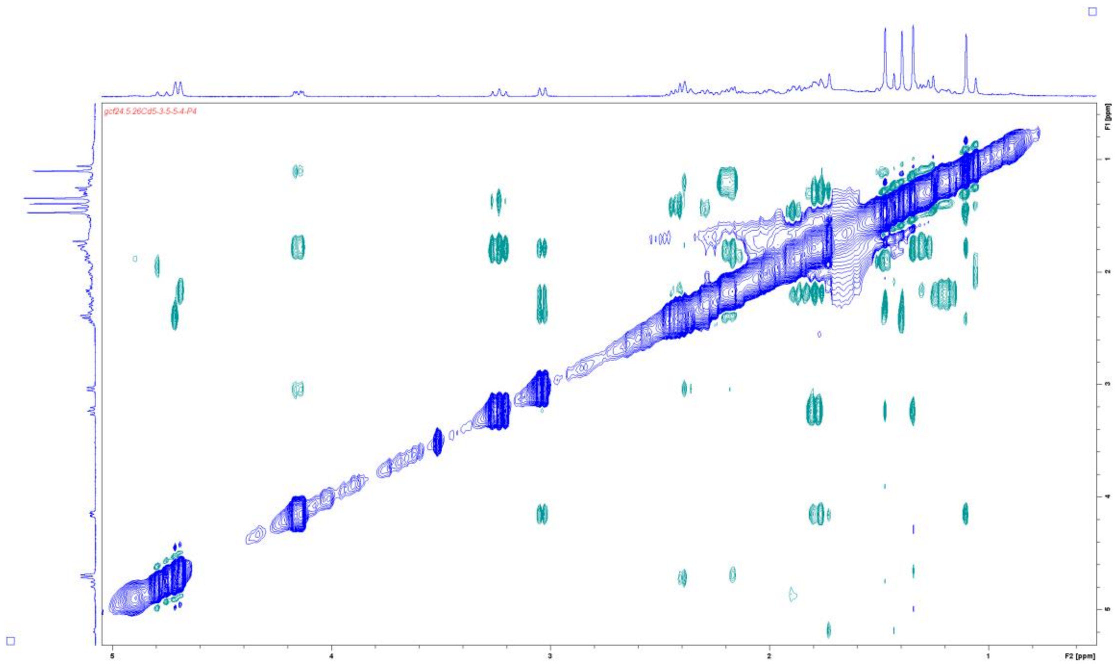

Figure S72 NOESY spectrum of compound **9** in $CDCl_3$

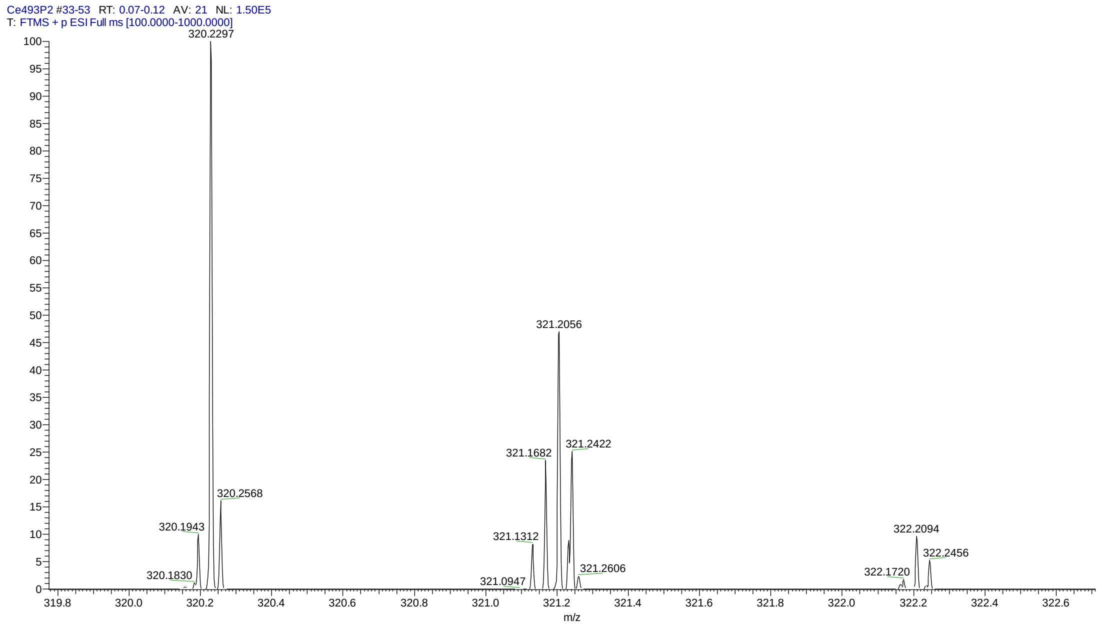

Mass	Calc. Mass	mDa	PPM	DBE	i-FIT	Norm	Conf(%)	Formula
321.2422	321.2424	-0.5	-0.7	4.5	185.7	n/a	n/a	$C_{20}H_{33}O_3$

Figure S73 HRESIMS spectrum of compound **10**

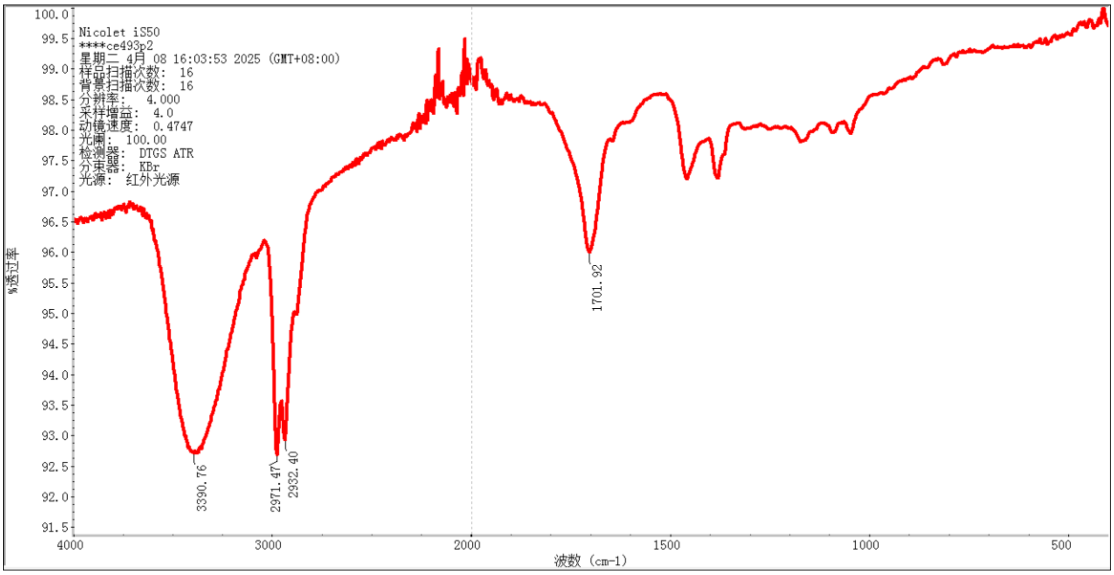

Figure S74 IR spectrum of compound **10**

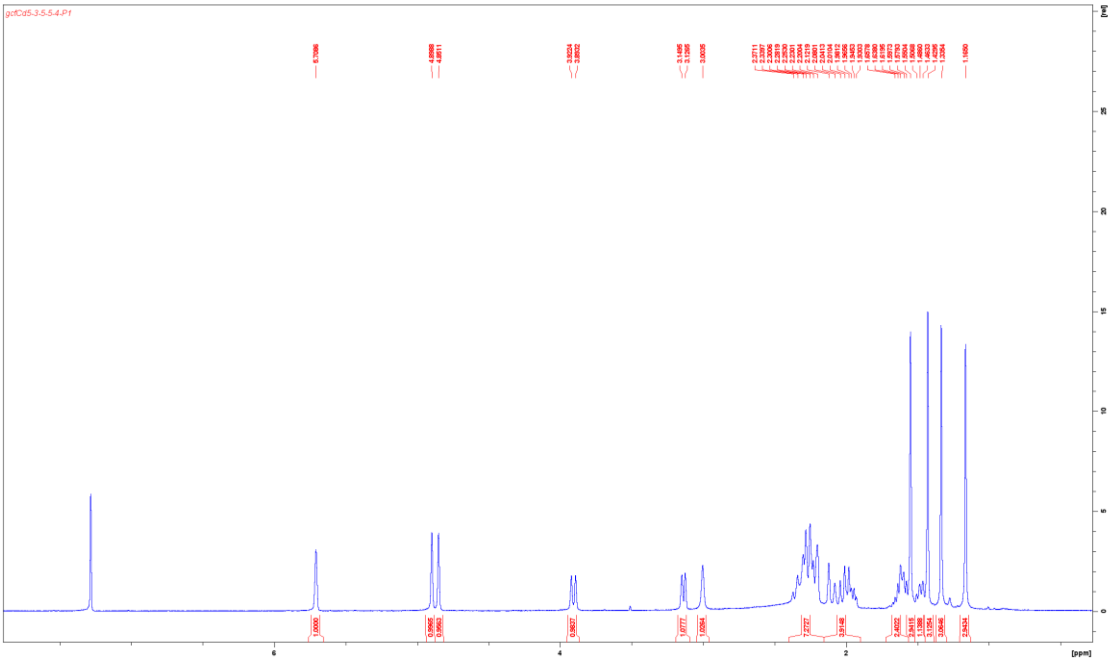

Figure S75 ^{1}H NMR spectrum of compound **10** in $CDCl_3$

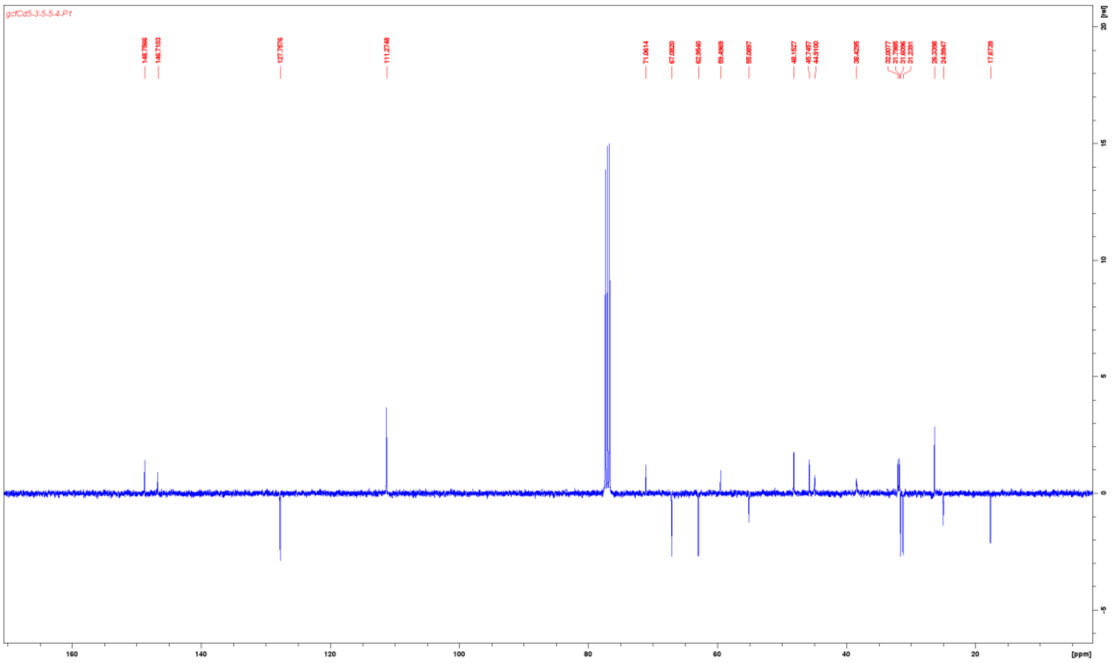

Figure S76 ^{13}C NMR (APT) spectrum of compound **10** in $CDCl_3$

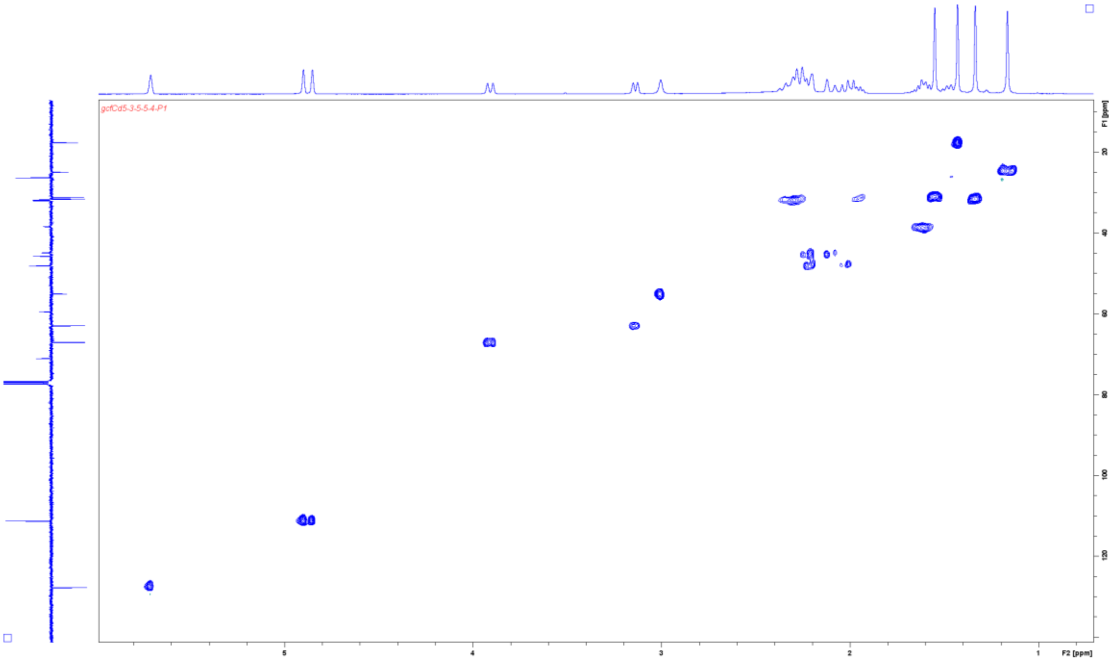

Figure S77 HSQC spectrum of compound **10** in $CDCl_3$

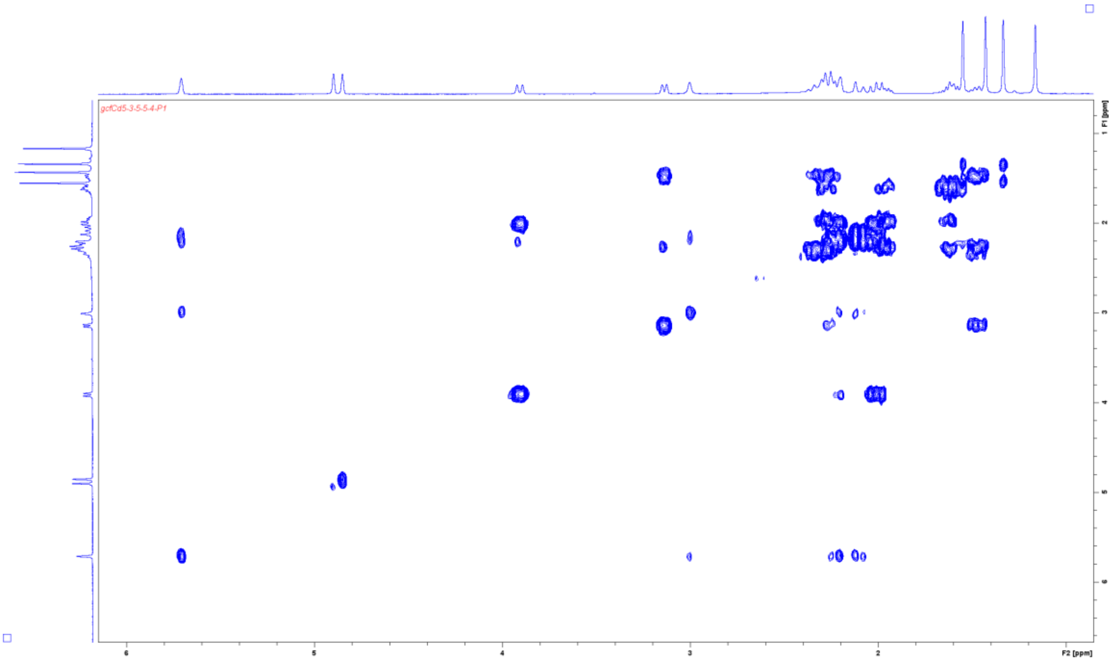

Figure S78 ^{1}H-^{1}H COSY spectrum of compound **10** in $CDCl_3$

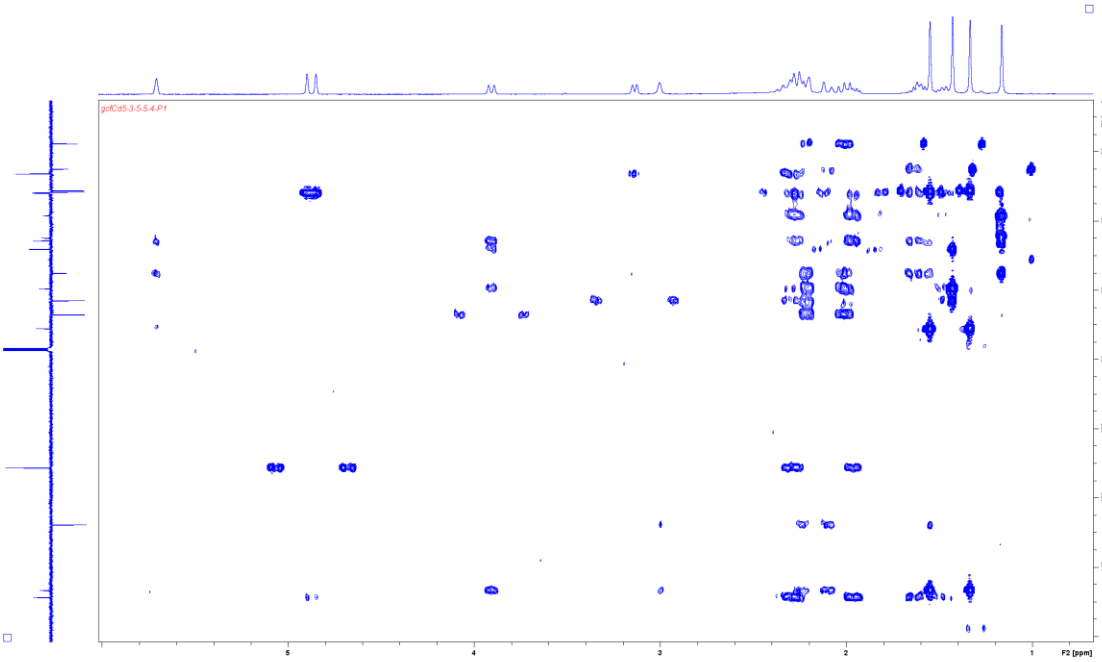

Figure S79 HMBC spectrum of compound **10** in $CDCl_3$

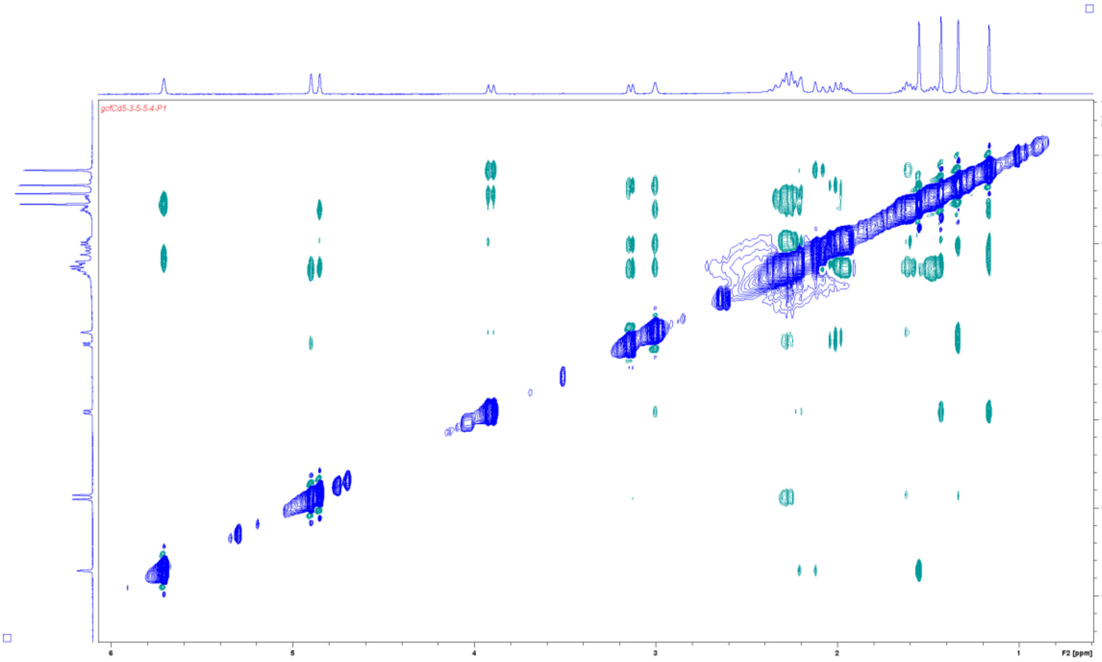

Figure S80 NOESY spectrum of compound **10** in $CDCl_3$

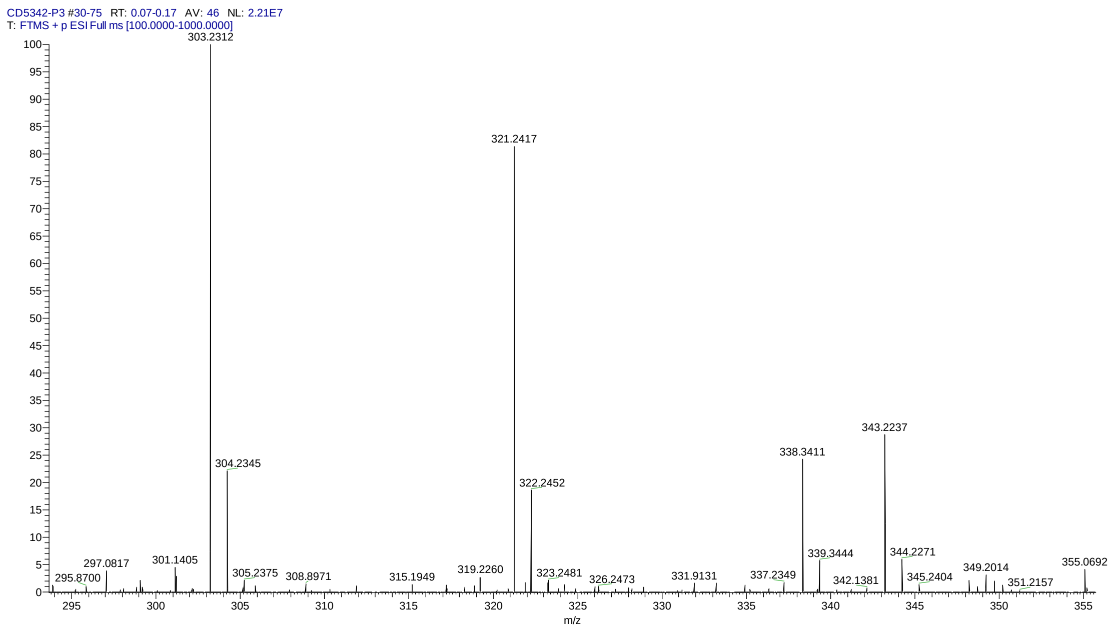

Mass	Calc. Mass	mDa	PPM	DBE	i-FIT	Norm	Conf(%)	Formula
321.2417	321.2424	-0.2	-2.2	4.5	815.6	n/a	n/a	$C_{20}H_{33}O_3$

Figure S81 HRESIMS spectrum of compound **11**

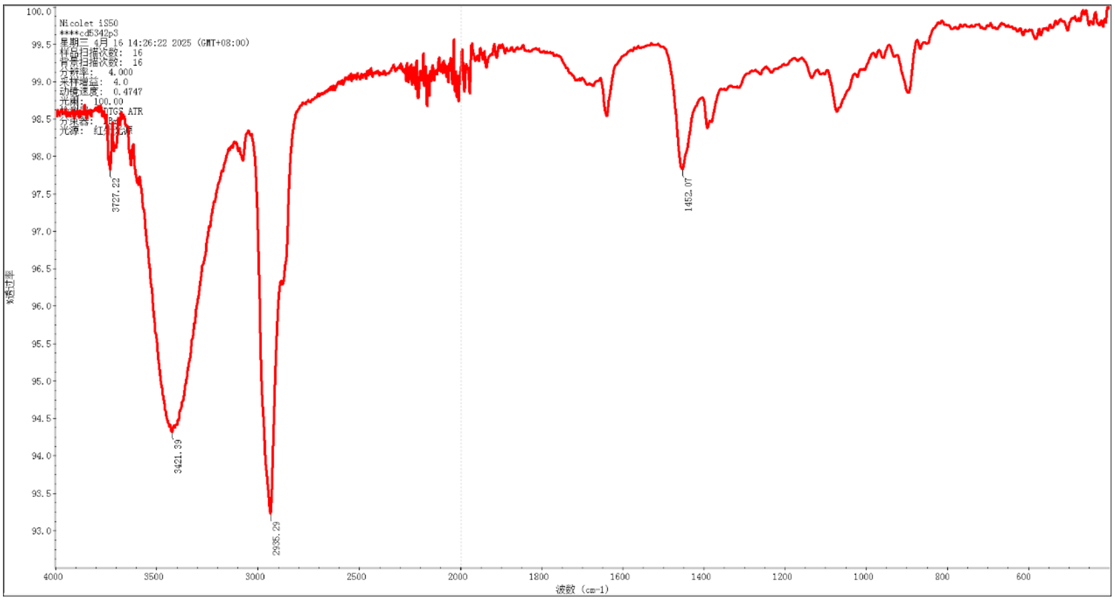

Figure S82 IR spectrum of compound **11**

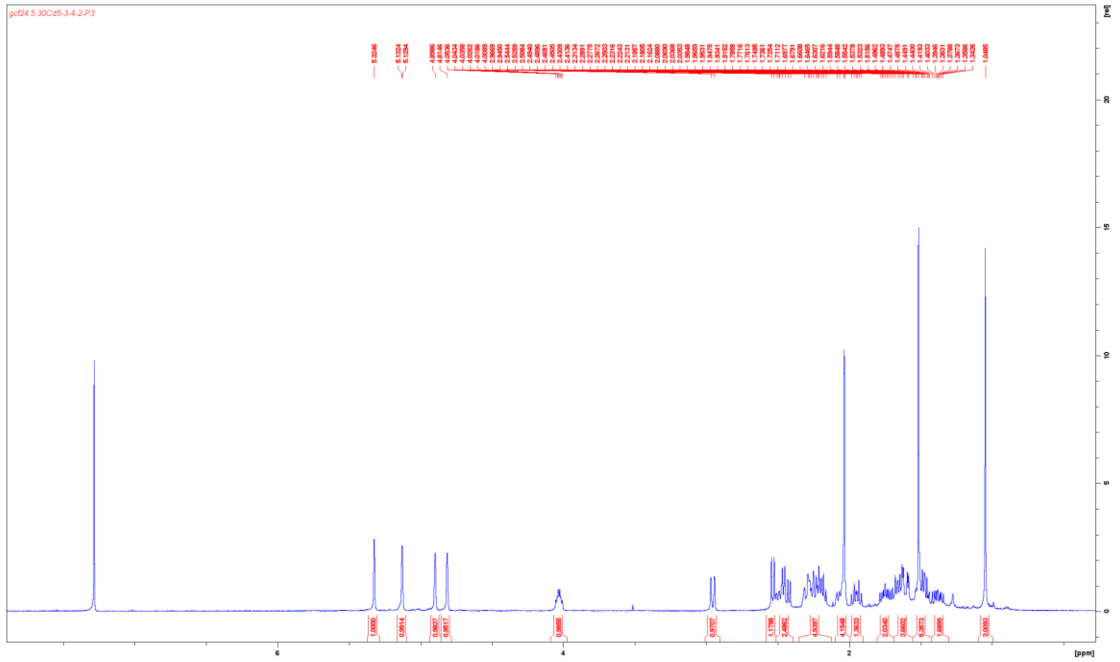

Figure S83 ^{1}H NMR spectrum of compound **11** in $CDCl_3$

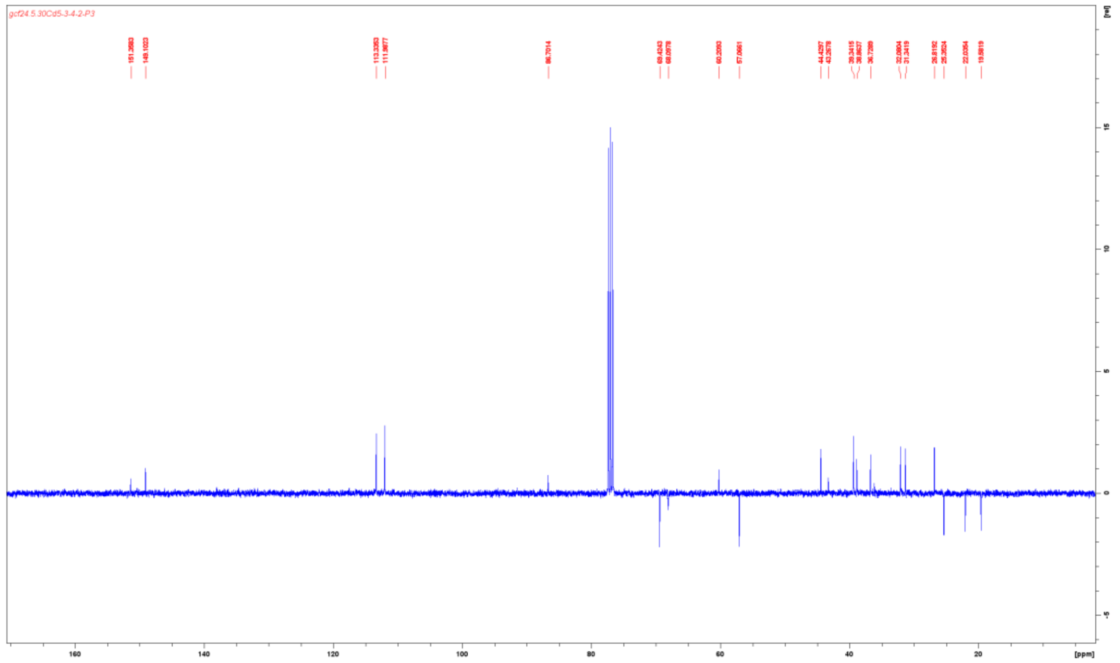

Figure S84 ^{13}C NMR (APT) spectrum of compound **11** in $CDCl_3$

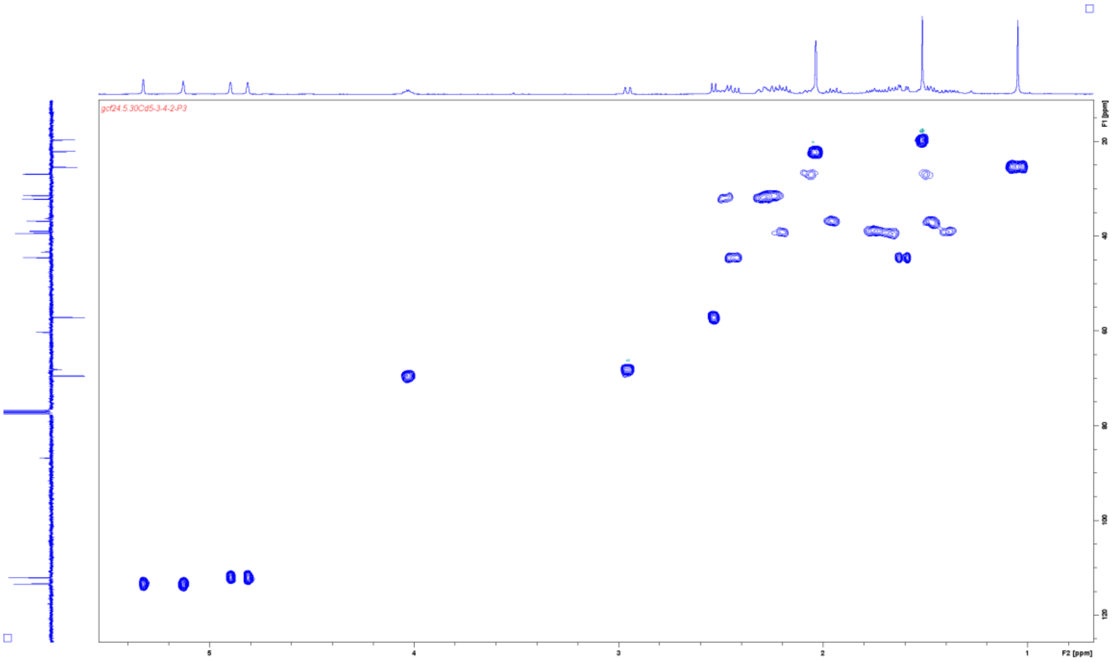

Figure S85 HSQC spectrum of compound **11** in $CDCl_3$

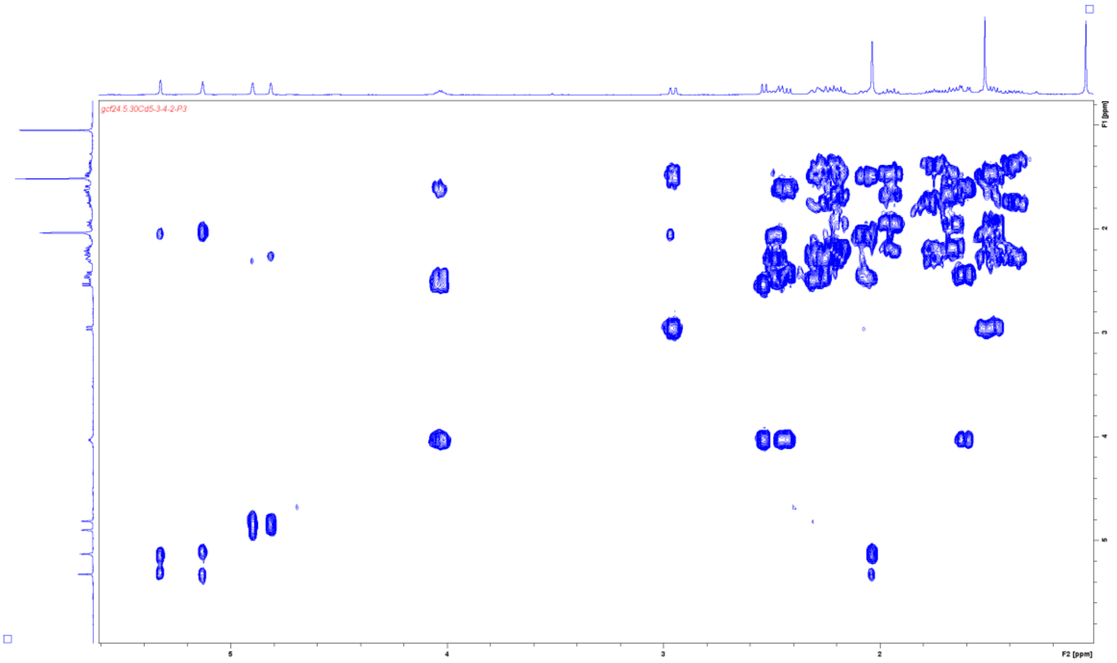

Figure S86 ^1H-^1H COSY spectrum of compound **11** in $CDCl_3$

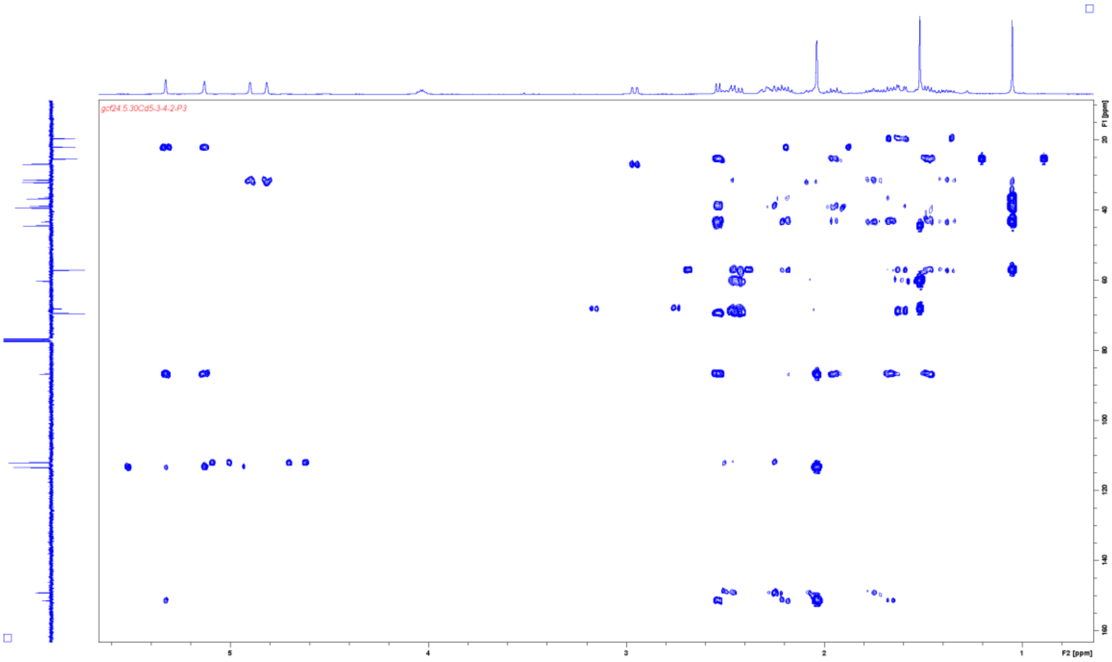

Figure S87 HMBC spectrum of compound **11** in $CDCl_3$

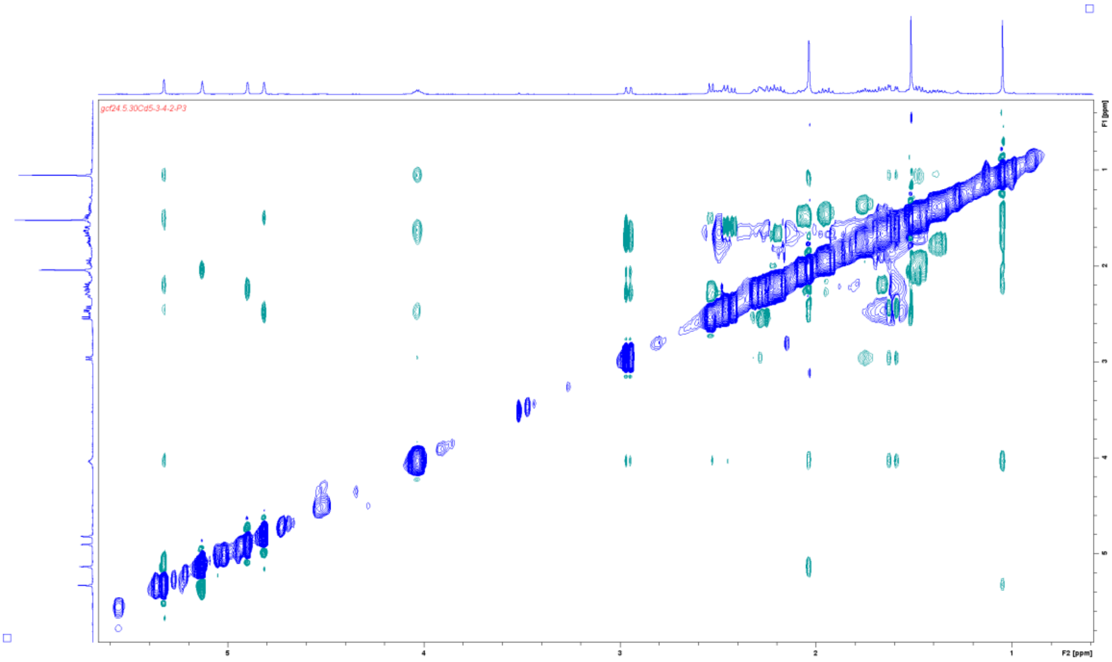

Figure S88 NOESY spectrum of compound **11** in $CDCl_3$

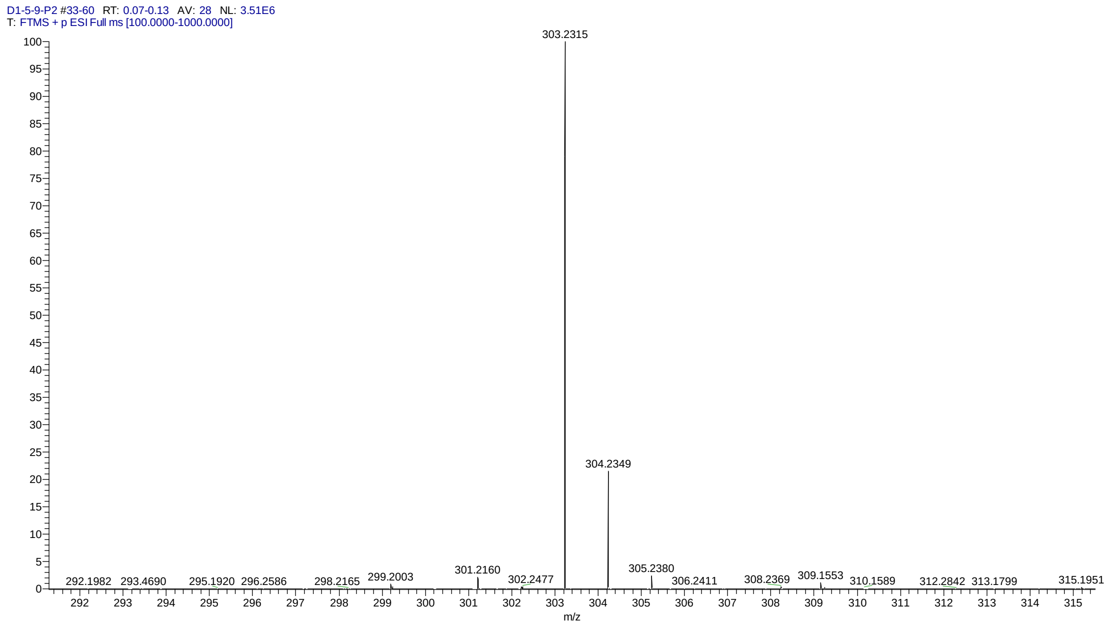

Mass	Calc. Mass	mDa	PPM	RDB	i-FIT	Norm	Conf(%)	Formula
303.2315	303.2318	n/a	-1.17	5.5	n/a	n/a	n/a	$C_{20}H_{31}O_2$

Figure S89 HRESIMS spectrum of compound **12**

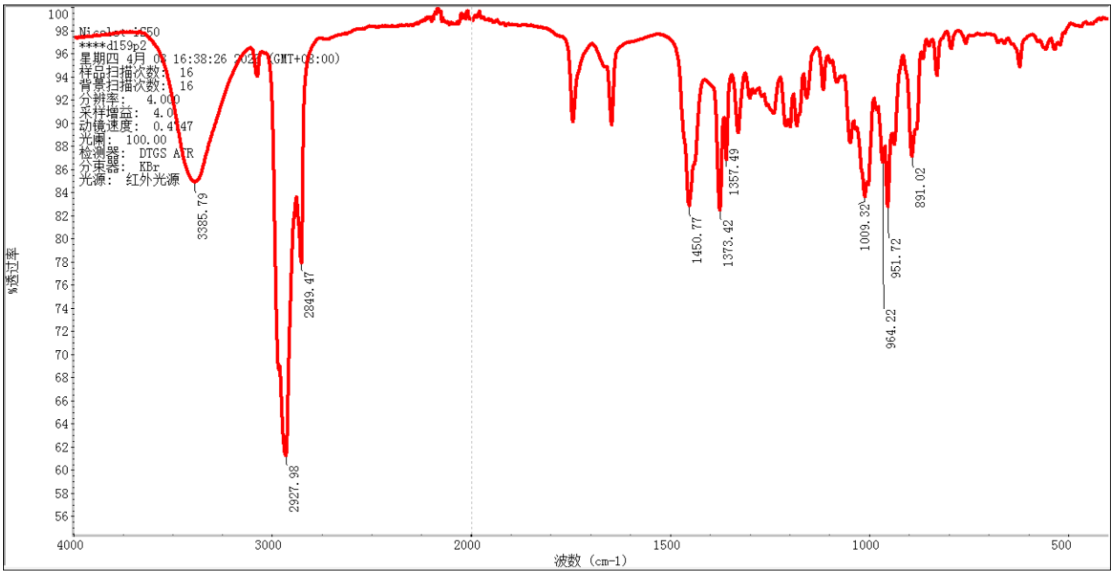

Figure S90 IR spectrum of compound **12**

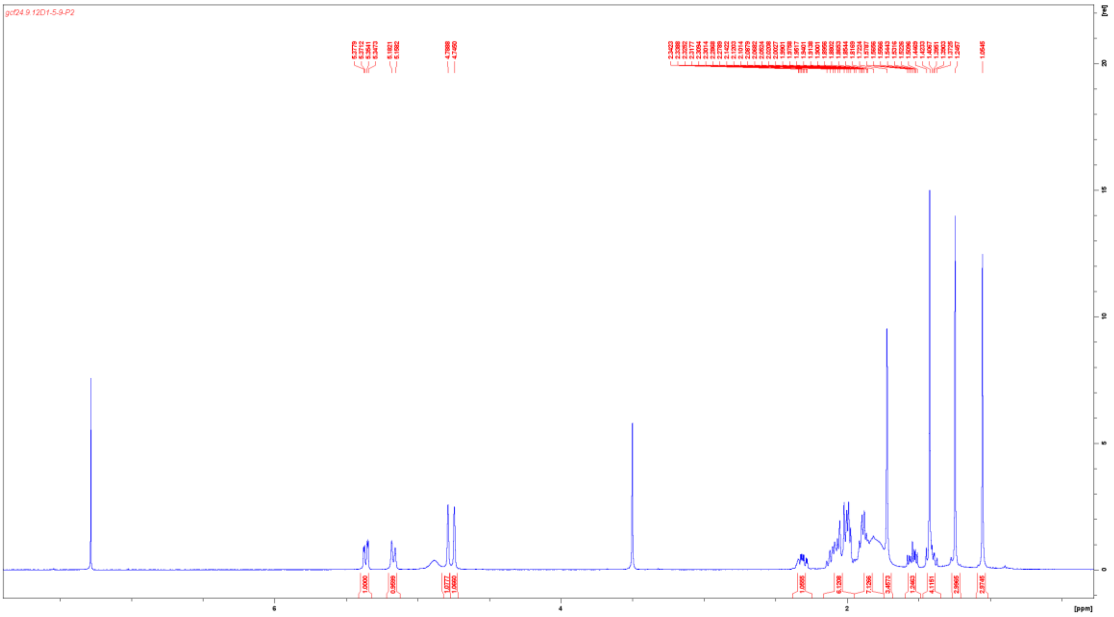

Figure S91 ^{1}H NMR spectrum of compound **12** in $CDCl_3$

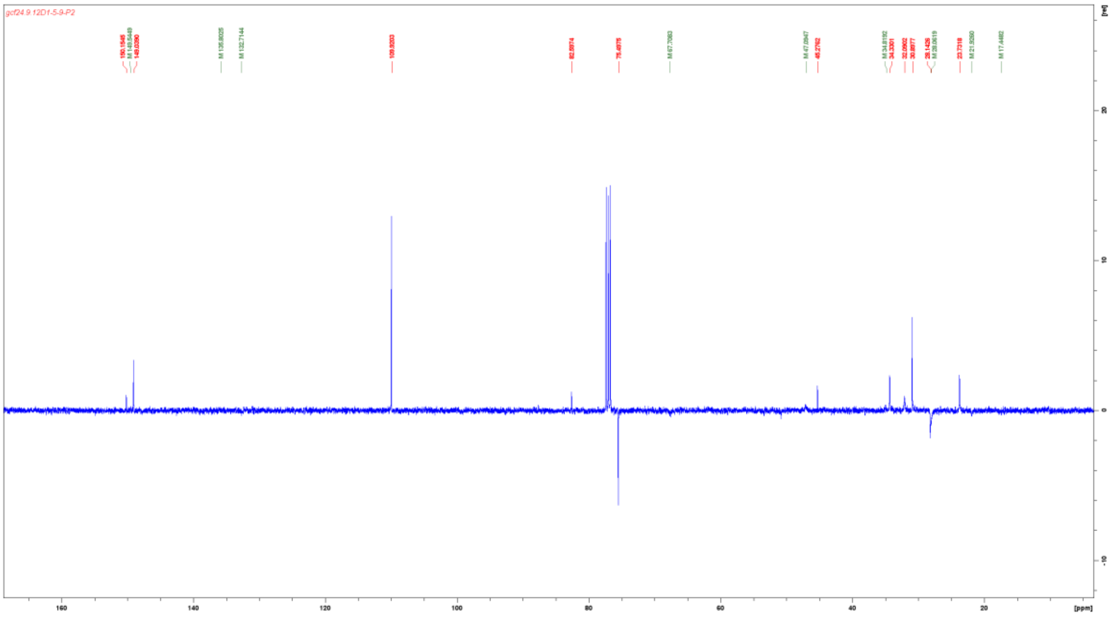

Figure S92 ^{13}C NMR (APT) spectrum of compound **12** in $CDCl_3$

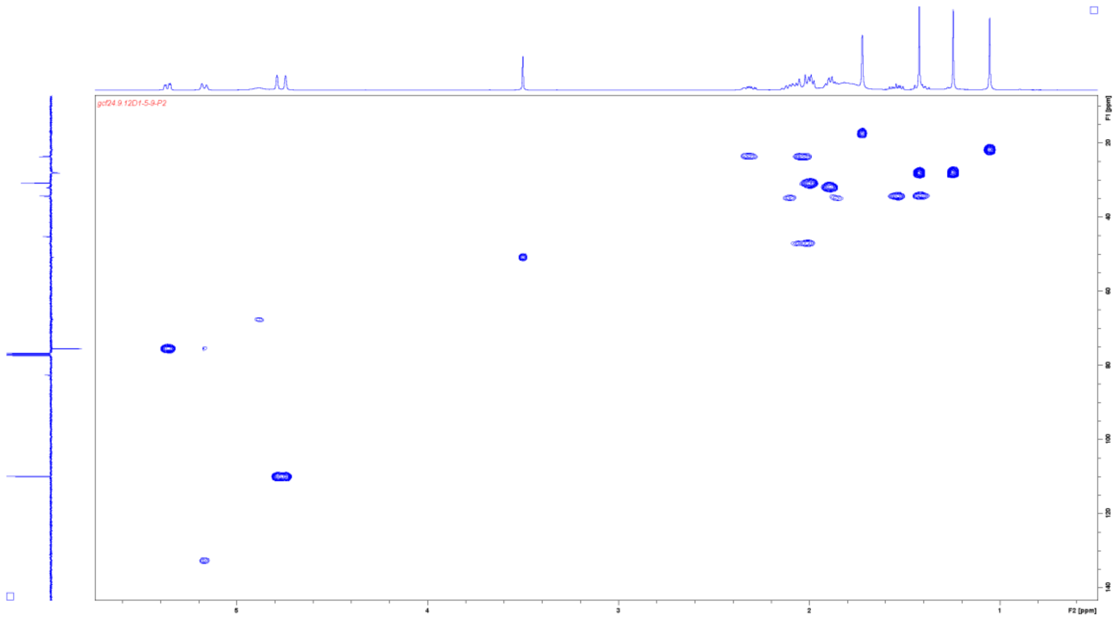

Figure S93 HSQC spectrum of compound **12** in $CDCl_3$

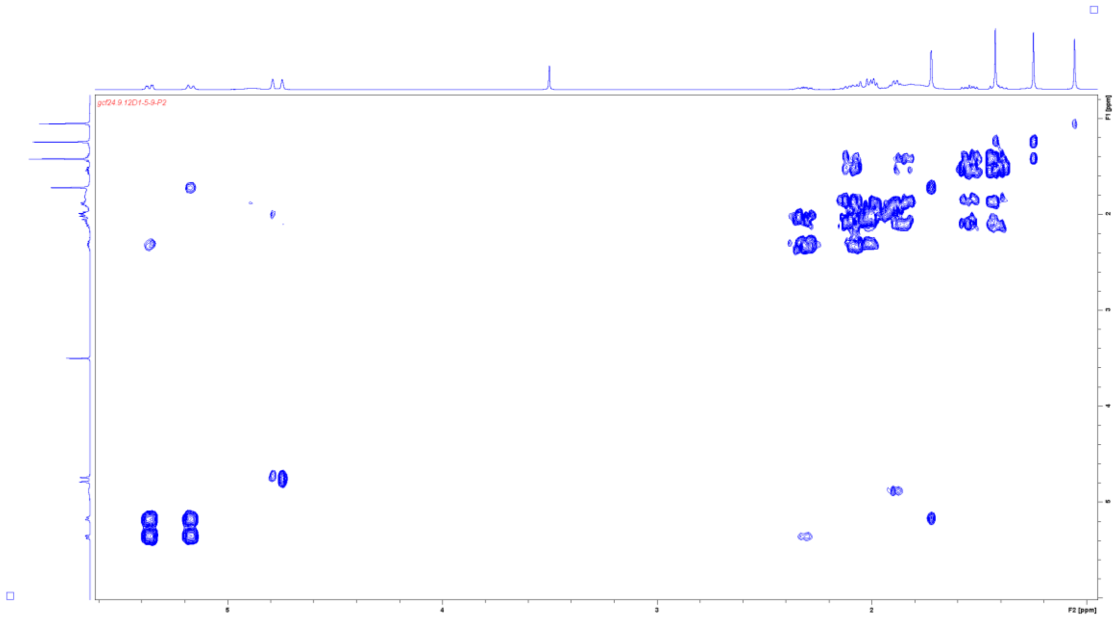

Figure S94 ^{1}H-^{1}H COSY spectrum of compound **12** in $CDCl_3$

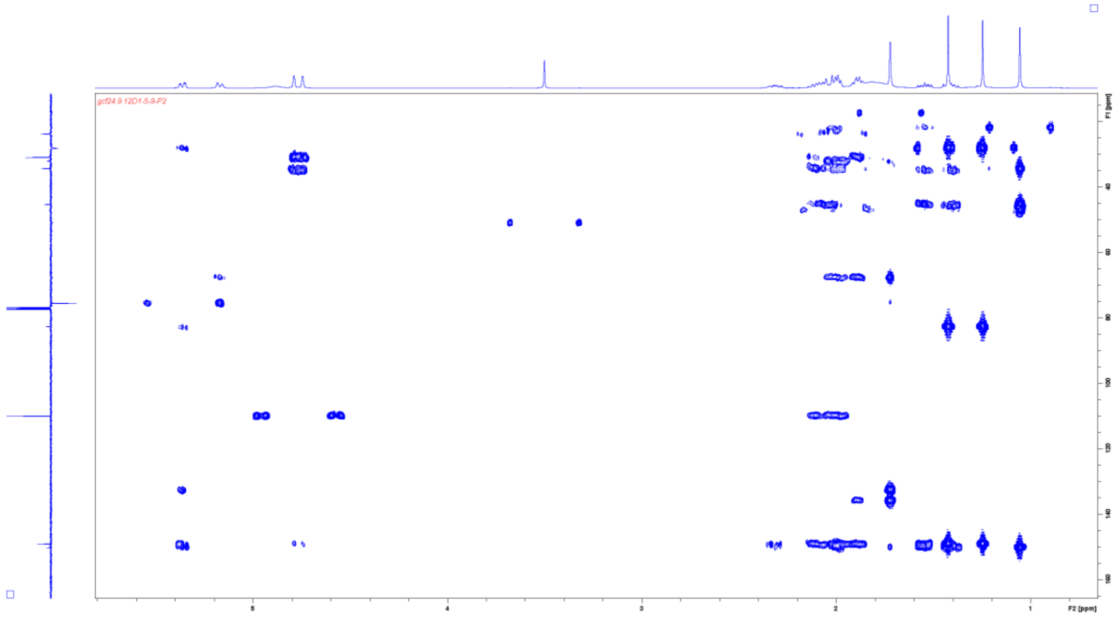

Figure S95 HMBC spectrum of compound **12** in $CDCl_3$

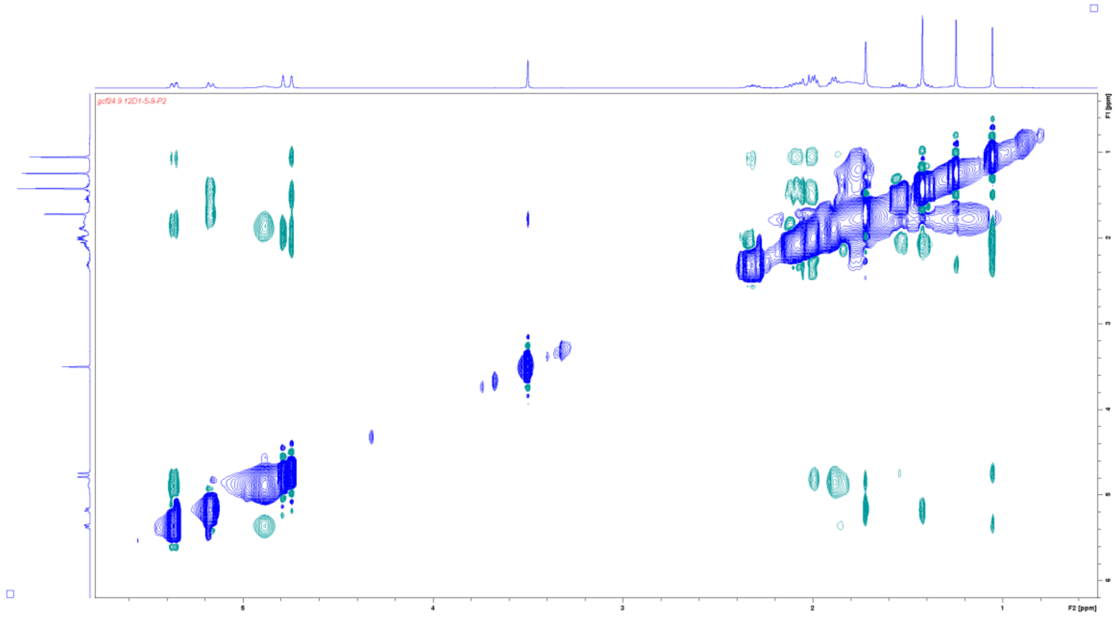

Figure S96 NOESY spectrum of compound **12** in $CDCl_3$

X-ray crystallography data of compounds 1, 2, 7, 8 and 12

Crystal of **1** (0.32 × 0.11 × 0.05 mm^3) belongs to the triclinic system, space group *P*1, with a = 8.11611(9) Å, b = 12.50127(12) Å, c = 14.02468(13) Å, α = 85.2539(8)°, β = 109.3100(10)°, γ = 75.6061(9)°, V = 1354.34(2) Å^3, Z = 3, D_{calcd} = 1.230 g/cm^3, μ(Cu Kα) = 0.672 mm^{-1}, *F(000)* = 546.0. A total of 49598 reflections were collected in the range of 7.306° ≤ 2θ ≤ 153.68°, of which 10362 unique (R_{int} = 0.0379, R_{sigma} = 0.0268) were used in all calculations. The refined structural model converged to a final R_1 = 0.0309 [$I > 2\sigma(I)$] and wR_2 = 0.0813 (all data), goodness-of-fit on $F2$ = 1.062. The absolute configuration of **1** was determined by Flack's method, in which the fractional contribution of the inverted component of its racemic twin structure was refined against data with Bijvoet pairs and expressed as Flack's parameter (zero for correct absolute configuration). The Flack's parameter for crystal of **1** was determined as 0.00(5).

The crystal of **2** (0.25 × 0.05 × 0.03 mm^3) belongs to the orthorhombic system, space group $P2_12_12_1$, with a = 6.16308(5) Å, b = 15.93927(16) Å, c = 38.1600(4) Å, α = β = γ = 90°, V = 3748.64(6) Å^3, Z = 8, D_{calcd} = 1.242 g/cm^3, μ(Cu Kα) = 0.712 mm^{-1}, *F(000)* = 1520.0. A total of 35598 reflections were collected in the range of 4.632° ≤ 2θ ≤ 156.94°, of which 7646 unique (R_{int} = 0.0440, R_{sigma} = 0.0322) were used in all calculations. The refined structural model converged to a final R_1 = 0.0329 [$I > 2\sigma(I)$] and wR_2 = 0.0836 (all data), goodness-of-fit on $F2$ = 1.018. The Flack's parameter for crystal of **2** was determined as 0.01(6).

The crystal of **7** (0.28 × 0.23 × 0.2 mm^3) belongs to the orthorhombic system, space group $P2_12_12_1$, with a = 8.28985(5) Å, b = 10.34157(8) Å, c = 20.73200(14) Å, α = β = γ = 90°, V = 1777.36(2) Å^3, Z = 4, D_{calcd} = 1.198 g/cm^3, μ(Cu Kα) = 0.615 mm^{-1}, *F(000)* = 704.0. A total of 17077 reflections were collected in the range of 8.53° ≤ 2θ ≤ 153.416°, of which 3623 unique (R_{int} = 0.0202, R_{sigma} = 0.0129) were used in all calculations. The refined structural model converged to a final R_1 = 0.0259 [$I > 2\sigma(I)$] and wR_2 = 0.0676 (all data), goodness-of-fit on $F2$ = 1.043. The Flack's parameter for crystal of **7** was determined as -0.05(4).

The crystal of **8** (0.27 × 0.2 × 0.1 mm^3) belongs to the orthorhombic system, space

group $P2_12_12_1$, with a = 8.59910(10) Å, b = 10.38500(10) Å, c = 20.9675(3) Å, $\alpha = \beta = \gamma = 90°$, V = 1872.43(4) Å^3, Z = 4, D_{calcd} = 1.137 g/cm^3, μ(Cu Kα) = 0.583 mm^{-1}, *F(000)* = 704.0. A total of 16930 reflections were collected in the range of 8.434° ≤ 2θ ≤ 153.856°, of which 3820 unique (R_{int} = 0.0399, R_{sigma} = 0.0241) were used in all calculations. The refined structural model converged to a final R_1 = 0.0338 [$I > 2\sigma(I)$] and wR_2 = 0.0922 (all data), goodness-of-fit on $F2$ = 1.068. The Flack's parameter for crystal of **8** was determined as -0.05(8).

The crystal of **12** (0.2 × 0.1 × 0.05 mm^3) belongs to the monoclinic system, space group $P2_1$, with a = 6.07376(5) Å, b = 27.7105(3) Å, c = 21.1284(2) Å, α = 90°, β = 90.5202(8)°, γ = 90°, V = 3555.92(6) Å^3, Z = 8, D_{calcd} = 1.130 g/cm^3, μ(Cu Kα) = 0.546 mm^{-1}, *F(000)* = 1328.0. A total of 147499 reflections were collected in the range of 7.63° ≤ 2θ ≤ 154.006°, of which 14502 unique (R_{int} = 0.0710, R_{sigma} = 0.0283) were used in all calculations. The refined structural model converged to a final R_1 = 0.0839 [$I > 2\sigma(I)$] and wR_2 = 0.2408 (all data), goodness-of-fit on $F2$ = 1.009. The Flack's parameter for crystal of **12** was determined as 0.02(6).
